# Comparative evolutionary analyses of eight whitefly *Bemisia tabaci *sensu lato genomes: cryptic species, agricultural pests and plant-virus vectors

**DOI:** 10.1186/s12864-023-09474-3

**Published:** 2023-07-19

**Authors:** Lahcen I. Campbell, Joachim Nwezeobi, Sharon L. van Brunschot, Tadeo Kaweesi, Susan E. Seal, Rekha A. R. Swamy, Annet Namuddu, Gareth L. Maslen, Habibu Mugerwa, Irina M. Armean, Leanne Haggerty, Fergal J. Martin, Osnat Malka, Diego Santos-Garcia, Ksenia Juravel, Shai Morin, Michael E. Stephens, Paul Visendi Muhindira, Paul J. Kersey, M. N. Maruthi, Christopher A. Omongo, Jesús Navas-Castillo, Elvira Fiallo-Olivé, Ibrahim Umar Mohammed, Hua-Ling Wang, Joseph Onyeka, Titus Alicai, John Colvin

**Affiliations:** 1 European Molecular Biology Laboratory, European Bioinformatics Institute, Wellcome Genome Campus, Hinxton, Cambridge, UK; 2 Natural Resources Institute, University of Greenwich, Chatham, Kent, UK; 3 Wellcome Sanger Institute, Wellcome Genome Campus, Cambridge, Hinxton UK; 4 CSIRO Health and Biosecurity, Dutton Park, QLD Australia; 5 School of Biological Sciences, The University of Queensland, Brisbane, QLD Australia; 6 Rwebitaba Zonal Agricultural Research and Development Institute, Fort Portal, Uganda; 7 National Crops Resources Research Institute, Kampala, Uganda; 8 Present Address: Imperial College London, South Kensington London, UK; 9 Department of Entomology, University of Georgia, Griffin, GA USA; 10 Department of Entomology, The Hebrew University of Jerusalem, Rehovot, Israel; 11 CNRS, Laboratory of Biometry and Evolutionary Biology UMR 5558, University of Lyon, Villeurbanne, France; 12 Center for Biology and Management of Populations, INRAe UMR1062, Montferrier-sur-Lez, France; 13 Department of Entomology, Cornell University, Ithaca, NY USA; 14 Institute for Molecular Bioscience, The University of Queensland, St Lucia, QLD Australia; 15 Royal Botanic Gardens, Kew, London, UK; 16 Instituto de Hortofruticultura Subtropical Y Mediterránea “La Mayora” (IHSM-UMA-CSIC), Consejo Superior de Investigaciones Científicas, Málaga, Algarrobo-Costa Spain; 17 Kebbi State University of Science and Technology, Aliero, Nigeria; 18 College of Forestry, Hebei Agricultural University, Baoding, Hebei China; 19 National Root Crops Research Institute (NRCRI), Umudike, Nigeria

**Keywords:** Biological species, Genome assembly, Comparative genomics, Phylogenomics, Cladogenesis, Transposons, Endosymbiont, Horizontal genes

## Abstract

**Background:**

The group of > 40 cryptic whitefly species called *Bemisia tabaci *sensu lato are amongst the world’s worst agricultural pests and plant-virus vectors. Outbreaks of *B. tabaci s.l.* and the associated plant-virus diseases continue to contribute to global food insecurity and social instability, particularly in sub-Saharan Africa and Asia. Published *B. tabaci s.l.* genomes have limited use for studying African cassava *B. tabaci* SSA1 species, due to the high genetic divergences between them. Genomic annotations presented here were performed using the ‘Ensembl gene annotation system’, to ensure that comparative analyses and conclusions reflect biological differences, as opposed to arising from different methodologies underpinning transcript model identification.

**Results:**

We present here six new *B. tabaci s.l.* genomes from Africa and Asia, and two re-annotated previously published genomes, to provide evolutionary insights into these globally distributed pests. Genome sizes ranged between 616—658 Mb and exhibited some of the highest coverage of transposable elements reported within Arthropoda. Many fewer total protein coding genes (PCG) were recovered compared to the previously published *B. tabaci s.l.* genomes and structural annotations generated via the uniform methodology strongly supported a repertoire of between 12.8—13.2 × 10^3^ PCG. An integrative systematics approach incorporating phylogenomic analysis of nuclear and mitochondrial markers supported a monophyletic *Aleyrodidae* and the basal positioning of *B. tabaci* Uganda-1 to the sub-Saharan group of species*.* Reciprocal cross-mating data and the co-cladogenesis pattern of the primary obligate endosymbiont ‘*Candidatus* Portiera aleyrodidarum’ from 11 *Bemisia* genomes further supported the phylogenetic reconstruction to show that African cassava *B. tabaci* populations consist of just three biological species. We include comparative analyses of gene families related to detoxification, sugar metabolism, vector competency and evaluate the presence and function of horizontally transferred genes, essential for understanding the evolution and unique biology of constituent *B. tabaci. s.l* species*.*

**Conclusions:**

These genomic resources have provided new and critical insights into the genetics underlying *B. tabaci s.l.* biology. They also provide a rich foundation for post-genomic research, including the selection of candidate gene-targets for innovative whitefly and virus-control strategies.

**Supplementary Information:**

The online version contains supplementary material available at 10.1186/s12864-023-09474-3.

## Background

The whitefly *Aleyrodes tabaci* was first described by Gennadius in 1889 and due to the lack of taxonomically robust morphological differences [1–3] with other closely related species, its systematics has undergone numerous revisions, such as the synonymization of different biological species under the binomial *Bemisia tabaci*. Recent research, however, has provided unequivocal evidence that “*B. tabaci*” has been used to refer to more than 40 biological species [4–10]. ​​ In recognition of the existence of this cryptic species group, we refer to them collectively here as *Bemisia tabaci *sensu lato (Table 1). More than half of these species are damaging agricultural pests and plant-virus vectors, thus conferring upon *B. tabaci s.l.* the status of one of the world's top-100 most invasive species [11]. *B. tabaci s.l.* cause direct damage to crops through plant phloem-sap feeding, inducing phytotoxic disorders, excreting honeydew that develops sooty-molds, and transmitting almost 500 different plant virus species [12, 13].

**Table 1:** *Bemisia tabaci s.l.* current and historical names

*Bemisia tabaci s.l.* population	Common & historical names	Partial mtCO1 phylogenetic group name	Integrative-approach derived biological-species name	Biological-species genome	INSDC accession
SSA1-SG1-Ug	African cassava whitefly, Ug1	SSA1-SG1	*Bemisia tabaci* SSA1-SG1 ∪ SG2	1a	GCA_902825415.1
SSA1-SG1-Ng	African cassava whitefly, Ng1	SSA1-SG1		1b	GCA_902825425.1
SSA2-Ng	African cassava whitefly, Ug2, Okra biotype, Sub-Saharan VI, S biotype	SSA2	*Bemisia tabaci* SSA2 ∪ SSA3	2a	GCA_903994125.1
SSA3-Ng	African cassava whitefly, African non-silver leafing 1 &2 (AnSL1, AnSL2)	SSA3		2b	GCA_903994115.1
Asia II-5	Indian cassava whitefly	Asia II-5	*Bemisia tabaci* Asia II-5	3	GCA_903994105.1
Uganda-1	Sweet-potato whitefly, Ug8, Bemisia Uganda1	Uganda-1	*Bemisia tabaci* Uganda-1	4	GCA_903994095.1
*B. argentifolii*	Silverleaf whitefly, MEAM1, cotton whitefly, sweet-potato whitefly, B and B2 biotypes	Middle East-Asia Minor 1 (MEAM1)	*Bemisia argentifolii*	5	GCA_001854935.1
*B. tabaci *sensu stricto	*Aleyrodes tabaci*, tobacco whitefly, MED, cotton whitefly, Q1, Q2 biotypes	Mediterranean (MED)	*Bemisia tabaci* s.s	6	GCA_003994315.1

The *B. tabaci s.l.* populations used here and the names associated with them historically. The union symbol “ ∪ ” is used to group populations together, under an integrative approach derived biological-species naming system. This integrative approach identified three biological species (1, 2 and 4) from the five African *B. tabaci s.l*. genomes presented here. The three other biological species used here are *B. tabaci* Asia II-5, *Bemisia argentifolii* and *B. tabaci *sensu stricto*.* See also Fig. 5

In East and Central Africa since the mid-1990s, the abundances of several *B. tabaci s.l.* cassava-colonizing populations have increased dramatically [14–17], driving epidemics of African cassava mosaic disease (CMD) and cassava brown streak disease (CBSD) [15, 18–21]. These diseases affect over 200 million sub-Saharan Africans who rely on cassava as their primary food source [22], causing production losses of up to 47% in nine East and Central African countries. The affected regions are expanding, resulting in hunger, recurrent famines, social instability, and annual losses of over US$1 billion [20, 23–25].

Several cassava-colonizing, phylogenetic species within *B. tabaci s.l.* have been proposed, based on their geographical separation and differences in their partial mitochondrial cytochrome oxidase 1 (mtCO1) sequences [7, 9, 10, 26] and were named using the mtCO1-marker naming framework. These include Sub-Saharan Africa 1 (SSA1 with five sub-groups, SSA1-SG1, SG2, SG3, SG4, SG5), SSA4, and SSA10 [7, 27]. SNP analyses identified six major genetic groups among African cassava whitefly populations, some of which differed from the partial-mtCO1 delineated putative species [28, 29]. In recent phylogenetic analyses combining a well-curated mtCO1 database with genome-wide SNPs, SSA4 nested within SSA2, while SSA1 comprised well-defined putative subspecies. Allopatric incipient speciation and a "hybrid zone" separating the two SSA1 groups were evident [30]. Biological differences were also found between the subgroups of *B. tabaci* SSA1, with mating compatibility between SSA1-SG1 and SSA1-SG2, but clear incompatibilities between SSA1-SG1 and SSA1-SG3, and between SSA1-SG2 and SSA1-SG3 [31]. In addition, mating and phylogenomics results based on SNPs were in agreement, but inconsistent with the full mitogenome analysis [28, 30]. As such, although the partial mtCO1 gene remains a highly informative marker, it needs to be considered in addition to other evidence when assigning biological species status to *B. tabaci s.l.* populations [6, 8].

In 2016, the first draft genome of *Bemisia argentifolii*, a *B. tabaci* species also known as MEAM1 (Table 1), was published [32]. This genome consisted of 615 Mb sequence length, with a final genome N50 length of 3.2 Mb, and was annotated with 15,664 protein-coding genes (PCGs). Two years later, the genome of another *B. tabaci* species (MED/Q) was published [33], with a similar genome length of 658 Mb housed within 4,975 scaffolds, but with a scaffold N50 of 437 kb. When compared, *B. tabaci* MED/Q had a higher count of annotated genes (*n* = 20,786) than *B. argentifolii*. The annotation protocols used for both species were similar, utilizing genomic alignment evidence such as RNA-seq data and *ab-initio* prediction methods such as AUGUSTUS and GENSCAN. Such prediction methods can incorporate gene models without the requirement of aligned transcriptomic evidence. Furthermore, *B. tabaci s.s.* and *B. argentifolii* genomes had *c*. 80% of all gene models assigned with a functional annotation [32, 33], but the large discrepancy in gene count suggested a high proportion of duplicated and/or fragmented gene models.

Since the mid-1990s in East and Central Africa, the abundances of several *B. tabaci s.l.* cassava-colonizing species have increased dramatically [14–17]. The phenomenon of “super-abundant” (outbreaking) cassava *B. tabaci* SSA1 populations remains a key factor driving epidemics of African cassava mosaic disease (CMD) and cassava brown streak disease (CBSD) [15, 18–21]. Although the *B. tabaci s.s.* and *B. argentifolii* genomes are available, they have limited use for studying the genetics of the African cassava *B. tabaci* SSA1 species due to the high genetic divergence between them. More recently, the draft genome of the cassava whitefly *B. tabaci* Sub-Saharan Africa—East and Central Africa (‘SSA-ECA’) was assembled from short read Illumina data derived from > 10,000 field-collected cassava whiteflies [29]. The ‘SSA-ECA’ genome, however, has a high degree of fragmentation and was annotated using *ab-initio* prediction, RNA-Seq aligned evidence and input homology evidence derived from the original *B. argentifolii* proteome, which may have led to propagating spurious or low-supported gene models. A comparison of published *B. tabaci s.l*. genomes to ‘SSA-ECA’, shows it has the shortest overall genome length at 513.7 Mb and only slightly improved N50 of 498 kb to that of *B. tabaci s.s*.

The challenge of defining a biological species within a cryptic species group such as *B. tabaci s.l*. arises from the difficulty of categorizing it within the continuous process of evolution [34, 35], compounded by the absence of reliable morphological diagnostic characters. Molecular-marker methods have partially addressed this issue, with phylogenetic-species delimitation providing a useful framework for identifying putative biological species and their boundaries [4]. Several molecular-based species delimitation approaches have been proposed previously [9, 20–23], but the most accurate method of identifying biological species has involved multiple lines of evidence such as gene flow, ecological data and molecular markers [6, 8, 10, 36]. To improve our understanding of biological species within *B. tabaci s.l.*, we present genomic and associated experimental data sets and propose appropriate biological species names, with the intent of reducing the confusion associated with the mtCO1-phylogenetic naming system. For example, we use the biological species names of *Bemisia argentifolii* and *Bemisia tabaci *sensu stricto for *B. tabaci* MEAM1 and *B. tabaci* MED, respectively (Table 1).

Due to the lack of diverse, high-quality *B. tabaci s.l.* genomic resources and the substantial differences between currently available genomes, there was a clear need for additional high quality data, particularly for cassava-utilizing species within *B. tabaci s.l*. The genomes described herein, provide the basis for further testing of new hypotheses, aided by a universally applied annotation methodology employed for this study. Here, we report on the evolutionary adaptations underlying this group of cryptic species, as well as highlighting some genetic differences with an added emphasis on resolving hitherto unclear species delineations between genetically similar and morphologically cryptic African and non-African *B. tabaci s.l.* species. These new genomic resources shall enable additional novel biological insights and progress the development of whitefly and virus-control strategies.

## Results and discussion

### Genome sequences, annotation and orthology

#### Genome assemblies

**Fig. 1:**
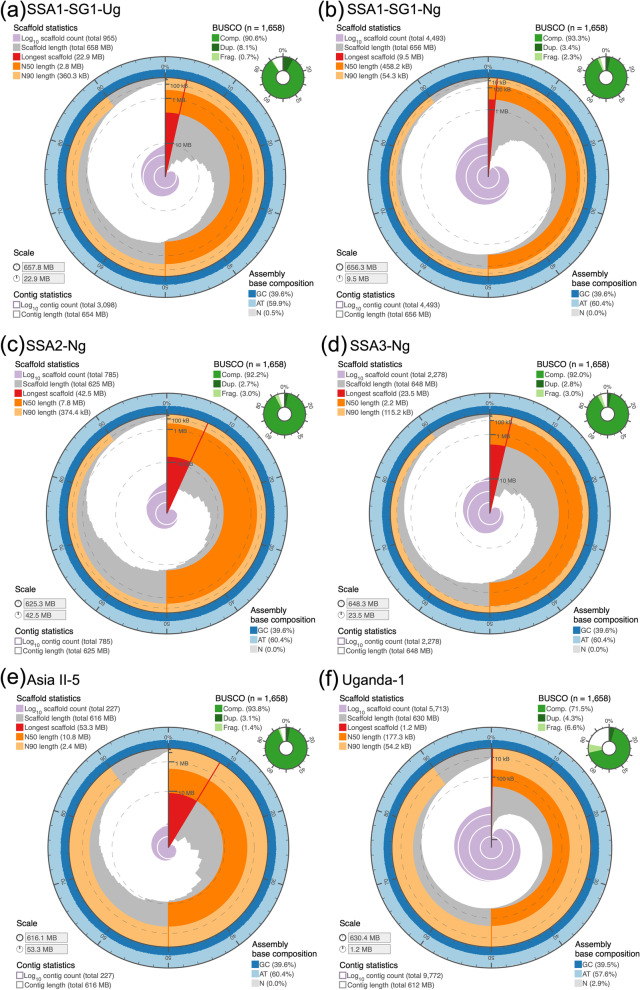
Comparison of six de novo *Bemisia tabaci s.l.* genome assemblies. Genome assemblies depicted as circular plots, where the complete plot represents the full genome length. Plots highlight the longest scaffold; scaffold N50/N90; assembly GC and gap coverage (%). Genome assembly completeness (BUSCO v3.0) shown with Insecta (OrthoDB v9; *n* = 1,658) orthology set. Historical and current *B. tabaci s.l.* population names summarized in Table 1. Assembly plots generated with assembly-stats [38]

Due to their tiny size (~ 1 mm) and considerable genome heterozygosity, we generated full-sib, inbred populations (F5-F7), for each of the *B. tabaci s.l.* populations sequenced in this study, which reduced genome heterozygosity and improved assembly graph traversal. Whitefly assemblies presented as part of this study are shown in Fig. 1; with comparisons to representative insect orders and *B. tabaci s.l.* assemblies shown in Table 2. Draft genome lengths varied (± 41 Mb), with the largest 657.7 Mb (Fig. 1a) and smallest 616.1 Mb (Fig. 1e) genomes for SSA1-SG1-Ug and Asia II-5, respectively. Although sequencing performance and genomic coverage varied between the sequenced populations, the average genome coverage of ~ 94X was used for genome assembly (Canu v1.8) [37]. Overall, assembly contiguity was improved compared to currently available whitefly genomes [29, 32, 33].

**Table 2:** Genome assembly statistics and comparison

Common name	Species/population	INSDC Accession	Assembly size (bp)	Assembly size (Mb)	Length in gap-free scaffolds (bp)	Length in gap-free scaffolds (%)	No. Scaffolds	Contig N50 (bp)	Scaffold N50 (bp)	Scaffold N50 (Mb)	L50 scaffold count	Max scaffold length (bp)	Min scaffold length (bp)	GC %
African cassava whitefly	*B. tabaci* SSA1-SG1-Ug	GCA_902825415.1	657,779,510	657.78	654,458,526	99.50	955	1,064,702	2,827,586	2.83	52	22,889,223	9,897	39.39
African cassava whitefly	*B. tabaci* SSA1-SG1-Ng	GCA_902825425.1	656,260,349	656.26	656,260,349	100.00	4,493	458,228	458,228	0.46	294	9,533,218	225	39.59
African cassava whitefly	*B. tabaci* SSA2-Ng	GCA_903994125.1	625,325,389	625.32	625,325,389	100.00	785	7,807,116	7,807,116	7.81	23	42,504,780	1,535	39.62
African cassava whitefly	*B. tabaci* SSA3-Ng	GCA_903994115.1	648,308,134	648.31	648,308,134	100.00	2,278	2,199,268	2,199,268	2.20	56	23,475,035	1,709	39.60
Indian cassava whitefly	*B. tabaci* Asia II-5	GCA_903994105.1	616,120,436	616.12	616,120,436	100.00	227	10,839,054	10,839,054	10.84	14	53,342,825	554	39.63
Sweet-potato whitefly	*B. tabaci* Uganda-1	GCA_903994095.1	630,407,635	630.41	612,247,491	97.11	5,713	91,652	177,279	0.18	1,121	1,182,621	2,406	38.36
Silverleaf whitefly	*B. argentifolii*	GCA_001854935.1	615,001,830	615.00	600,627,672	97.66	19,750	102,104	3,232,964	3.23	56	11,178,615	254	38.72
Tobacco whitefly	*B. tabaci *sensu stricto	GCA_003994315.1	658,280,412	658.28	635,234,674	96.50	4,975	54,255	436,791	0.44	421	2,857,362	221	38.27
Greenhouse whitefly	*T. vaporariorum*	GCA_011764245.1	787,368,623	787.36	785,766,123	99.79	397	500,116	69,978,400	69.97	5	97,306,886	1,000	38.91
Pea aphid	*A. pisum*	GCA_000142985.2	541,675,471	541.68	499,891,253	92.29	23,924	28,201	518,546	0.52	280	3,073,041	200	27.46
Anopheles mosquito	*A. gambiae*	GCA_000005575.1	283,349,351	283.35	260,133,033	91.81	831	169,164	49,364,325	49.36	3	61,545,105	885	40.67
Fruit fly	*D. melanogaster*	GCA_000001215.4	143,726,002	143.73	142,573,910	99.20	1,870	21,485,538	25,286,936	25.29	3	32,079,331	544	41.67
Flower beetle	*T. castaneum*	GCA_000002335.3	165,928,604	165.93	152,415,650	91.86	2,081	86,006	15,265,516	15.27	5	31,381,287	248	31.10

The *Bemisia tabaci s.l.* genome assembly statistics in comparison to selected (publicly available) insect genomes with INSDC accession numbers. Whitefly genome assembly sizes ranged from 615 to 787 Mb, which was consistently larger than the genomes of *Acyrthosiphon pisum, Anopheles gambiae, Drosophila melanogaster* and *Tribolium castaneum*

Assembly performance for the six *B. tabaci s.l.* genomes was non-uniform, with longer contig N50 values recovered for five of the six genomes (excluding Uganda-1). The mean scaffold count across all six genomes was 2,409. Asia II-5 exhibited the highest scaffold N50 (10.84 Mb) and the lowest L50 value (14) obtained in any *B. tabaci s.l.* genome published at the time of writing. Genome level contiguity improvements were achieved, with unbroken scaffold lengths considerably longer (22.8 Mb to 53.3 Mb) than that of *B. argentifolii* (11.1 Mb) and *B. tabaci s.s.* (2.85 Mb) (Table 2). Genomic GC content ranged from 38.3% (Uganda-1) to 39.6% (Asia II-5), which was broadly similar to *B. argentifolii* (38.7%) and *B. tabaci s.s.* (38.2%). Notably, the genomic GC content for all cassava-utilizing populations was > 39.4%. Genomic regions with low GC content are associated with heterochromatic DNA, which are much harder to transcribe [39]. For the Insecta orthologs (*n* = 1,367), complete BUSCO [40] recovery (OrthoDB v9 [41]; Insecta: *n* = 1,367) was between 78% (Uganda-1) and 95.1% (*B. tabaci* SSA2-Ng); with single-copy BUSCO % ranging from 72.1% to 91.7%.

Karyotype studies have shown that whitefly genomes are likely to have 10 individual chromosomes [42]. Data generated for this study, however, were insufficient to obtain telomere to telomere length assemblies, or to anchor assemblies to chromosomes. Genome assemblies generated in this work were shorter than that reported for the closely related non-*Bemisia* whitefly, *Trialeurodes vaporariorum* (787 Mb) [43], but were largely consistent with previously published *B. tabaci s.s*. and *B. argentifolii* genomes and varied between 657.7 Mb—616.1 Mb. Despite the overall improvements gained in genomic assembly of whitefly made in this study, the use of highly accurate longer reads such as PacBio HiFi, Hi-C scaffolding and optical mapping would facilitate generation of more complete and less fragmented chromosome-scale *B. tabaci s.l*. genomes. All assemblies generated in this study were deposited in the European Nucleotide Archive (ENA) at the European Molecular Biology Laboratory (EMBL-EBI) under project accession numbers PRJEB28507, PRJEB35304, PRJEB39408 (https://www.ebi.ac.uk/ena); genomic PacBio data is described in Additional file 1: Table S1. See Table 2, Additional file 1: Table S3 and Additional file 2: Figs. S2-S7 for additional assembly processing and taxonomic contamination evaluation.

#### Annotation and reannotation of *B. tabaci s.l.* genomes

Annotation and assembly quality are linked [44, 45] and, as such, assembly methodology remains the main source of variability in the recovery and comparison of gene models for all *B. tabaci s.l.* genomes. Early efforts in the genomic annotation of the new whitefly genomes in this work revealed significantly fewer gene models, compared to that of previously published *B. tabaci s.l.* (~ 12 k vs 15 k). To address this discrepancy, we applied a uniform structural annotation methodology across all *Bemisia* genomes presented herein. Our approach aimed to maximize the likelihood that the results of comparative analyses and the conclusions drawn would be reflected and underpinned by variance in whitefly biology, as opposed to the methodologies underpinning transcript model identification.

Genomic annotations of the six new *B. tabaci s.l.* genomes were performed using the ‘Ensembl gene annotation system’ [46], which produced transcript models based on empirical evidence; to the exclusion of any *ab-initio* prediction methods. High quality standards were achieved by ensuring model accuracy that derives solely via integration of alignment of expressed protein, cDNA and other types of biological sequences, such as high-throughput RNA-seq. The Ensembl gene annotation pipeline also facilitates the identification of alternative splice patterns, allowing for multiple alternate splice variants to be captured per transcript model.

Finalized gene sets obtained for all eight *B. tabaci s.l*. genomes were recovered with only small variances in total PCG counts. The number of PCGs captured across the majority of genomes were considerably fewer than previous estimates, decreasing from ~ 15 k (*B. argentifolii*) by ~ 2.5 k genes. Our analyses of PCGs recovered an average count across *B. tabaci s.l*. genomes of ~ 13 k PCGs (*n* = 13,010; stdev:510); see Table 3. For the reannotated gene sets recovered from *B. argentifolii* and *B. tabaci s.s*., the average PCG count increased to 14,316, which was likely due to the uniquely higher gene count recovered in *B. tabaci s.s*. An average, alternate-spliced transcript count of 2.06 was observed across the six new genomes, which ranged from ~ 24 k (Uganda-1) to ~ 28 K (SSA1-SG1-Ug). Reannotation of *B. argentifolii* and *B. tabaci s.s.* genomes recovered a gene-to-transcript ratio shifting away from ~ 1:1 to ~ 2:1; a feature not seen in the previously published annotations. Transcript models recovered had a mean CDS length of 1.7 kb (stdev:234), while coding exon counts averaged 212 K (stdev:38,921); see Additional file 1: Table S4 for details of input RNA-seq data generated in this study. The average exon count recovered across all PCGs was 6.9 exons per coding transcript, with an average length of 188.5 bp. Average intron length (3,157 bp) was also higher in these new genomes compared to an average of 3,061 bp seen in *B. tabaci s.s.* and *B. argentifolii.* The use of a uniform structural annotation methodology resulted in high concordance amongst all eight *B. tabaci s.l.* gene annotations.

**Table 3:** Summary of *Bemisia tabaci s.l.* structural annotation with four comparative insect genomes

		***B. tabaci* SSA1-SG1-Ug**	***B. tabaci* SSA1-SG1-Ng**	***B. tabaci* SSA2-Ng**	***B. tabaci* SSA3-Ng**	***B. tabaci* Asia II-5**	***B. tabaci* Uganda-1**	***B. argentifolii***	***B. tabaci s.s***	***T. vap.***^a^	***A. pisum***	***A. gambiae***	***D. melanogaster***	***T. castaneum***
**Gene**	Total genes	13,852	14,942	14,386	14,952	13,497	13,804	12,723	16,378	18,275	37,522	13,796	17,807	17,052
	Protein coding	12,710	13,661	12,928	13,463	12,289	12,749	12,077	15,786	18,275	36,195	13,057	13,947	16,590
**Transcript**	Total transcripts	29,757	29,919	29,035	30,073	28,862	25,101	26,475	30,266	18,275	37,522	15,718	34,920	18,996
	Protein coding	28,022	27,923	26,825	27,844	26,928	23,614	25,522	29,609	18,275	36,195	14,979	30,588	18,534
	% Prot. coding	94.17	93.33	92.39	92.59	93.30	94.08	96.40	97.83	100.00	96.46	95.30	87.59	97.57
	Avg coding length	1,893	1,805	1,604	1,575	2,019	1,383	2,255	1,506	1,382	1,983	2,508	2,280	1,765
**Exon**	Total coding exons	248,703	225,850	201,198	204,013	248,187	144,581	254,773	228,346	94,682	182,028	71,504	181,712	98,403
	Avg translatable exon length	219.12	202.2	173.69	172.47	198.64	165.05	213.73	200.71	308	208.55	371.24	378.82	288.26
	Avg exons per transcript	8.2	7.2	6.4	6.3	8.1	5.1	8.9	6.8	5.2	4.8	3.7	4.9	4.3
**Intron**	Total introns	220,681	197,927	174,373	176,169	221,259	120,967	229,251	198,737	92,160	145,833	56,525	151,124	79,869
	Avg length	3,661	3,051	3,325	3,148	3,641	2,113	3,409	2,143	1,846	1,658	1,862	1,606	1,424
	Total length	807,954,736	603,944,323	579,736,570	554,533,025	805,703,415	255,575,888	781,519,935	425,841,115	170,187,655	241,821,138	105,259,352	242,737,713	113,707,069
**5'UTR (canonical)**	Total 5'UTRs	5,496	7,587	7,572	8,017	7,398	7,218	8,670	9,130	10,877	32,115	10,118	13,394	13,522
	Avg length	5,202	3,958	4,837	3,985	5,838	1,921	4,018	1,750	192	1,661	1,841	1,441	1,086
	Complete length	28,591,304	30,027,509	36,622,299	31,951,687	43,192,086	13,862,775	34,834,074	15,973,160	2,091,440	53,344,412	18,630,451	19,296,959	14,683,753
**3'UTR (canonical)**	Total 3'UTRs	5,180	7,080	6,748	7,303	6,751	7,278	8,010	8,880	9,033	33,840	9,551	13,495	13,504
	Avg length	983	950	1,000	968	1,141	864	869	530	323	1,186	789	517	282
	Complete length	5,093,086	6,723,389	6,746,881	7,071,323	7,702,287	6,291,556	6,962,565	4,707,060	2,919,282	40,126,738	7,533,191	6,970,762	3,812,572
**BUSCO (Insecta n = 1,367)** **[%]**	Complete BUSCO	91.8	94.8	95.1	93.6	95.0	78.0	98.0	93.3	94.7	96.8	98.0	99.6	99.3
	Single copy	82.4	90.6	91.7	89.4	91.1	72.1	96.1	72.6	92.5	90.7	96.2	99.0	98.8
	Duplicated	9.4	4.2	3.4	4.2	3.9	5.9	1.9	20.7	2.2	6.1	1.8	0.6	0.5
	Fragmented	1.3	2.0	2.4	3.6	1.2	6.9	1.2	1.9	0.6	0.7	0.8	0.1	0.4
	Missing	6.9	3.2	2.5	2.8	3.8	15.1	0.8	4.8	4.7	2.5	1.2	0.3	0.3

^a^*T. vap* = *T. vaporariorum*, *D. melan.* = *D. melanogaster*

Summary statistics for the *B. tabaci s.l.* genomes and four comparative insect genomes. The new *B. tabaci s.l.* genomes and those of *B. argentifolii* and *B. tabaci s.s.* were annotated using the same methodology (see M&M). Gene features were categorized according to gene, transcript, exon, intron and both *3*’ and *5*’ UTRs. A BUSCO analysis of single copy ortholog results for orthology set (OrthoDB v9)—Insecta (*n* = 1,658) was above 82% for all cassava whitefly populations. The *B. tabaci* Uganda-1 genome had the highest percentage of missing genes from the BUSCO ortholog set. See Additional file 1: Table S3 for additional BUSCO ortholog set analyses: Arthropoda (*n* = 1,066), Metazoa (*n* = 978) and Eukaryota (*n* = 303)]

RNA-Seq evidence specifically for Uganda-1 was lacking in this study, due to the unavailability of biological sample material for sequencing. Examination of aligned evidence in support of protein-coding transcript models, however, showed gene models had RNA-Seq read coverage ranging from 62.6% (Uganda-1) to 83.2% (SSA1-SG1-Ug). Structural annotation was achieved using all combined RNA-Seq data from other *B. tabaci s.l.* generated as part of this study. Given the close evolutionary relationships of these *B. tabaci* populations, the transcriptomic evidence readily aligned and was therefore useful for capturing Uganda-1 gene features. The caveat, however, is that the Uganda-1 annotation likely has reduced gene recovery, maximization of complete ORFs, exon–intron boundaries and species-specific features.

Structural annotation of the *B. tabaci s.l.* genomes revealed a marked reduction in total gene models recovered, compared to previously published gene sets of *B. tabaci s.s*. and *B. argentifolii*. Despite this, we posit that use of a uniform annotation methodology employed across contiguous genome assemblies, and without use of *ab-initio* methods, provides a well-supported and conservative estimate of *B. tabaci s.l.* gene space. Furthermore, downstream analyses should benefit from this increased robustness, ensuring conclusions drawn are underpinned by inherent biology and not the result of disparate annotation methodologies.

**Fig. 2:**
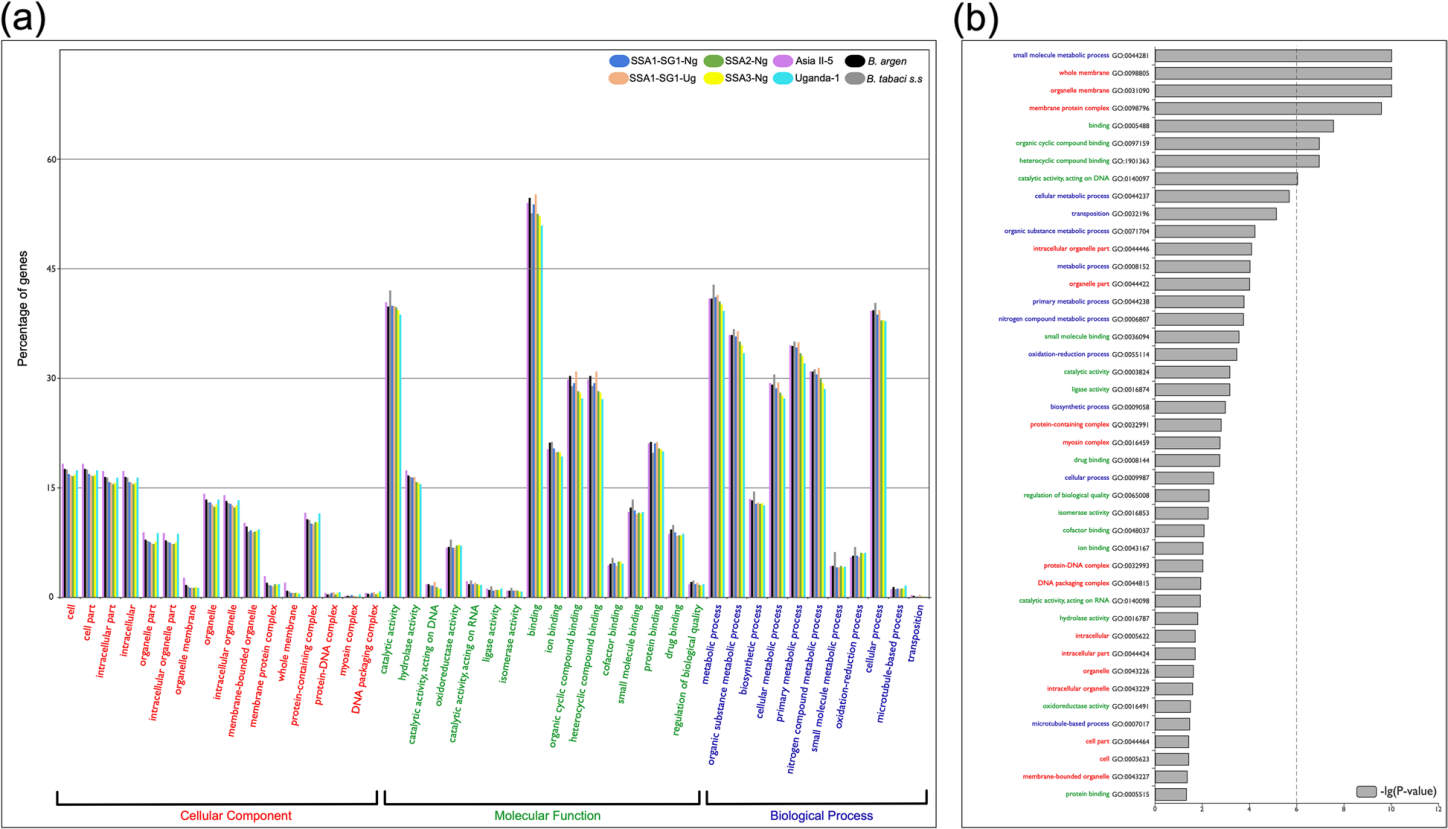
*Bemisia tabaci s.l.* functional annotation and enriched GO terms. **a** Histogram comparison of gene percentages of significant GO terms, identified from eight *B. tabaci s.l.* genomes. Population names are color coded in the inset and *B. argentifolii* has been abbreviated to “*B. argen*”. **b** Significant differences in GO terms across eight *B. tabaci s.l.* populations. Y-axis shows log.^10^ transformed *P*-values for each GO term in figure part (a). Results generated using web service WEGO v2.0 (https://wego.genomics.cn/)

Visualization and comparison of gene functional annotation between all whitefly genomes (Fig. 2) revealed that ~ 66.44% of input genes recovered some form of GO term annotation (Table 4). Some genes showed a significant difference between the expected frequencies of genes with GO terms and observed GO term frequencies; with most GO terms recovered related to molecular function (86.30%), followed by biological process (60.75%) and cellular location (30.49%). The highest proportion of GO-enriched genes across *B. tabaci s.l*. genomes were related to catalytic activity (GO:0003824) (> 41% of all PCGs) and binding (Parent GO:0005488) (> 50% of all PCGs); see Fig. 2a, b. Approximately 30% of *B. tabaci s.l.* PCGs were characterized as related to functional binding of: (i) organic cyclic compounds (GO:0097159) or (ii) heterocyclic compounds (GO:1,901,363) (Fig. 2a). Aside from the above-mentioned GO-enriched gene ontologies, other significantly different GO-terms between whitefly genomes related to forms of binding; including small molecule binding (GO:0036094), ion binding (GO:0043167), drug binding (GO:0008144) and cofactor binding (GO:0048037).

**Table 4:** Gene ontology of eight *Bemisia tabaci s. l.* populations

		***B. tabaci* SSA1-SG1-Ng**	***B. tabaci* SSA1-SG1-Ug**	***B. tabaci* SSA2-Ng**	***B. tabaci* SSA3-Ng**	***B. tabaci* Uganda-1**	***B. tabaci* Asia II-5**	***B. argentifolii***	***B. tabaci s.s***
**Genes**	**Total PCG**	13,661	12,710	12,928	13,463	12,749	12,749	12,077	15,786
	**PCG with GO:term**	8,996	8,677	8,497	8,823	8,032	8,407	8,290	10,793
**GO:Term**	**Biological**	5,447	5,229	5,131	5,335	4,826	5,126	5,041	6,707
	**Cellular**	2,731	2,587	2,546	2,666	2,494	2,626	2,572	3,281
	**Function**	7,787	7,598	7,309	7,544	6,786	7,287	7,236	9,335

Analysis of GO terms for eight *B. tabaci s.l.* populations, performed with WEGO (version 2.0; https://wego.genomics.cn/), where the protein-coding genes (PCGs) were annotated as part of this study. Gene ontology terms were provided only for canonical PCGs and provided as input in “WEGO native format” (Gene_ID; GO:_,GO:_,..). GO terms were assigned via InterProScan (v -5.40–77; http://www.ebi.ac.uk/interpro/). Summary of results for PCGs with identified GO terms for ontological categories: cellular compartment, biological process and molecular function. See Fig. 2 for graphical representation of enriched GO:Terms across the *B. tabaci s.l.* populations

#### Transposable elements

**Fig. 3:**
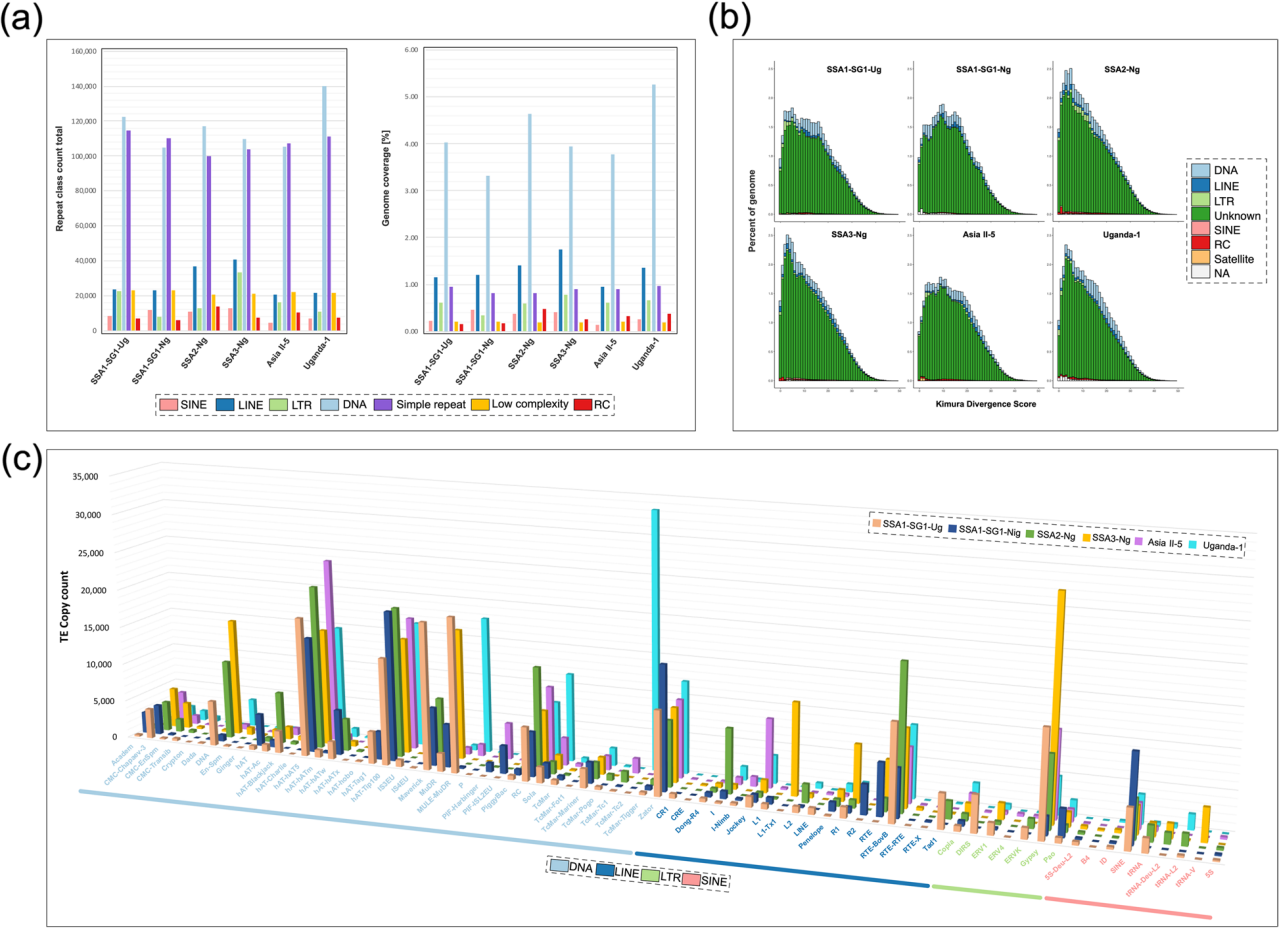
Genomic transposable element content in *Bemisia tabaci s.l.* genome assemblies. **a** Summary of major TE classes highlighting copy count (#) and genome repeat coverage (%). **b** Stacked bar-chart of Kimura sequence divergences of TE classes, expressed as a function of percentage of each genome; Y-axis: Genome percent coverage (%); X-axis: Kimura divergence score. **c** 3D-Bar graph showing TE copy count of repeat classes: DNA, LINE, LTR, SINE

Transposable elements (TE) are an important source of novel genomic variation and contributor not only to genome structural variation, but they can also influence changes to gene regulation [47–49]. TE content analysis of the six new *B. tabaci s.l.* genomes showed both shared species-specific differences, including repeat copy number counts and total genomic coverage (i.e., total genomic percent coverage) variability across TE families. The average genome repeat coverage, summed across all repeat classes of ~ 42% (268.3 Mb), ranged between 38.27% and 46.74% (see Fig. 3a, Table 5). Genomic TE coverage estimates for both SSA1-SG1 *B. tabaci s.l.* genomes were similar to that reported for the *B. tabaci* ‘SSA-ECA’ partial genome at 36.80–39.17% [29, 50]. Levels of TE coverage in the genomes of *B. argentifolii* (45%; 276.9 Mb) and *B. tabaci s.s.* (40.30%; 265.0 Mb) were intermediate; greater than both SSA1-SG1 genomes and Asia II-5, but less than SSA2-Ng (46.74%), SSA3-Ng (46.25%) and Uganda-1 (43.90%). Apart from SSA1-SG1-Ug, all other new *B. tabaci s.l.* genomes exhibited higher overall TE coverage than the closely related pea aphid (*Acyrthosiphon pisum;* 38%), in agreement with recent reports [50]. In contrast, we observed considerably lower genomics TE coverage across *B. tabaci s.l.* genomes, in comparison to the greenhouse whitefly *T. vaporariorum* (56.60%) [43].

**Table 5:** *Bemisia tabaci s.l.* transposable element summary statistics

*B. tabaci s.l.* genome	TE Statistic	Transposable element class											Genomic GC	Total Repeat GC	GC diff	Genome masked
		**SINE**	**LINE**	**LTR**	**DNA**	**Rolling-circles**	**Small RNA**	**Satellite**	**Simple repeat**	**Low complexity**	**Unclassified**	**Combined Total**				
***B. tabaci* SSA1-SG1-Ug** *(657.7 Mb)*	**Copy count**	8,221	23,676	22,606	122,504	7,048	2,034	1,873	114,820	23,003	1,032,414	1,358,199	-	-	-	-
	**Span (bp)**	1,504,504	7,586,278	4,065,482	26,608,725	1,075,915	183,698	473,301	6,220,683	1,351,002	199,426,376	248,495,964	-	-	-	-
	**Coverage (%)**	0.23	1.15	0.62	4.05	0.16	0.03	0.07	0.95	0.21	30.32	37.78	39.6	40.44	0.84	37.78
***B. tabaci* SSA1-SG1-Ng** ***(****656.2 Mb)*	**Copy count**	11,805	22,927	7,821	104,781	5,919	302	1,652	110,078	22,867	1,100,798	1,388,950	-	-	-	-
	**Span (bp)**	3,003,411	7,895,164	2,209,704	21,839,192	1,104,760	143,941	439,305	5,360,484	1,321,503	213,765,783	257,083,247	-	-	-	-
	**Coverage (%)**	0.46	1.20	0.34	3.33	0.17	0.02	0.07	0.82	0.20	32.57	39.17	39.59	40.67	1.08	39.17
***B. tabaci* SSA2-Ng ***(625.3 Mb)*	**Copy count**	10,786	36,764	12,933	117,049	1,3847	2,060	2,247	100,112	20,789	1,093,821	1,410,408	-	-	-	-
	**Span (bp)**	2,396,458	8,850,008	3,715,322	29,063,758	2,942,314	300,128	577,628	5,053,855	1,212,022	238,132,975	292,244,468	-	-	-	-
	**Coverage (%)**	0.38	1.42	0.59	4.65	0.47	0.05	0.09	0.81	0.19	38.08	46.74	39.63	41.31	1.68	46.74
***B. tabaci* SSA3-Ng ***(648.3 Mb)*	**Copy count**	12,665	40,819	33,578	109,579	7,615	2,691	1,983	103,648	21,228	1,116,514	1,450,320	-	-	-	-
	**Span (bp)**	2,660,271	11,352,651	5,024,618	25,645,693	1,643,239	683,945	517,288	5,783,986	1,252,960	245,293,574	299,858,225	-	-	-	-
	**Coverage (%)**	0.41	1.75	0.78	3.96	0.25	0.11	0.08	0.89	0.19	37.84	46.25	39.61	41.09	1.48	46.25
***B. tabaci* Asia II-5 ***(616.1 Mb)*	**Copy count**	4,312	20,780	16,212	105,077	10,283	822	1,980	107,152	22,167	1,004,819	129,3604	-	-	-	-
	**Span (bp)**	881,080	5,852,771	3,803,415	23,317,570	1,984,802	225,648	863,091	5,574,468	1,248,592	192,014,952	235,766,389	-	-	-	-
	**Coverage (%)**	0.14	0.95	0.62	3.79	0.32	0.04	0.14	0.91	0.20	31.17	38.27	39.63	40.77	1.14	38.27
***B. tabaci* Uganda-1** *(630.4 Mb)*	**Copy count**	6,901	21,781	10,742	140,247	7,603	1,674	438	111,149	21,539	98,8850	1,310,924	-	-	-	-
	**Span (bp)**	1,643,597	8,573,982	4,231,613	33,332,306	2,414,279	661,559	199,819	6,088,730	1,242,222	218,378,685	276,766,792	-	-	-	-
	**Coverage (%)**	0.26	1.36	0.67	5.29	0.38	0.10	0.03	0.97	0.20	34.64	43.90	39.5	41.27	1.77	43.90

Transposable element (TE) summary for the new *B. tabaci s.l*. genomes. TEs are summarized by their class

Columns left to right represent: complete TE copies (n); length span (bp) and total genomic coverage (%)

DNA TEs are part of class II type TEs, which mediate their movements via a DNA ‘cut and paste’ mechanism [47]. The majority of repeats identified in this study are DNA type transposons. The most widespread was DNA-hAT5, with *c.* 18,164 copies across all six genomes. One of the most striking examples of a species-specific DNA type TE expansion was ‘Tc-Mar-Tc2’ in Uganda-1 (*n* = 33,335). It is unknown why this is so prevalent in Uganda-1, but it may be related to Uganda-1’s phylogenetic position as one of the earliest branching *B. tabaci s.l.* species to originate in sub-Saharan Africa; a region considered to be the geographical origin of the *B. tabaci* group of species [7]. Estimates of DNA type TEs in the new genomes were noticeably higher than previous estimates of genomic DNA TE content in *B. argentifolii* (1.53%), yet more similar to *T. vaporariorum* (3.40%) [43]. Notably, the TE results presented here, when compared to a recent reanalysis of TEs in published *B. tabaci s.l.* genomes, showed that DNA superfamily diversity in *B. tabaci s.l.* is likely richer when methods designed at identifying hitherto uncharacterised TE repeats are applied, see [50].

LINE type retrotransposons (class I type transposons) transpose via an RNA intermediate, in which their transcription and transposition are facilitated via reverse transcriptase (RT) [47]. LINE TEs were the second most abundant repeat type identified in these new *B. tabaci s.l.* genomes, having an average genomic coverage of 1.30% and between 10 to 12 superfamilies each. Despite LINE family copy count differences between SSA2-Ng and SSA3-Ng, both populations had uniquely higher total LINE TEs with 36.7 k and 40.8 k copies, respectively, compared to a maximum of 23.6 k copies in SSA1-SG1-Ug. Although not a conclusive apomorphic trait; total LINE copy number of between 14.4 K and 18.5 K is a distinguishing feature of SSA2-Ng and SSA3-Ng populations when compared to all other *B. tabaci s.l.* and lends support for their designation as the same, single, biological species *B. tabaci* SSA2 ∪ SSA3 (see Table 1, Additional file: 1 Table S5). Further detailed analysis is warranted to fully explore the TE diversity, abundance, and divergence between these genomes. Total LINE genomic coverage across newly reported *B. tabaci s.l.* genomes were similar to those reported for *T. vaporariorum* (1.07%) and *B. argentifolii* (0.96%). Our results are supported by Sicat et al*.* [50], which showed a higher LINE coverage of 0.94% for *B. tabaci* ‘SSA-ECA’ genome than was initially reported (0.44%) [32].

Long terminal repeats (LTRs) are a group of autonomous retrotransposons similar to LINEs in that they are also mediated via a RT mechanism to “copy and paste” to new genomic loci [47]. Class I type LTR retroelements were the third most abundant TE type and displayed a slightly more uniform distribution but represented only ~ 0.6% of total genome coverage. The most abundant LTR family was ‘Gypsy’, with *c*. 11,546 copies. Instances of population specific LTR TE families were also observed, including ‘DIRS’ and ‘ERV4’ located in SSA1-SG1-Ug and Asia II-5, respectively (see Fig. 3c). Differentiated from LINEs and LTR TEs, which encode their own RT enzymes, SINE TEs rely on hitch-hiking copies of itself via the transcriptional machinery of autonomous class I TEs. SINE TEs had the smallest overall contribution to each of the *B. tabaci s.l*. genomes, the most abundant family was ‘SINE’ with between 3 to 5 k copies per genome and the SINE family ‘tRNA-V’ was uniquely identified in SSA3-Ng. Overall, we found broad similarities to previous estimates of LTRs and SINE TEs in *T. vaporariorum*, *B. argentifolii*, *B. tabaci s.s.* and *B. tabaci* ‘SSA-ECA’ [50]. See Table 5 and Additional file 1: Table S5 for a full breakdown of *B. tabaci s.l.* TE family copy diversity.

Kimura-distance-based divergence analysis [48] was used to estimate the relative age of TE types for the six new *B. tabaci s.l.* genomes (Fig. 3b). ‘Kimura scores’ are based on relative distance from the reconstructed repeat consensus sequence, so lower values represent a “younger” or more recently acquired sequence. Peaks along the X-axis shown in Fig. 3b indicate increased acquisition of younger TE*.* The pattern outlined above is largely conserved, whereby the age distribution and overall genomic TE coverage followed a similar distribution across all six genomes, culminating in a Kimura divergence score of ~ 40. Of the characterized TEs, pronounced peaks in DNA TEs across all six genomes were observed and represents the largest overall total contributor and continued temporal TE activity identified in this study. *Bemisia tabaci* SSA2 ∪ SSA3 (both SSA2-Ng and SSA3-Ng genomes) showed subtle, yet clearly observable, patterns of more recent acquisition of DNA and LINE type TEs. Peak LTR TE activity for SSA1-SG1-Ug was evident between Kimura score 1 to 6, and for SSA2-Ng between Kimura score 8 to 14. The pattern of LTR activity observed in SSA1-SG1-Ug and SSA2-Ng is not seen in the other *B. tabaci s.l.* genomes, though Uganda-1 does also show signs of increased LTR TE activity between Kimura score 1 to 3.

To gain a greater evolutionary context regarding *Bemisia* TE evolution, we examined *B. tabaci s.l.* TEs with reference to the Arthropoda TE complement first presented in Petersen *et. al.* [49]. Notably, the new *B. tabaci s.l.* genomes exhibit some of the highest proportion of hitherto uncharacterized TE diversity (~ 85%), second only to species of mayfly, such as *Ephemera danica* or *Eurytemora affinis* (at + 93% uncharacterized) and comparable to the more closely related species, *F. occidentalis* (~ 87%) [49]. The apparent level of uncharacterized TE in our *B. tabaci s.l.* genomes have recently been improved whereby DNA and LINE type TE superfamilies were shown to be present at a higher total genomic coverage compared to our analysis [50]. Here, between 45 and 55 TE superfamilies were identified across all six *B. tabaci s.l.* genomes; but it is likely that it is closer to ~ 80 superfamilies when uncharacterized TEs are identified through clustering and in-depth characterization methods. Importantly, TE diversity described here was close to that reported for pea aphid, *A. pisum*, another phloem-feeding hemipteran [49]. Despite additional analysis being required to fully characterize and assign family level affinity across all TE examined; our results nonetheless emphasize *B. tabaci s.l.* genomes have some of the highest proportion of total genomic TE coverage for all major Arthropoda clades examined to date.

#### Whole genome comparative genomics

**Fig. 4:**
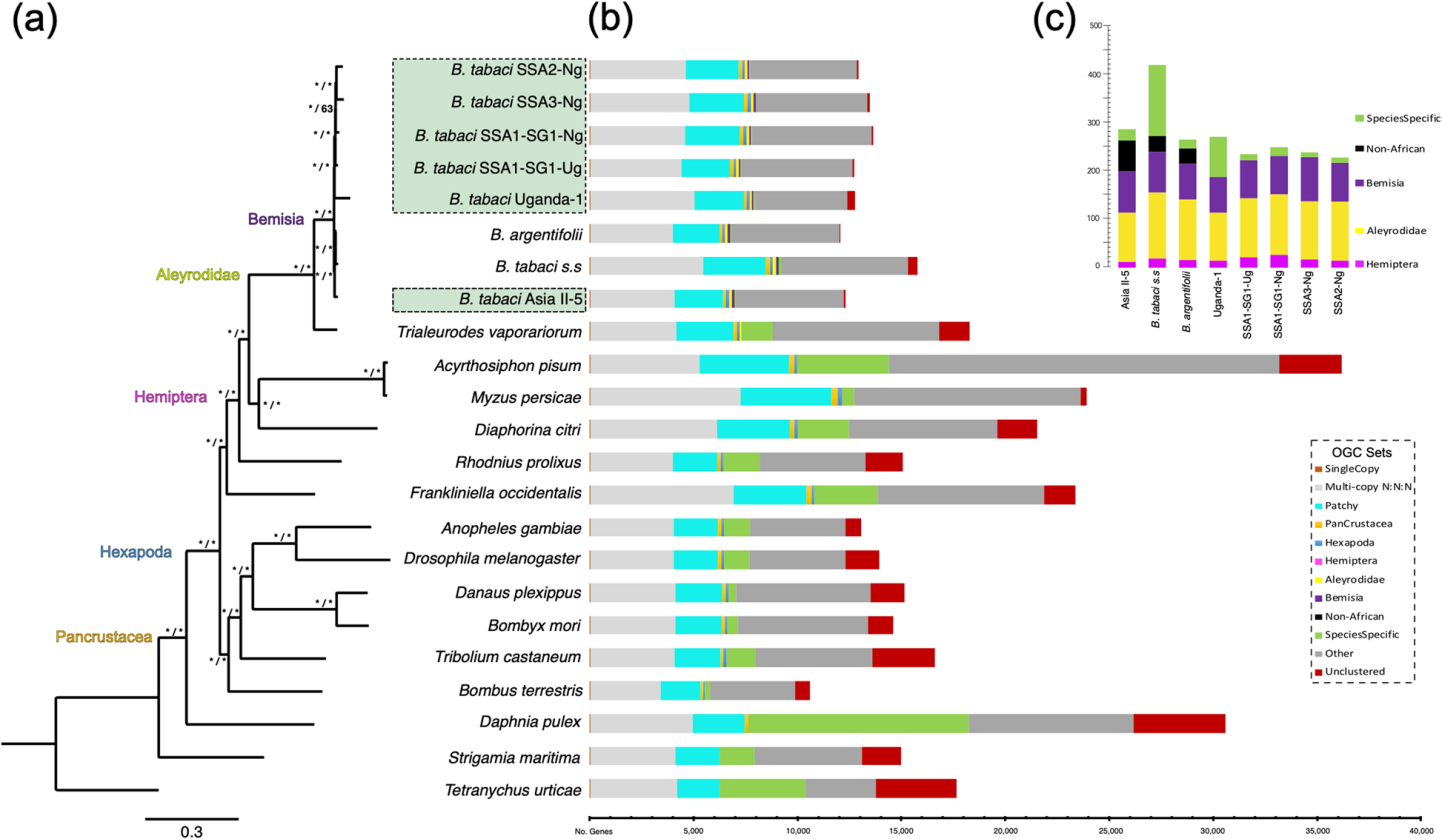
A genome wide species level phylogeny with clade specific orthologs. Whole genome comparative analysis computed with Orthofinder. Publicly available whitefly species were *B. argentifolii*, *B. tabaci s.s.* and *T. vaporariorum* (“Greenhouse whitefly”). Phylogenetic relationships estimated with RAxML (maximum likelihood) and MrBayes (Bayesian posterior probability) on a concatenated matrix of protein sequences of 23 species covering 655 OGCs (131,953 amino acids). **a** Species-level phylogeny with associated node support values (*/*) ⟹ Bayesian PP / BS (bootstrap replicates *n* = 100); under the best-fitting substitution model LG + G + F + I. **b** Ortholog set delineation depicted with respect to major Arthropoda clades (Pancrustacea, Hexapoda, Hemiptera and Aleyrodidae); ‘Multi-copy N:N:N’ ortholog sets contain ≥ 1 gene across all species; ‘Patchy’: missing a single species representative. The six new *B. tabaci s.l.* populations are highlighted in green dashed boxes. **c** OGC clade sets with relatively low gene counts expanded for clarity

Comparative genomics of *B. tabaci s.l.* was performed via OrthoFinder (v 2.4.0) analysis [51, 52] and included twenty-three Hexapoda genomes, with a primary focus on sampling more closely related Hemiptera taxa (Fig. 4, Table 6). Non-whitefly Hemiptera included: *A. pisum* (pea aphid)*, Diaphorina citri* (Asian citrus psyllid)*, Myzus persicae* (green peach aphid) and *Rhodnius prolixus* (kissing bug). To improve overall resolution of the ingroup *B. tabaci s.l.,* the reannotated genomes for *B. argentifolii* and *B. tabaci s.s.* produced for this study were included. The non-*Bemisia* Aleyrodidae greenhouse whitefly *T. vaporariorum* [43] was selected as an outgroup, being the only other whitefly species with a completely sequenced genome at the time of analysis. Complete results, listed ingroup taxa and data obtained from the comparative analysis with OrthoFinder, including OGCs, sequences and preliminary gene trees can be obtained from the data repository ‘Figshare’ [53]; see ‘Materials and methods’.

**Table 6:** Comparative genomics of *Bemisia tabaci s.l*. and related insects with OrthoFinder

Species	Common name	Annotation source	INSDC^a^ assembly accession	Input genes	Unassigned genes	Genes in OGCs^b^	Genes in OGCs (%)	Number of OGCs incl. species	OGCs incl. species (%)	Speciesspecific OGCs	Genes in speciesspecific OGCs	Genes in speciesspecific OGCs (%)
*Acyrthosiphon pisum*	Pea aphid	Ensembl Metazoa (rel. 100)	GCA_000142985	36,195	3,031	33,164	91.6	10,228	46.0	891	4,410	12.2
*Anopheles gambiae*	African malaria mosquito	Ensembl Metazoa (rel. 100)	GCA_000005575	13,057	756	12,301	94.2	7,792	35.1	219	1,229	9.4
*Bombus terrestris*	Buff-tailed bumblebee	Ensembl Metazoa (rel. 100)	GCA_000214255	10,581	674	9,907	93.6	7,697	34.6	62	276	2.6
*Bombyx mori*	Domestic silk moth	Ensembl Metazoa (rel. 100)	GCA_000151625	14,623	1,249	13,374	91.5	8,677	39.0	112	498	3.4
*B. tabaci* SSA1-SG1-Ng	African cassava whitefly	Ensembl Metazoa (rel. 103)	GCA_902825415	13,661	104	13,557	99.2	7,822	35.2	9	19	0.1
*B. tabaci* SSA1-SG1-Ug	African cassava whitefly	Ensembl Metazoa (rel. 103)	GCA_902825425	12,710	68	12,642	99.5	7,371	33.2	3	13	0.1
*B. tabaci* SSA2-Ng	African cassava whitefly	Ensembl Metazoa (rel. 103)	GCA_903994125	12,928	70	12,858	99.5	7,682	34.6	5	11	0.1
*B. tabaci* SSA3-Ng	African cassava whitefly	Ensembl Metazoa (rel. 103)	GCA_903994115	13,463	119	13,344	99.1	7,727	34.8	5	10	0.1
*B. tabaci* Asia II-5	Indian cassava whitefly	Ensembl Metazoa (rel. 103)	GCA_903994105	12,289	62	12,227	99.5	7,687	34.6	6	24	0.2
*B. tabaci* Uganda-1	Sweet-potato whitefly	Ensembl Metazoa (rel. 103)	GCA_903994095	12,749	347	12,402	97.3	6,853	30.8	40	85	0.7
*B. argentifolii*	Silverleaf whitefly	Ensembl Metazoa (unreleased)	GCA_001854935	12,077	65	12,012	99.5	7,950	35.8	6	19	0.2
*B. tabaci s.s*	Tobacco whitefly	Ensembl Metazoa (unreleased)	GCA_003994315	15,784	485	15,299	96.9	7,873	35.4	68	151	1.0
*Danaus plexippus*	Monarch butterfly	Ensembl Metazoa (rel. 100)	GCA_000235995	15,128	1,597	13,531	89.4	9,088	40.9	121	369	2.4
*Daphnia pulex*	Common water flea	Ensembl Metazoa (rel. 100)	GCA_000187875	30,590	4,437	26,153	85.5	9,034	40.6	1,601	10,616	34.7
*Diaphorina citri*	Asian citrus psyllid	NCBI-RefSeq (06–2020)	GCF_000475195	21,517	1,892	19,625	91.2	8,698	39.1	919	2,479	11.5
*Drosophila melanogaster*	Fruit fly	Ensembl Metazoa (rel. 100)	GCA_000001215	13,947	1,630	12,317	88.3	7,819	35.2	324	1,235	8.9
*Frankliniella occidentalis*	Western flower thrips	NCBI-RefSeq (06–2020)	GCF_000697945	23,356	1,472	21,884	93.7	9,021	40.6	743	3,012	12.9
*Myzus persicae*	Green peach aphid	NCBI-RefSeq (06–2020)	GCF_001856785	23,910	275	23,635	98.8	8,975	40.4	172	553	2.3
*Rhodnius prolixus*	Kissing bug	Ensembl Metazoa (rel. 100)	GCA_000181055	15,061	1,803	13,258	88.0	7,733	34.8	310	1,739	11.5
*Strigamia maritima*	Centipede	Ensembl Metazoa (rel. 100)	GCA_000239455	14,992	1,902	13,090	87.3	7,245	32.6	369	1,684	11.2
*Tetranychus urticae*	Two-spotted spider mite	Ensembl Metazoa (rel. 100)	GCA_000239435	17,671	3,892	13,779	78.0	6,443	29.0	660	4,110	23.3
*Trialeurodes vaporariorum*	Greenhouse whitefly	WhiteflyDB (06–2020)	GCA_011764245	18,275	1,467	16,808	92.0	8,277	37.2	276	1,509	8.3
*Tribolium castaneum*	Red flower beetle	Ensembl Metazoa (rel. 100)	GCA_000002335	16,590	3,001	13,589	81.9	8,294	37.3	284	1,371	8.3

^a^ *INSDC *International Nucleotide Sequence Database Collaboration

^b^ *OGCs* Orthologous gene clusters

A comparison of 23 arthropod taxa including the six new *B. tabaci s.l.* new genomes. Analysis was performed via Orthofinder (v2.4.0) [51], by providing canonical protein-coding sequences as input. The Orthofinder pipeline implemented both MSA and phylogenetic gene tree reconstruction with default settings. All *B. tabaci s.l.* gene sets were generated via the Ensembl gene annotation pipeline. Newly generated *B. tabaci s.l.* were first released via Ensembl Metazoa (release e103) (https://metazoa.ensembl.org). For previously published *B. argentifolii* and *B. tabaci s.s.* re-annotated datasets see Additional File 5. The protein-coding gene (PCG) set of *T. vaporariorum* was obtained from WhiteflyDB (http://www.whiteflygenomics.org). Remaining PCG sets were obtained directly from Ensembl Metazoa (release e100), using the Ensembl Perl API or alternatively downloaded from NCBI-RefSeq (June—2020)

Of the 391,154 input genes that received orthology assignment, 360,756 (92.2%) genes were successfully clustered into 22,225 orthologous gene clusters (OGCs). A total of 2,297 OGCs (10.3%) contained all twenty-three species while 7,205 (32.4%) OGCs were species-specific gene clusters representing 9.1% of all genes analyzed. 2,404 OGCs (10.81% of all OGCs) included all eight *B. tabaci s.l.* species considered, while on average 7,620 OGCs contained one or more representative genes. We recovered 142 (0.64%) OGCs as *B. tabaci s.l.* species specific. Excluding all *B. tabaci s.l.*, the average species specific OGC count rose to 470 (2.1%). An explanation for the discrepancy in species specific OGC counts and evidence of concordance of annotation could stem from our application of a uniform methodology across *B. tabaci s.l.*, in contrast to the remaining taxa obtained from a range of community annotations that potentially could have overestimated genes or included poor-quality models.

The recovery of taxonomically clustered OGCs within: (i) the Hemiptera (excluding whitefly), (ii) whitefly and iii) *B. tabaci s.l.*, showed largely similar numbers of OGCs (Fig. 4b, c, Additional file 1: Table S6). Hemiptera had an average count of seventeen OGCs per species, with a maximum of twenty-eight observed in *B. tabaci* SSA1-SG1-Ng and a minimum of two in *R. prolixus*. Overall, within the Aleyrodidae, *B. tabaci s.l.* species recovered an average of 123 OGCs per species, while *T. vaporariorum* had a total of 71 OGCs. The genome of *B. tabaci s.s.* uniquely exhibited many more species-specific OGCs (*n* = 151), compared to an average of 41.5 amongst the remaining *B. tabaci s.l*. Forty-seven OGCs contained unique, single-sequence, one-to-one, single-copy orthologs. This increased to 655 OGCs, when the minimum percentage of ingroup taxa represented by a single sequence ortholog was reduced to 78.3%.

### Integrative systematics: a consilience of evidence

#### Phylogenomic analysis of *B. tabaci s.l.* and other insects

Phylogenomic analyses on an alignment of 655 OGCs or 131,953 amino acids (average % missing data = 10.34%) were conducted using the best fitting evolutionary model (LG + Γ + I + F) under both maximum likelihood (ML) and Bayesian inference (BI) methods using RAxML and Mr. Bayes, respectively. Only amino acid sequences of the longest canonical protein-coding transcript were considered. Results from both ML and BI phylogenetic analyses recovered near universal support of the exact same topology, providing high support for monophyletic whitefly (*Aleyrodidae)* and monophyletic *B. tabaci s.l.* (Fig. 4a). Overall topological support recovered from ML and BI analyses was similar with linear log-likelihood values of *lnl*-2.359003 and *lnl*-2.360958, respectively. All African *B. tabaci s.l.* species clustered into a single clade (“Africa-only”: Uganda-1, SSA1-SG1-Ug, SSA1-SG1-Ng, SSA2-Ng, SSA3-Ng) sister to a clade of all non-African whitefly (*B. argentifolii*, *B. tabaci s.s.* and Asia II-5). Of the three non-African whiteflies, *B. argentifolii* and *B. tabaci s.s.* clustered as each other's closest relative, to the exclusion of Asia II-5.

Full posterior probability (PP) and 100% of all bootstrap (BS) replicates (*n* = 100) supported the placement of the non-cassava feeding species Uganda-1 as the earliest branching member of “Africa-only” whitefly clade. The sister group to Uganda-1 was *B. tabaci* SSA1-SG1-Ug, which was placed in a paraphyletic relationship with SSA1-SG1-Ng. Topological support within this clade only differed regarding the placement of SSA1-SG1-Ng; whose position in the phylogeny was the least stable, highlighted by a fall in BS support (63%).

Full support was recovered across both ML and BI analyses for monophyletic Hemiptera, in which whitefly were sister grouped to a clade of aphids (*A. pisum* + *M. persicae*) and *D. citri*, to the exclusion of *R. prolixus* which was positioned as the earliest branching member of all Hemiptera taxa. Hexapoda was recovered as monophyletic, with full support (ML, BI) of Pancrustacea including Hexapoda sister to *D. pulex*. Remaining taxa were positioned at the base of Arthropoda, falling in line with the current understanding of major orders and subphyla within Arthropoda [54, 55].

#### Biological species inferred by reciprocal cross-mating

We determined the reproductive compatibility between selected populations of *B. tabaci s.l.* using single-pair and group-pairing reciprocal cross-mating tests. *Bemisia tabaci s.l.* reproduces by arrhenotoky, whereby unmated females produce only male progeny. In contrast, mated females produce both male and female progeny, whereby the female progeny develop sexually from fertilized eggs [56]. We used this phenomenon, therefore, to assess mating success and reproductive compatibility, by the presence or absence of female offspring. Cross-mating tests involved pairing 3:1 and 15:5 males to females from different populations, including SSA1-SG1-Ng, SSA1-SG1-Ug, SSA2-Ng, SSA2-Ug, and SSA3-Ng (Additional file 1: Tables S7, S8).

**Fig. 5:**
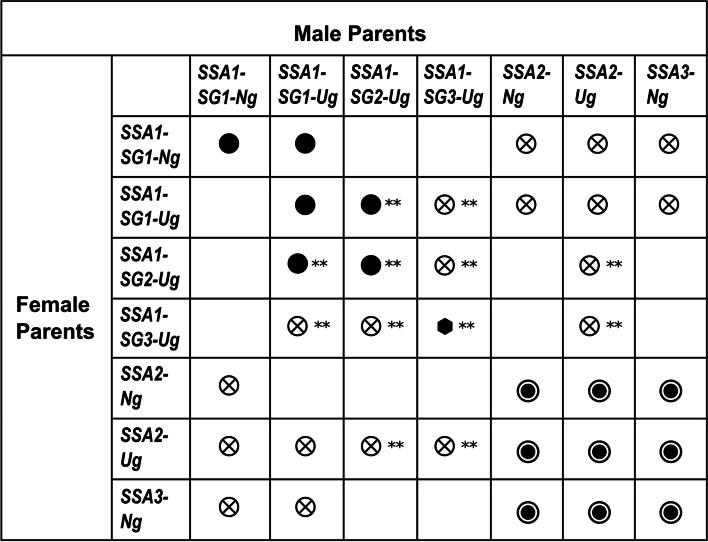
Reproductive compatibility of eight *B. tabaci s.l.* populations collected in Uganda and Nigeria. Male parents (top row) and female parents (left column). Symbols represent the degree of reproductive compatibility. The black circle (⚫) represents complete reproductive compatibility between members of the SSA1-SG1 ∪ SG2, the fisheye circle (◉) represents complete reproductive compatibility between members of the SSA2 ∪ SSA3 species, the hexagon (⬢) represents complete reproductive compatibility observed in the SSA1-SG3 population, while the circled-cross ( ⊗) represents complete reproductive incompatibility with no female progeny production in F1 generation. The mating-crosses denoted by double asterisks (**) were carried out by Mugerwa et al*.* [8]

Reproductive incompatibility was observed between sympatric populations that differed by more than 7% in their partial mtCO1 sequences, i.e. SSA1-SG1-Ng x SSA3-Ng, SSA1-SG1-Ng x SSA2-Ng. Cross-mating of most allopatric populations did not produce any female progeny, indicating complete reproductive incompatibility. However, SSA1-SG1-Ug x SSA1-SG1-Ng and SSA2-Ng x SSA3-Ng crosses demonstrated complete reproductive compatibility, producing female progeny in both single-pair and group-mating crosses. Importantly, the partial mtCO1 of SSA2-Ng and SSA3-Ng differs by over 6.1%, exceeding the proposed 3.5% species threshold by 2.6% [9, 26]. In addition, control backcrosses showed that the F_1_ progeny were fertile (Fig. 5, Additional file 1: Tables S7, S8). Similar observations were reported for two putative species Asia II-9 and Asia II-3, with mtCO1 divergence of > 4.5%, where reproductive compatibility was shown to be complete in one direction, and partial in the other [57].

Overall, these results highlight the need for additional genetic markers to enable accurate differentiation of the biological species within *B. tabaci s.l.,* e.g. the concatenated nuclear-gene sequences in building the species tree [58]. Although SSA2 and SSA3 are evidently the same biological species, which we now call, *B. tabaci* SSA2 ∪ SSA3 (Table 1), the SSA3 population has mostly been recorded from the rainforest ecological zones of West Africa, while SSA2-Ug populations are mostly present in the drier and higher-temperature latitudes of the Sahel [59]. It is probable, therefore, that the observed genetic differences reflect ecological adaptations to the different ecological zones they occupy.

#### Whitefly systematics using mitochondrial genomes

Phylogenetic analyses were performed using concatenated 11 mitochondrial PCGs on 28 hemipteran species and one Coleoptera as outgroup (Additional file 1: Table S9). The higher rate of mutation of mitochondrial sequences compared to nuclear sequences meant that we had to restrict the phylogenetic analyses to only Hemipteran, rather than including Hexapoda and Pancrustacea as done for the genome-wide phylogenetic analysis [60]. The nucleotide sequences of the 11 PCGs were better suited for inferring phylogenetic relationships within the Hemiptera compared to the isolated mtCO1 gene (data not shown). Within the Aleyrodidae, support values were also better for the concatenated mitochondrial PCGs (Fig. S8). Phylogenetic reconstruction shows that *B. tabaci s.l.* populations from Sub-Saharan Africa occupied a monophyletic clade with Uganda-1 positioned as the earliest branching member.

Although the partial mtCO1 marker provides a good initial framework for identifying putative biological species within *B. tabaci s.l.*, analysis of mtCO1 alone has limitations. There is a need, therefore, to continue the integrative approach [6] for identifying biological species within the group which involves combining evidence from biological and molecular datasets. An integrative approach, as applied herein, supports that the seven African cassava populations of *B. tabaci s.l.* represented in Fig. S8, can be reduced to three biological species. Hence, the SSA1-SG1 Nigeria, SSA1-SG1 Uganda and SSA1-SG2 Kayingo populations were grouped as “*B. tabaci* SSA1-SG1 ∪ SG2” and SSA2 Kiboga, SSA2 Nigeria and SSA3 Nigeria were grouped as “*B. tabaci* SSA2 ∪ SSA3” while SSA1-SG3 remains distinct from “*B. tabaci* SSA1-SG1 ∪ SG2” (Table 1, Fig. S8).

#### Endosymbiont *Portiera* co-cladogenesis and metabolic potential

*B. tabaci s.l.* contain endosymbiotic bacteria, including the primary obligate endosymbiont, ‘*Candidatus* Portiera aleyrodidarum’ (hereafter *Portiera*) and up to seven secondary, facultative endosymbionts; *Cardinium*, *Arsenophonus*, *Hamiltonella*, *Rickettsia*, *Wolbachia*, *Fritschea* and *Hemipteriphilus asiaticus* [61, 62]. Of these, *Portiera, Arsenophonus*, *Cardinium*, *Hamiltonella, Rickettsia*, and *Wolbachia* have been reported previously from *B. tabaci* SSA1 [62]. The population of *B. argentifolii* used to generate the draft genome, for example, contained *Portiera* and two secondary endosymbionts, *Hamiltonella* and *Rickettsia,* which had assembled genome sizes of 352 kb, 1.74 Mb and 1.38 Mb, respectively. For *P. aleyrodidarum,* 273 genes were predicted whose functions were essential for basic cellular processes and whitefly nutrition [32].

Strict vertical transmission over long evolutionary periods result in primary endosymbionts reflecting their host phylogeny (co-cladogenesis) [63]. Indeed, genomic and molecular dating analysis show that *Portiera* has been associated with whiteflies since their origin, more than 125 Mya [64–66]. We assembled the *Portiera* genomes of the six new *B. tabaci s.l.* populations to examine whitefly phylogenetic relationships. The genomic characteristics of the newly obtained *Portiera* were similar to those from *Portiera* associated with *B. tabaci s.s*., Asia II-3 and *B. argentifolii* (Additional file 1: Table S10). The number of frame-shifted genes was extremely high in some *Portiera*, however, especially those from *B. tabaci* Uganda-1 and SSA1-Ng. Frameshifts were generally caused by low-complexity DNA regions, mostly in repetitive ‘A’ rich polymer regions. Although the correct frame could be recovered due to the polymerase slippage [67], the negative correlation between assembly coverage and the number of frameshifted genes suggests this problem is more related to sequencing artifacts from the technology used (PacBio Sequel).

Despite known frameshift issues, *Portiera* genomes are a complementary source of information to study whitefly evolution. First, we compared the major functions encoded by the different *Portiera* genomes (Additional file 2: Fig. S9). If frame-shifted genes are considered as coding sequences, we found no differences in the information transfer and translation machinery, nor in the metabolic potential (energy, essential amino acids and co-factors), encoded by *Portiera* from *B. tabaci s.s*., *B. tabaci s.l.* and *B. argentifolii* (Additional file 2: Fig. S9). Differences in gene content (e.g., different pseudogenization events) are expected when comparing *Portiera* from different whitefly hosts [68]. Therefore, the maintenance of the same gene content suggests a close relationship among *Bemisia* harboring *Portiera* with the same encoding capabilities.

**Fig. 6:**
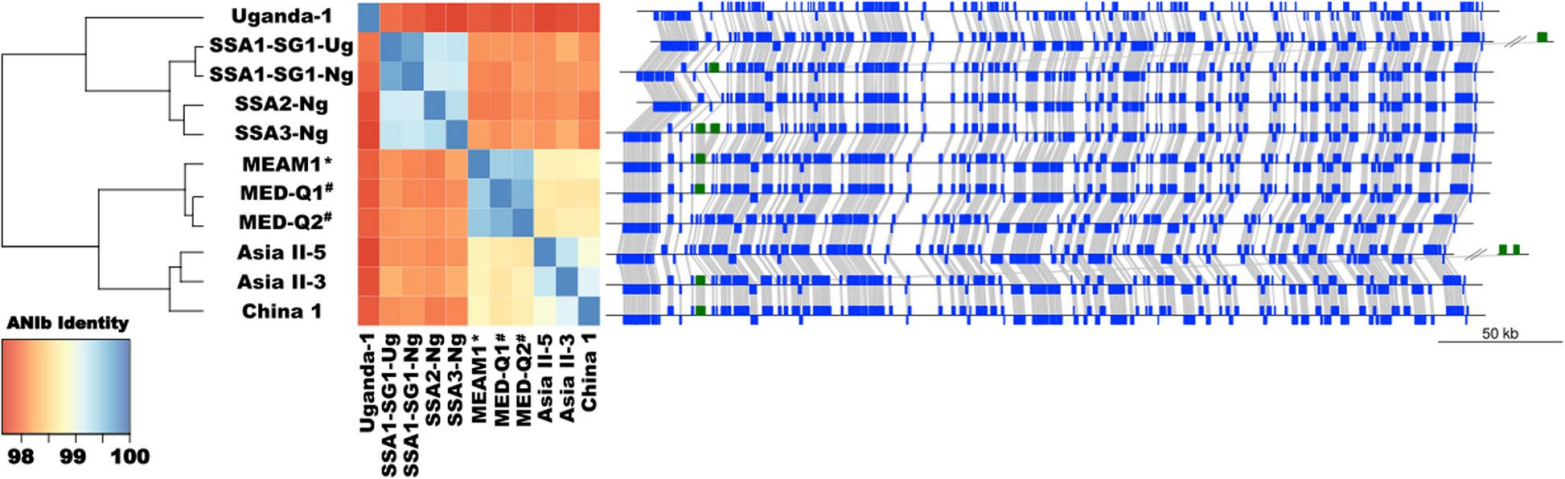
Average Nucleotide Identity and genomic synteny among *Candidatus* Portiera aleyrodidarum from different *Bemisia* hosts The cladogram on the left summarizes *Portiera* relationships based on their pairwise Average Nucleotide Identity values (heatmap, middle). On the right, genomic synteny conservation among *Portiera* strains based on 202 complete Coding Sequences (CDS) (blue) and the CDS presence in the variable region (green). *Portiera* genomes are represented linearly, the presence of a subcircular conformation of the variable region is represented at the end of the plot (separated by double backslashes). Blue boxes representing syntenic CDS in the direct strand (upwards) or in the complementary strand (downwards), genes from the variable region are denoted in green. Gray lines connect orthologous CDS

Second, we computed the average nucleotide identity (ANI) among *Portiera* genomes from *B. tabaci s.s.*, *B. tabaci s.l.* and *B. argentifolii* species. Their ANI values were above 96% (Fig. 6), suggesting that all of the new bacterial genomes are strains of the same *P. aleyrodidarum* [69]. A cluster dendrogram analysis (Fig. 6), however, grouped the genomes of *Portiera* from *B. tabaci* SSA2 and *B. tabaci* SSA3 in one cluster while those from *B. tabaci* SSA1-Ng and *B. tabaci* SSA1-Ug were recovered in a different one, showing within-species evolutionary changes. These data, therefore, add additional evidence for concluding that *B. tabaci* SSA2 and *B. tabaci* SSA3 are the same biological species (called here, *B. tabaci* SSA2 ∪ SSA3). Indeed, clustering analysis of ANI values recovered a similar topology as the ones obtained by nuclear and mitochondrial genes, (Fig. 4 and Fig. S8 respectively).

*Portiera* from whitefly species belonging to the Aleurolobini tribe, which includes the *Bemisia* genera, present different genome architectures [68]. Indeed, the genome architecture of *Portiera* is less conserved among Aleurolobini species that diverged longer ago, such as *Singhiella simplex*, which separated from the branch leading to *B. tabaci s.l. c.* 71.34 Mya [68]. Therefore, the maintenance of the genomic architecture (macro and micro-synteny) among *Portiera* from *B. tabaci s.s*., *B. tabaci s.l.*, and *B. argentifolii* suggests a recent divergence, in evolutionary terms, of their hosts (Fig. 6). The exception in genome order was mainly the region encoding for three genes (yidC, mnmE, and mnmG). This region is known to be present as an episome (sub-circular particles) or integrated into the chromosome. Also, the number of gene copies varies from zero (absent) to at least three copies [70]. This region was detected as an episome in *B. tabaci* SSA1-Ug (single copy) and Asia II-5 (two copies), integrated into the chromosome of *B. tabaci* SSA2 (two copies) and absent in *B. tabaci* SSA3 and Uganda 1 (Fig. 6). However, variations in this region are unrelated to the host species, since variation occurs even at the intrapopulation level [70].

For the six new *B. tabaci s.l.* genomes, only *Hamiltonella* reads were found for *B. tabaci* Uganda-1. We obtained 12 scaffolds, with a total size of 1,609,740 bp for the *Hamiltonella* genome. *Hamiltonella* and *Arsenophonus S-*endosymbionts supply their host with B vitamins, therefore, they are required for whitefly development [71, 72]. A possible explanation for the absence of S-endosymbionts sequences was the use of adult males for sequencing. In adult males, bacteriocytes degenerate with age, thus reducing the number of endosymbionts present, especially S-endosymbionts [73].

### Gene families associated with detoxification, sugar metabolism and cassava adaptation

Host-plant association studies suggest that *B. tabaci s.l.* is a group of more than 40 oligophagous species, with only a few possessing a truly broad host-plant range. RNA-Seq analyses have shown that *B. tabaci s.l.* have an ancestral, or converged, expression pattern of the detoxification “machinery” that is shared amongst species and that enables them to perform well on multiple common and novel hosts [74]. In a study of the genetic diversity of whitefly (*Bemisia* spp.) on crop and uncultivated plants in Uganda, the most prevalent whiteflies were *B. tabaci* MED-ASL (30.5% of samples), *B. tabaci* SSA1 (22.7%) and *B. tabaci* Uganda-1 (12.1%), which were also the most polyphagous occurring on 33, 40 and 25 different plant species, respectively. Although all three species exhibited a high level of polyphagy, only *B. tabaci* SSA1-SG1 ∪ SG2 and *B. tabaci* SSA2 ∪ SSA3 were present on cassava [31], suggesting clear differences in the abilities of these species to process cassava’s phytotoxins.

Known detoxification gene families, including cytochrome P450s, (UDP)-glucuronosyltransferases, glutathione transferases, ABC transporters and carboxylesterases were reported from the draft *B. argentifolii* genome [32]. Of these, the carboxylesterases and UDP-glucuronosyltransferases were expanded significantly, relative to most other insect genomes. Expansion of these six detoxification gene families in *B. tabaci s.l.* is probably central to their success [31] and has enabled insecticide resistances to evolve rapidly.

Here, we characterized detoxification gene families in *B. tabaci s.l.* to understand their potential role in adaptations to cassava, as well as to insecticides. Utilizing queries derived from published proteins of *B. argentifolii* [32], we conducted phylogenetic analysis of these targeted detoxification enzyme families in *B. tabaci s.l.* that operate in a three-phase process. Phase I includes mainly the activity of cytochrome P450 monooxygenase (P450s) and carboxylesterase enzymes (COEs), which reduce, hydrolyze, or oxidize a variety of endogenous toxic compounds and exogenous substances. Phase II includes enzymes mainly from the glutathione S-transferases (GSTs), uridine diphosphate (UDP)-glucuronosyltransferases (UGTs) and cytosolic sulfotransferases (SULTs) families. These enzymes catalyze the conjugation of glutathione, glucose or sulfonate group to the reactive site of the phase I products, thereby increasing their polarity and facilitating their excretion. Phase III includes mainly ATP-binding cassette transporters (ABCs) that export the conjugated products out of the cells [75, 76]. Most studies on the functionality of detoxification gene families in *B. tabaci s.l.* have focused so far on their involvement in host-plant adaptation [74, 77] and insecticide resistance [78].

**Fig. 7:**
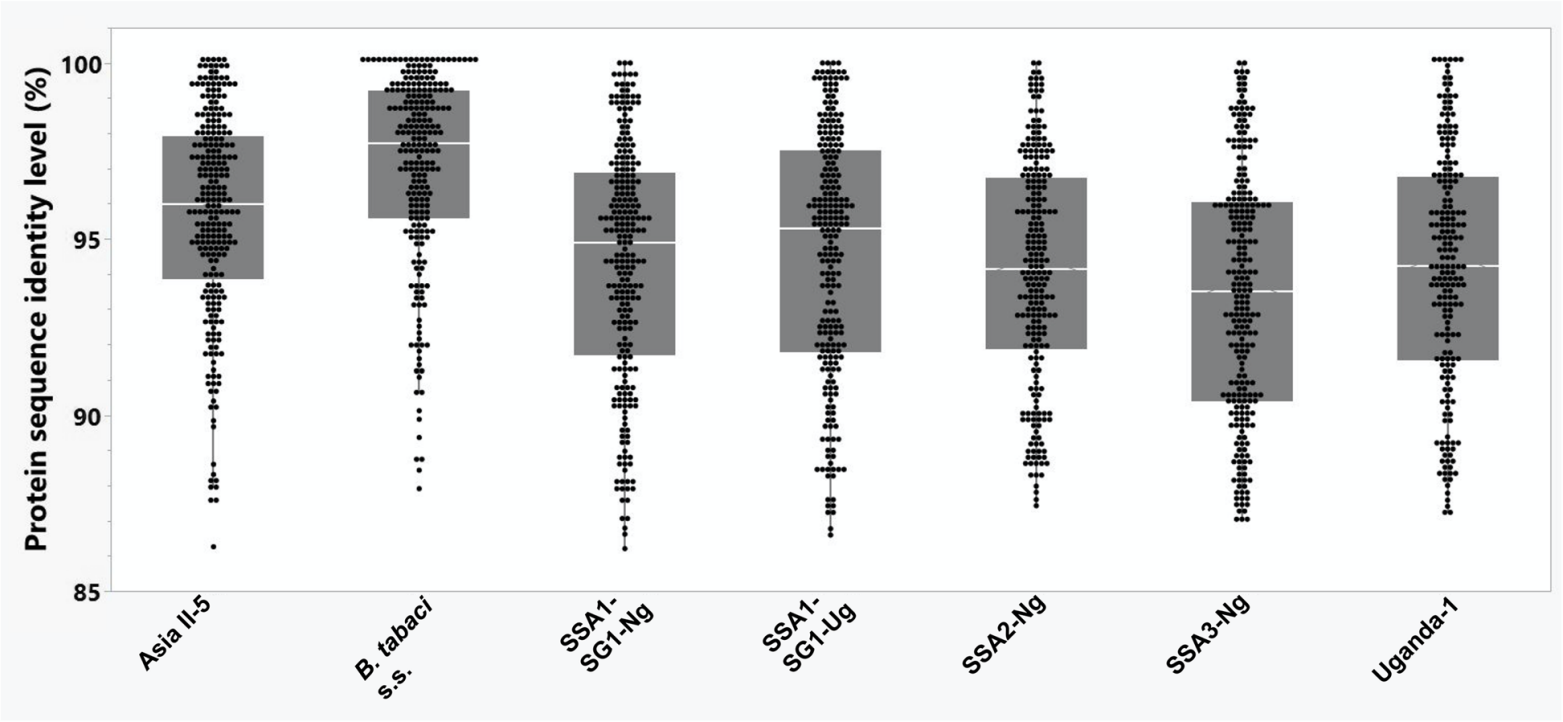
Protein identity across detoxification gene families. A box-plot representation of a curated set of detoxification proteins obtained from *B. argentifolii* and their putative orthologous protein (each represented by a single dot) in seven analyzed species of *Bemisia tabaci s.l*: Asia II-5, *B. tabaci* s.s., SSA1-SG1-Ng, SSA1-SG1-Ug, SSA2-Ng, SSA3-Ng and Uganda-1. A BLAST-combined with manual inspection approach used to check the identity of each protein. All alignments shown include putative orthologous proteins with at least 100 amino acids of the entire sequence aligned (cutoff >  = 85% PID). The number of proteins analyzed between species vary, as it was not always possible to recover a *B. argentifolii* orthologue in each of the seven analyzed *Bemisia* species

Overall, we detected variation in the number of detoxification genes in the five African species, ranging from 207 in SSA3-Ng to 164 in *B. tabaci* Uganda-1 (the genome of the reference *B. argentifolii* species harbors 268 detoxification genes). The identity level (of the proteins coded by the detoxification genes) between the African species and *B. argentifolii* was surprisingly high, ranging from 93.1% in the COE family of SSA2-Ng, to 97.2% in the ABC family of SSA1-SG1-Ug (Fig. 7, Additional file 1: Table S11). Even so, ~ 10% of the genes in each Species X Gene family combination showed an identity level of 90% or lower. For full details on the input sets of *B. argentifolii* detoxification gene family queries and a summary of PAV (Presence Absence Variation) across the seven *B. tabaci spp*. examined see Additional file 1: Table S12 and Table S13 respectively.

#### Carboxylesterase enzymes (COEs)

COEs catalyze the hydrolysis of an ester bond into the corresponding alcohol and carboxylic acid. Our analysis focused on enzymes previously proposed to display digestive or detoxification functions [79, 80]. In *B. tabaci s.l.*, these enzymes (α/β esterase) play a role in the detoxification of several important groups of insecticides, such as pyrethroids, organophosphates, and carbamates [81]. Testing the presence/absence of *B. argentifolii* orthologs in the three African grouped biological species (Additional file 1: Table S12, Table S13, Additional File 2: Fig. S10, indicated the absence of seven orthologs in Uganda-1, four in SSA1-SG1 ∪ SG2, and three in SSA2 ∪ SSA3. Among all absent orthologs, two were absent in all three African biological species (XP_018910005.1 and XP_018899251.1 in *B. argentifolii*). Orthologs of carboxylesterase 1E in *B. tabaci s.l.* (XP_018899849.1 in *B. argentifolii*), a highly conserved enzyme involved in xenobiotic resistance [82], were the only enzymes that clustered together with both outgroup species, *T. vaporariorum* and *Drosophila melanogaster*. This gene could not be detected in the genomes of *B. tabaci* SSA1-SG1 ∪ SG2.

#### Cytochrome P450 monooxygenases

P450s are a superfamily of enzymes that have essential roles in metabolic processes such as hormone synthesis and the catabolism of toxins and other chemicals in insects [83]. In *B. tabaci s.l.*, the activity of specific P450 enzymes was shown to confer resistance to chemical insecticides [84, 85]. Similar to other insects [86, 87], the cytochrome P450 (CYP) gene family can be divided into four major phylogenetic clans: CYP2, CYP3 (including families CYP6, CYP9, and CYP325), CYP4 (including families CYP4 and CYP325), and mitochondrial P450s (including families CYP12 and CYP314) (Additional File 1: Table S12, Table S13, Additional File 2: Fig. S11). Testing the presence/absence of *B. argentifolii* orthologs in the three African grouped biological species (Additional File 2: Fig. S11), indicated the absence of 33 orthologs in Uganda-1, mainly from the CYP3 (19 genes) and CYP4 (9 genes) clans. Also, 6 orthologs were found to be absent in SSA1-SG1 ∪ SG2 (3 in CYP3 and 3 in CYP4), and 10 in SSA2 ∪ SSA3 (7 in CYP3 and 2 in CYP4). From all orthologous missing in the African grouped biological species, two were absent both in SSA1-SG1 ∪ SG2 and SSA2 ∪ SSA3 (XP_018917660.1 and XP_018898101.1) and an additional two (XP_018905683.1 and XP_018917273.1) in all three African biological species.

#### Cytosolic sulfotransferases

SULTs constitute a group of enzymes that catalyze the transfer of a sulfonate group from the active sulfate, 3′-phosphoadenosine 5′-phosphosulfate, to a substrate compound containing a hydroxyl or amino group [88]. These enzymes are considered to be involved in the inactivation and excretion of xenobiotics and endogenous compounds [88]. In our analysis, we focused on two groups of sulfotransferases, SULT1E1 (six genes) and SULT1C4 (5 genes) (Additional File 1: Table S12, Table S13). Enzymes from the SULT1E1 group transfer a sulfonate group both to endogenous substrates such as estrogens or iodothyronines and to various flavonoids [89]. Enzymes from the SULT1C4 group modify steroids, neurotransmitters, and xenobiotics, and are involved in drug detoxification [90]. Testing the presence/absence of *B. argentifolii* orthologs in the three African biological species (Additional File 2: Fig. S12), indicated the absence of three orthologs in the Uganda-1 genome (two from SULT1C4 and one from SULT1E1). Also, orthologs from *T. vaporariorum* and *D. melanogaster* were only detected in the SULT1E1 group (Additional File 2: Fig. S12).

#### Glutathione S-transferases

Glutathione S-transferases of insects are essential to convert xenobiotics, such as toxic phytochemicals and synthetic insecticides, into nontoxic products [91]. In insects, this family is divided into two groups, microsomal enzymes and cytosolic enzymes, which differ both in their origin and structure [92]. The cytosolic GSTs are further divided into six classes: Theta, Zeta, Omega, Sigma, Delta, and Epsilon [93], the latter two being unique to insects [94]. Previous studies have shown that both the high tolerance of *B. tabaci* to insecticides and the species adaptability to plants with high levels of secondary metabolites, are associated with enhanced expression of GSTs [95–97]. Testing the presence/absence of *B. argentifolii* orthologs in the three African biological species (Additional File 1: Table S12, Table S13, Additional File 2: Fig. S13), indicated the absence of nine orthologs in Uganda-1 (eight from the Sigma and Delta-Epsilon classes), three Delta orthologs in SSA1-SG1 ∪ SG2 and one Delta class ortholog in SSA2 ∪ SSA3 (Additional File 2: Fig. S13). For all orthologous missing in the African biological species, only QHU79966.1 (Delta GST class) was absent in all three species. Interestingly, the Delta GST class was reported to play a role in the ability of insects to detoxify xenobiotics [98] and to be significantly expanded in *B. tabaci s.s.* [91].

#### Uridine diphosphate-glucuronosyltransferases

UGTs catalyze the addition of UDP-sugars to small hydrophobic molecules, turning them into more water-soluble metabolites [99]. In insects, UGTs play an essential role in the detoxification of xenobiotics and a variety of plant phytotoxins [99]. The phylogenetic analysis we conducted indicated that the UGT gene family of *B. tabaci* *s.l.* can be further divided into 15 subfamilies: (UGT352, UGT353, UGT354, UGT355, UGT356, UGT357, UGT358, UGT359, UGT360, UGT361, UGT362, UGT363, UGT365, UGT366, UGT50) [100, 101] (Additional File 1: Table S12,, Table S13 Additional File 2: Fig. S14). Testing the presence/absence of *B. argentifolii* orthologs in the three African biological species (Additional File 2: Fig. S14), indicated that 27 UGT orthologs are missing in Uganda-1, six in SSA1-SG1 ∪ SG2, seven in SSA2 ∪ SSA3, and three in both *B. tabaci s.s.* and Asia II-5. From the total of 33 absent orthologs, three could not be found in all three African biological species (XP_018914531.1, XP_018903292.1 and XP_018896850.1). Most absent genes belonged to only two subfamilies, *UGT353* (5/12) and *UGT352* (18/33). On the other hand, *T. vaporariorum* orthologs were found in all subfamilies except *UGT355*. Expansion of specific UGT subfamilies, *UGT352* and *UGT353*, was detected in *B. tabaci s.l.* (when compared to *T. vaporariorum*). Only one gene (XP_018897454.1 in *B. argentifolii*, subfamily *UGT50*) showed sufficient conservation to allow its clustering with orthologs from *D. melanogaster* and *T. vaporariorum* [102]. This UGT gene could not be found in the genomes of the *B. tabaci* SSA2 ∪ SSA3 and Uganda-1.

#### ATP-binding cassette transporters

ABC transporter genes encode membrane-bound proteins that carry a wide range of molecules such as amino acids, peptides, sugars, and a large number of hydrophobic compounds across membranes [103] and in *B. tabaci s.l.* are also implicated in insecticide resistance [104, 105]. The ABC transporters gene family of *B. tabaci s.l.* can be further sub-divided into eight subfamilies (A-H), which include an expanded ABC-G subfamily [105] (Additional File 1: Table S12, Table S13, Additional File 2: Fig. S15). Testing the presence/absence of *B. argentifolii* orthologs in the three African biological species (Additional File 2: Fig. S15), indicated that five orthologs (three ABC-G and two ABC-H) are missing in Uganda-1, and one ortholog from the ABC-G subfamily is missing in *B. tabaci* SSA1-SG1 ∪ SG2 and *B. tabaci* SSA2 ∪ SSA3, although not the same gene. *T. vaporariorum* orthologs were identified in all subfamilies, but *D. melanogaster* orthologs could be identified only for the ABC-G subfamily.

#### Evolution of α-glucosidase (GH13) within cassava *B. tabaci* SSA1-SG1-Ug and SSA1-SG1-Ng: a case of sucrose hydrolase

*B. tabaci s.l.* has evolved to exploit the sugars-rich diet of plant phloem-sap. Adaptations to this specialized diet include α-glucosidase genes that encode sugar-transforming enzymes belonging to the α-glucosidase glycoside hydrolase (GH) family 13 by hydrolyzing sugar to its constituent monosaccharides to facilitate both digestion and osmoregulation within the whitefly gut [106–108]. Alpha-glucosidases are categorized into three types (“I”, “II”, “III”), based on substrate recognition [109]. The α-glucosidase glycoside hydrolase (GH) family 13 type I is found in bacteria and insects [110]. These molecules recognize the α-glycosyl moiety and hydrolyze heterogeneous substrates such as sucrose. The phloem-sap of different plants contains varying concentrations of heterogeneous substrates/sugars, but sucrose is the dominant sugar present in many plants [111]. Recent studies have shown that the starch and sucrose metabolism pathways were overexpressed in *B. argentifolii* [74, 112]. This gene family and mechanism for sugar hydrolysis within different whitefly species, therefore, is likely to be important for host-plant adaptation.

In East and Central Africa, *B. tabaci* SSA1-SG1 ∪ SG2 reaches “super-abundant” numbers on cassava, however this phenomenon has not been reported for populations of the same species in West Africa [59]. To investigate whether, or not, this phenomenon is associated with evolutionary changes in the α-glucosidase GH13 genes, we analyzed these families in the sister populations *B. tabaci* SSA1-SG1-Ug and *B. tabaci* SSA1-SG1-Ng genomes and compared them to the non-cassava colonizing species *B. argentifolii*.

**Fig. 8:**
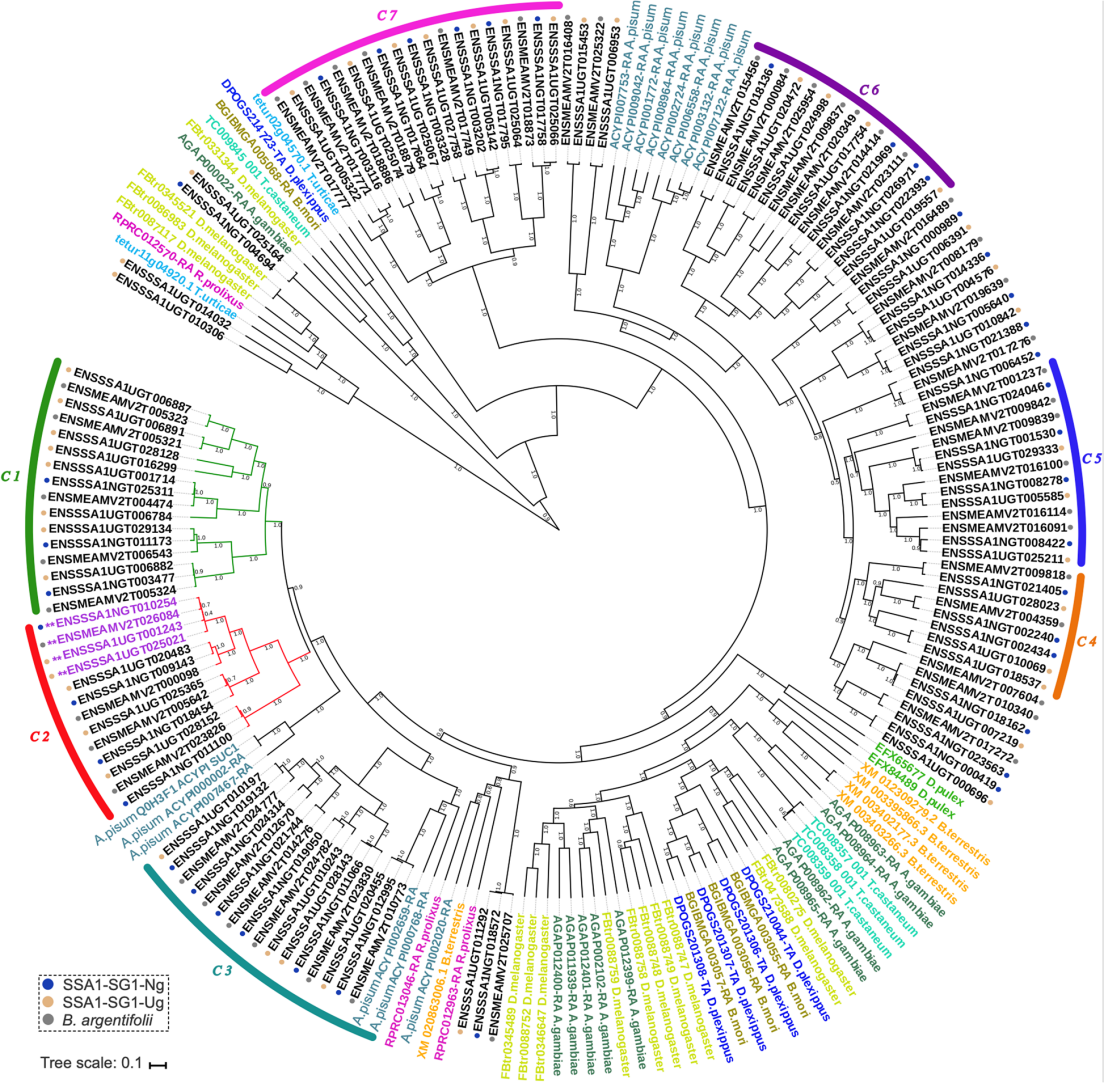
Phylogenetic relationships of α-glucosidase (GH-13) genes of thirteen arthropod species. Phylogenetic analysis focused on three *Bemisia tabaci s. l.* populations: SSA1-SG1-Ug, SSA1-SG1-Ng and *B. argentifolii*. Non-whitefly taxa *T. castaneum*, *A. pisum*, *A. gambiae*, *B. terrestris*, *B. mori*, *D. plexippus*, *D. pulex*, *D. melanogaster*, *R. prolixus* and *T. urticae* are uniquely colored. Phylogenetic analysis performed using a Bayesian approach and implemented in Bayesian Evolutionary Analysis Sampling Trees (BEAST version 1.10.2). Clusters (C 1 <—> C 7) are defined based on selection analysis; see Additional file 1: Table S14. The α-glucosidase genes related to sucrose hydrolysis are located in cluster 2, highlighted in purple (**)

Phylogenetic analysis of the 199 α-glucosidase (GH13) protein sequences identified in OGC ‘OG0000016’ resulted in clustering of 26 different clades (Fig. 8). In comparison to non-whitefly species, whitefly genomes had the highest number of α-glucosidase genes (Table 7), underlining their importance to this species group. The presence of signal peptides varied amongst all 199 sequences examined. Twenty-nine whitefly α-glucosidase genes clustered with the experimentally validated SUC1 gene ‘Q0H3F1_ACYPI’ (*A. pisum*), forming two clusters (cluster *C1*, highlighted in green & cluster *C2*, highlighted in red) (Fig. 8). The gene product ENSSSA1UGT001243 clustered with ENSSSA1NGT010254 and ENSMEAMV2T026084, SUC1 genes in *B. tabaci* SSA1-SG1-Ng and *B. argentifolii*, respectively. It also clustered with ENSSSA1UGT025021, a paralog in *B. tabaci* SSA1-SG1-Ug, although it lacked a signal peptide (Additional file 3). The implication is the gene ENSSSA1UGT025021 may encode for an enzymatically inactive protein or alternatively has become pseudogenized, as a signal peptide is required for the protein to function as an extracellular enzyme in the gut lumen [113]. These findings show that the vast majority of whitefly α-glucosidase genes clustered and were unique to whitefly. A small proportion had an orthologous relationship with genes belonging to *A. pisum*, indicating that these are also present in other phloem-sap feeders.

**Table 7:** The presence/absence of signal peptides in 13 arthropod populations/taxa

Populations and species	Total number of alpha-glucosidase	Alpha-glucosidase with signal peptide	Alpha-glucosidase without signal peptide
*B. argentifolii*	48	20	28
*B. tabaci* SSA1-SG1-Ug	48	28	20
*B. tabaci* SSA1-SG1-Ng	41	22	19
*Drosophila melanogaster*	14	13	1
*Acyrthosiphon pisum*	14	2	12
*Anopheles gambiae*	10	9	1
*Bombus terrestris*	5	4	1
*Danaus plexippus*	5	2	3
*Tribolium castaneum*	4	3	1
*Bombyx mori*	4	3	1
*Rhodnius prolixus*	3	3	0
*Daphnia pulex*	2	1	1
*Tetranychus urticae*	1	1	0

The numbers of α-glucosidase (GH13) with and without signal peptides for 13 populations of arthropod populations/taxa. Signal peptides are required for an enzyme to function as an extracellular enzyme in the gut lumen, so the presence of a signal peptide signifies that the protein is active or functional [113]

A total of four genes in *B. tabaci* SSA1-SG1-Ug and two genes in *B. tabaci* SSA1-SG1-Ng had orthologs in insect species that do not feed on phloem-sap. For example, three genes from *B. tabaci* SSA1-SG1-Ug, *B. tabaci* SSA1-SG1-Ng and *B. argentifolii* (ENSSSA1UGT011292, ENSSSA1NGT018572 & ENSMEAMV2T025707) clustered with RPRC013046-RA (*R. prolixus*), ACYP1002020-RA (*A. pisum*), XM020863006.1 (*B. terrestris*) and RPRCO12963-RA (*R. prolixus*). This gene encodes for a sucrose hydrolyase with two magnesium ion ligands binding to five amino acid residues; D.46, D.48, D.50, I.52 & D.54 and three protein–ligand interactions forming metal complexes with amino acid residues; D.48, D.50 and I.52. The second set of orthologous genes in *B. tabaci*, *Anopheles gambiae, D. melanogaster, Tribolium castaneum, Bombyx mori* and *Danaus plexippus* encode for heavy chain protein (neural and basic amino acid transport rBAT). These are heteromeric amino acids transporter b0, + AT-rBAT complex bound with arginine, and in SSA1-SG1-Ug and SSA1-SG1-Ng are coded by gene ENSSSA1UGT025164 and ENSSSA1NGT004694 respectively. Two SSA1-SG1-Ug genes (ENSSSA1UGT010306 & ENSSSA1UGT014032) were annotated as alpha-amylase, an enzyme that hydrolyze alpha bonds of large polysaccharide such as starch and glycogen. Alpha-amylases have also been previously reported in *B. tabaci* [114]. Among the studied insects, these genes were found only in *T. urticae*, *R. prolixus* and *D. melanogaster*.

To classify the α-glucosidases in *B. tabaci* SSA1-SG1-Ug and *B. tabaci* SSA1-SG1-Ng based on the approximate specificity, selected protein sequences were submitted to the protein structure homology-modeling server (Expasy webserver-SWISSMODEL) [115] to identify the best protein database (PDB) template for their three-dimensional structure modeling. We conclude that the α-glucosidase GH13 are: (i) sucrose hydrolyzing enzymes (with PDB: 6Igg.1.A, 6Iga.1.A, 6Igf.1.A as the best homologous PDB templates), (ii) toxin receptor proteins (PDB: 6K5p.1.A), (iii) neutral and basic amino acid transporter protein rBAT (PDB: 6li9.1.A) and (iv) alpha amylase (PDB: 1dhk.1.A, 1kxt.1.A). Of these, the largest numbers of proteins were toxin receptor proteins and sucrose hydrolyzing enzymes.

#### Selection pressure in alpha-glucosidases of East and West African *B. tabaci* SSA1-SG1

From examination of gene-tree clustering, we identified nine α-glucosidase genes in ‘cluster 2’ (Fig. 8) and these were chosen to investigate the nature of selection acting on these different populations of the same biological species of cassava whitefly. Each gene was analyzed for site selective pressure under both pervasive diversifying and purifying selection. Significant pervasive diversifying and episodic selection were detected in two genes: ENSSSA1NGT018454 and ENSSSA1UGT001243 (Table 8). Of these, ENSSSA1UGT001243 encodes for enzymes that hydrolyze sucrose in SSA1-SG1 cassava whitefly and when the sucrose hydrolases in ‘cluster 2’ (ENSSSA1UGT001243, ENSMEAMV2T026084 and ENSSSA1UGT025021) were compared, only ENSMEAMV2T026084 for *B. argentifolii* contained a protein–ligand interaction of two magnesium ions, interacting with ASP 48, ASP 50 and ILE 52 (Additional file 1: Table S15).

**Table 8:** Selection pressure analysis on the *Bemisia tabaci* SSA1-SG1 α-glucosidase (GH13) genes

Gene	Likelihood ratio test (LRT)	Number of sites under pervasive selection at P ≤ 0.01		Presence of episodic diversifying selection
		**Diversifying selection ( +)**	**Purifying selection (-)**	
ENSSSA1NGT018454	132.57	8	2	+
ENSSSA1UGT001243	16.08	4	0	+
ENSSSA1UGT028152	11.54	1	0	+
ENSSSA1UGT020483	2.39	1	0	-
ENSSSA1NGT009143	1.88	2	0	-
ENSSSA1NGT010254	1.06	1	0	-
ENSSSA1UGT025021	0.93	3	0	-
ENSSSA1UGT025365	0.00	0	0	-
ENSSSA1NGT011100	0.00	2	0	-

Analysis of selection pressure acting on the α-glucosidase (GH13) gene of *B. tabaci* SSA1-SG1-Ug and *B. tabaci* SSA1-SG1-Ng in “cluster 2”. The adaptive Branch-site Random Effects Likelihood (aBSREL) software analyzed diversifying selection using a branch-site effects model [116], while Fixed Effect Likelihood (FEL) software analyzed the number of sites within a gene with pervasive selection [117]. Three genes showed episodic diversifying selection indicating adaptive evolution

The *B. tabaci* SSA1-SG1-Ug gene ENSSSA1UGT001243 was identified using sequence-based methods. The gene encodes for a sucrase hydrolysis enzyme. Oligomeric modeling in SWISSMODEL identified PDB: 6k5p.1.A (a binary toxin receptor protein) and 6lga.1.A (sucrose hydrolase) as the two best quaternary structure annotations. The best model built for both ENSSSA1UGT001243 and its ortholog in *B. argentifolii* ENSMEAMV2T026084 (“Bta03818” [32]) sequence was that of a toxin receptor protein, while the second-best model predicted a sucrose hydrolase. Another study has reported some of the α-glucosidase GH13 acquire a secondary function, citing a toxin receptor as an example, primarily in mosquito species [118]. The results imply that ENSSSA1UGT001243 may possess two functions; sucrose hydrolysis (osmoregulation) and also a toxin receptor protein, which allows the *B. tabaci* SSA1-Ug to survive on many plant hosts [74, 112]. The toxin receptor proteins analyzed here possess a cadmium ion as a ligand, interacting with different amino acids and forming different ligand–protein interactions (Additional file 1: Table S15), highlighted by gene mutations in the number and nature of indels. We propose that the different attributes of these toxin receptor proteins enable the whitefly to deal with different phytotoxins, although experimental validation is still yet required.

To investigate if selection pressures have been relaxed or intensified for the East and West African populations of *B. tabaci* SSA1-SG1 ∪ SG2, the specific relaxation parameter (K) for each gene was determined. Examination of selection pressure In ‘cluster 1’, using the relaxation parameter (K) of both test branches (genes) shifted away from neutrality, with a branch-specific (K) of 1.15 and 1.53 for SSA1-SG1-Ug and SSA1-SG1-Ng, respectively (Additional file 2: Fig. S16a, S17a). In ‘cluster 2’, test branches in SSA1-SG1-Ug shifted towards neutrality, conversely SSA1-SG1-Ng shifted away from neutrality (K = 0.89 and 1.85, respectively) see Additional file 2: Fig. S16b, S17b. Genes evolving under relaxed selection, as seen in these SSA1-SG1-Ug α-glucosidases, facilitate organisms to respond adaptively to the changes in the environment [119]. This occurs either through the reduced intensity of both purifying and diversifying selection, which fosters evolutionary innovation or neofunctionalization, whereby one paralogous copy derives a new function after gene duplication [120]. Our results also show that the genes in ‘cluster 2’ (sugar homeostasis—osmoregulation genes) and ‘cluster 7’ (toxin receptor proteins – detoxification genes) in SSA1-SG1-Ug are under relaxed selection constraints, when compared with similar genes in SSA1-SG1-Ng (Additional file 1: Table S14, S15).

Since the epidemic of severe CMD began in Uganda in the 1990s, new varieties of cassava have been bred and released with CMD resistance. These varieties, however, proved highly susceptible to cassava whitefly [16, 17], allowing the *B. tabaci* SSA1-SG1-Ug population to adapt to this change in the ecological landscape. In this study, one of the genes in ‘cluster 2’, ENSSSA1UGT001243 (SUC1) has experienced episodic diversifying selection with four sites under pervasive diversifying selection (Table 8) and belongs to a cluster of genes that experienced relaxed selection. The combination of episodic diversifying and relaxed selection indicates that a selection force increased amino acid diversity of this *B. tabaci* SSA1-SG1-Ug *SUC1* gene, likely facilitating further evolutionary adaptations.

### Gene families associated with virus-vector competency

*Bemisia tabaci s.l.* evolution is linked closely to one of the groups of plant viruses that it transmits, the begomoviruses (genus *Begomovirus*, family *Geminiviridae*) [12]. These single-stranded DNA plant viruses are almost exclusively transmitted by *B. tabaci s.l.* and comprise one of the largest genera in the virosphere [121, 122]. Our goal was to elucidate the extent to which gain *vs* loss of important viral interaction gene families has occurred within *B. tabaci s.l.* genomes in order to further elucidate potential whitefly gene targets for interference studies. Our analysis of virus-vector competency gene families centered on sequence-based matching via conditional reciprocal best BLAST (CRBB), as well as manual curation of protein-coding functional annotation information. We expand on results by providing a comparative genomics framework (i.e., OGC gene tree generated via OrthoFinder) including specific reference to broader whitefly, and importantly *T. vaporariorum*. This approach was non-exhaustive, with further examination is warranted to verify these findings and fully elucidate these candidate gene family targets with potential for whitefly control.

Evasion of the host immune system is crucial for successful host infection and propagation of virus particles. Here we have examined the presence and diversity of peptidoglycan recognition proteins (PGRP) (IPR015510, IPR002502, IPR006619, IPR036505). PGRPs are a widely distributed, diverse family of proteins conserved between invertebrates and mammals which facilitate maintenance of vector-pathogen homeostasis via innate immune response against invading pathogens [123–126]. PGRPs exhibit gene-copy variability across Arthropoda. *D. melanogaster* (thirteen genes) contains a rich repertoire while mosquitos (seven genes) and tsetse flies (six genes) genomes contain fewer PGRPs [127–130].

To date, only a single PGRP gene ‘*BtPGRP’* (AJQ31845.1) has been identified in *B. argentifolii* [131]. In this study, comparative analysis revealed two independent PGRP-containing OGCs (‘OG0000284’ and ‘OG0003013’). OGC ‘OG0000284’, was composed of single copy PCGs across all genomes and contained proteins with high sequence identity to ‘*BtPGRP’* [131]. Wang et al. [131] reported that *BtPGRP* contained an Arg^106^ which is associated with recognizing *meso*-diaminopimelic acid (DAP)-type peptidoglycans, as previously shown for *D. melanogaster* PGRP-LE and PGRP-LC [132]. Our results confirm the presence of *BtPGRP in whitefly,* while further demonstrating its complete sequence conservation of Arg^106^ between all Aleyrodidae species examined and *D. melanogaster* PGRP-SB1 (FBtr0075348).

The second PGRP-like OGC ‘OG0003013’, contained proteins which exhibited functional domains linked to the ‘*N*-acetylmuramoyl-L-alanine amidase/PGRP domain’ superfamily (IPR002502). ‘OG0003013’ contained *B. tabaci s.l.* members harboring two gene copies each (SSA3-Ng, Uganda-1, *B. tabaci s.s*). However, not all Hemiptera examined (*A. pisum, M. persicae)* had detectable homologs within either of these two PGRP-like OGCs and highlights a possible PGRP gene loss over time within whitefly and more broadly Hemiptera.

The hsp70 family is the largest clade of *B. tabaci s.l.* heat shock proteins (HSPs) [133–136] and has been shown to play important roles in both insect development and begomovirus transmission for *B. argentifolii* [137, 138]. HSPs are highly conserved chaperone proteins, present in all organisms and cell types. They have diverse roles related, but not exclusively, to protein transport across membranes, cell cycle control, signaling, cellular stress responses and apoptosis [139]. Several studies have demonstrated that *B. tabaci s.l.* utilize HSPs to cope with thermal stress and that thermotolerance differs between cryptic species, possibly conferring ecological advantages [133, 140, 141]. An expanded analysis of these diverse sets of *hsp70*-related gene families is warranted but falls outside the scope of this investigation. We instead present a targeted examination of hsp70 protein domains (*IPR018181, IPR013126*, *IPR043129*) in *B. tabaci s.l* genomes.

A total of five OGCs (‘OG0000087’, ‘OG0002826’, ‘OG0004004’, ‘OG0004227’, ‘OG0004931’) with homology to HSP70 functional protein-domains were identified. Between 58–75% of the total gene members, across all *hsp70*-like OGCs identified across all *B. tabaci s.l.* genomes, were located within a single OGC ‘OG0000087’. Published hsp70 ‘*Bt*HSP70’ (*XP_018908958.1*) previously identified in *B. argentifolii* recovered significant CRBB hits to ‘OG0000087’ members. Our results showed hemipteran species exhibit the largest diversity of *hsp70*-like genes, with OGC ‘OG0000087’ containing up to fourteen HSP70 homologs per species; while *B. tabaci s.l.* genomes contained between 9–14 compared to 10 in *T. vaporariorum.*

Recent studies have demonstrated that for *B. tabaci s.l.,* the vesicle trafficking system of midgut cells plays an important role in the transport of begomoviruses across the midgut basal membrane to the haemolymph [142, 143]. Further work by Zhao et al. [144] identified the vesicle-associated membrane protein–associated protein B (VAPB) in *B. argentifolii*, which showed an inhibitory role in the transmission of the begomovirus tomato yellow leaf curl virus (TYLCV). Our investigation of VAPB-like proteins in *B. tabaci s.l.* genomes recovered six OGCs with homology to VAPB protein domains related to “Vesicle-associated membrane-protein-associated protein” (IPR016763), “PapD-like superfamily” (IPR008962) and “Major sperm protein” (MSP) domain (IPR000535). One of the six OGCs ‘OG0002935’ harbored members with high sequence identity to that of *B. argentifolii* VAPB (XP_018905345.1). All species were represented in ‘OG0002935’ by a single VAPB gene member, to the exclusion of *B. tabaci* SSA1-SG1-Ng and *B. tabaci* Asia II-5 which both had two copies. VAPB was not identified in *B. tabaci s.s.* Notably then, VAPB is potentially not vital for successful TYLCV transmission, given *B. tabaci s.s.* Is known to be an efficient vector of this virus [145].

Another crucial protein related to begomovirus transmission is found in *B. tabaci* midgut proteins (MGP). MGP is crucial for successful begomovirus transmission, where the viral coat protein interacts with the MGP to facilitate transfer into hemolymph and salivary glands [146]. MGP is characterized by the presence of a secretory signal-peptide and transcription activator MBF2 (IPR031734) domain. Two OGCs (‘OG0009507’, ‘OG0000655’) were identified with likely homology to this particular MBF2 domain, however only a subset of *B. tabaci s.l.* examined contained evidence of possessing a MBF2 signal peptide as previously reported by Rana et al. [146].

OGC ‘OG0009507’, had the most significant BLASTp hits to the previously reported uncharacterized protein in *B. argentifolii* (XP_018898813.1) and a secreted salivary protein (XP_026481543.1) identified in the cat flea *Ctenocephalides felis*. ‘OG0009507’ was composed of exclusively whitefly-only gene members, of which only Non-African *B. tabaci s.l.* members possessed a signal-peptide. *A. pisum* and *M. persicae* lacked any identifiable homologs to the MGP-like protein suggesting a this to be a uniquely whitefly-specific gene expansion with a potentially high degree of importance of this protein family during the co-evolution of begomoviruses and *B. tabaci s.l*. The second OGC ‘OG0000655’, had the highest overall homology to the MGP reported previously for *B. argentifolii* (AIK97534.1) [146]. Both OGCs represent excellent gene family targets for possible expansion of whitefly control mechanisms given their high degree of importance for begomovirus transmission.

Lastly, we examined the diversity of *B. tabaci s.l.* genes with homology to cyclophilins (CyPs). CyPs are a diverse superfamily of proteins found in both prokaryotes and eukaryotes. Cyps are involved in protein folding [147], and function in many cellular processes, such as protein–protein interactions and chaperone functionality (reviewed in [148]). CyPs have been implicated in luteovirus transport and transmission by aphids [149]. Whole genome comparative analysis based on query proteins with functional annotations linked to the cyclophilin-like domain superfamily (IPR029000), among others (IPR002130, IPR024936, IPR020892) resulted in recovery of fourteen OGCs containing CyP-like proteins. Three previously reported *B. tabaci s.l.* CyP proteins [150] were identified within our set of fourteen OGCs, specifically ‘CyPB’ (KX268377: ‘OG0003660’), ‘CyPD’ (KX268378: ‘OG0000333’), and ‘CyPG’ (KX268379: ‘OG0000792’). An average of 22.5 CyP-like proteins were discovered across all *B. tabaci s.l.* genomes (ranging from 16 to 28). In contrast only 17 PCGs were identified with CyP-like functionality in *T. vaporariorum*, which is unable to transmit TYLCV [151].

Amongst CyP gene families, CyPB was shown to have relatively increased gene expression after TYLCV acquisition by whitefly individuals [150], while also playing an important role in both insect development and the transmission of TYLCV by *B. argentifolii* [138, 150]. Generation of MSA of CyPB ‘OG0003660’ proteins highlighted extremely high sequence conservation within *B. tabaci s.l.* at ~ 96–99%, which decreased to 75–77% when considering all Aleyrodidae species. Crucial residues involved in CyPB cyclosporin A (CsA) binding and the reported CyPB signature peptidyl-prolyl *cis–trans* isomerase domain (‘YKGSKFHRVIKDFMIQGG’) had near complete sequence conservation across all whitefly. However, *T. vaporariorum* uniquely possessed a single substitution to the peptidyl-prolyl *cis–trans* isomerase domain (Tyr—> Phe) of the CsA binding domain. Of the fourteen CyP-like OGCs identified, only two OGCs ‘OG0009174’ and ‘OG0009922' were identified that appeared to be whitefly-specific, with no discernible sequence homology with any other taxon examined. Proteins in both OGCs had high sequence identity to *B. tabaci s.l.* PPIase B-like (LOC109043131) and PPIase B1-like (LOC109043200) proteins.

### Horizontally transferred genes

**Fig. 9:**
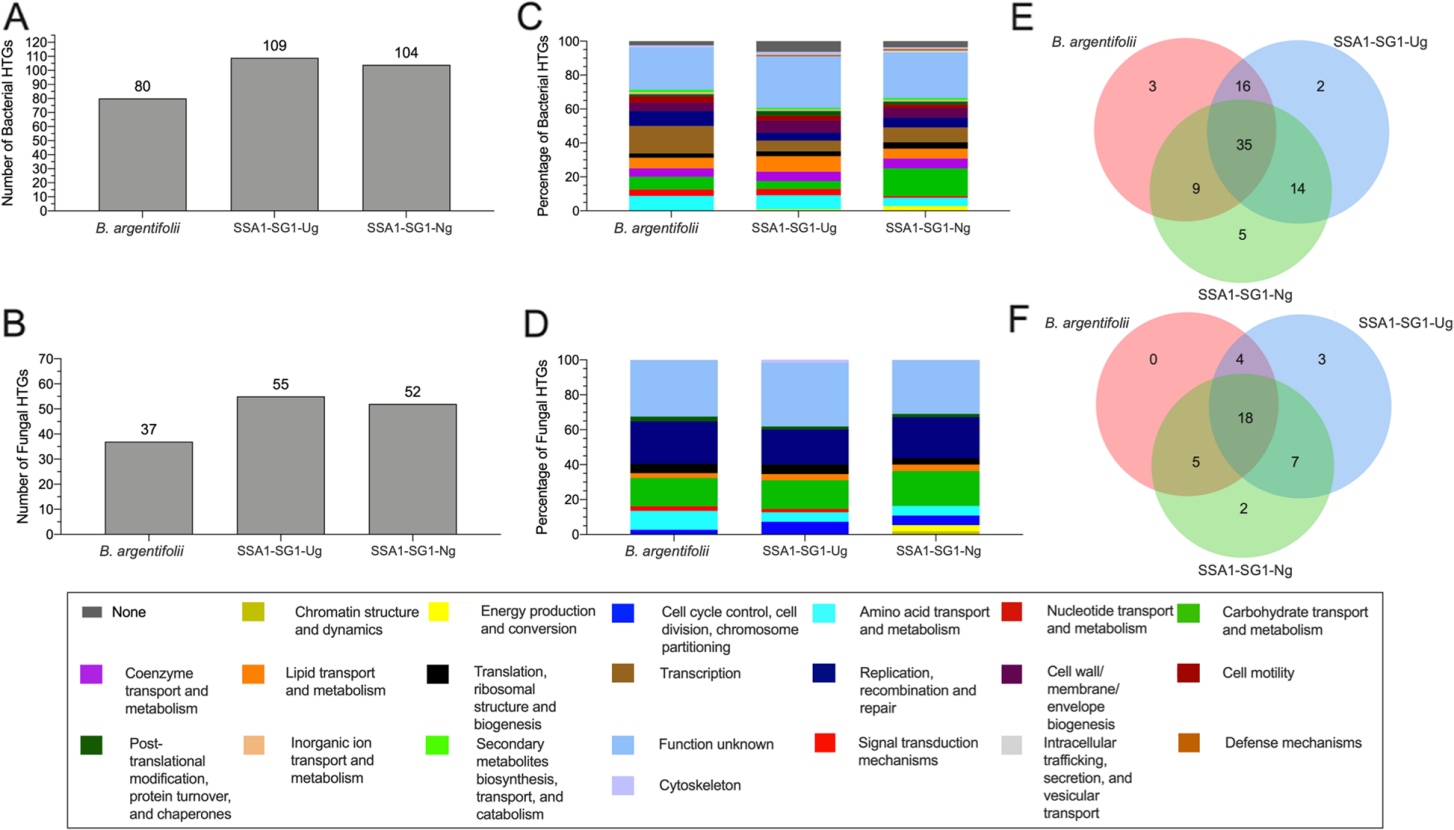
*Bemisia tabaci s.l.* horizontally transferred genes (HTGs) of bacterial and fungal origin. Total number of HTGs of: (**a**) bacterial and (**b**) fungal origin, derived from protein-coding transcripts. Relative proportion of functional predictions (COG categories) for: (**c**) bacterial and (**d**) fungal HTGs. Venn diagram of the distribution of HTG orthologous gene clusters (OGCs) among whitefly genomes for: (**e**) bacteria and (**f**) fungi

Genes originating from both bacteria and fungi are present in *B. tabaci s.l.* genomes, with 142 identified in the *B. argentifolii* draft genome [32]. Their functions involved hopanoid/sterol synthesis and xenobiotic detoxification enzymes that may play a role in polyphagy and insecticide resistance. Two adjacent bacterial pantothenate biosynthesis genes, panB and panC, were also reported to have been fused into a single gene that had acquired introns [32]. Protein-coding transcript analysis Additional file 1: Table S16 revealed that the *B. tabaci* SSA1-SG1 (i.e. SSA1-SG1-Ug, SSA1-SG1-Ng), *B. argentifolii* and *B. tabaci s.l.* genomes contained between 117 and 164 horizontally transferred genes (HTGs) of bacterial and fungal origins, respectively (Fig. 9a, b). These HTGs were predicted to encode proteins involved in conserved and different biological functions (Fig. 9c, d).

The functional profiles of HTGs of bacterial origin were similar in these genomes with most assigned to amino-acid transport and metabolism, carbohydrate transport and metabolism, lipid metabolism, transcription, and unknown functions (Fig. 9c). These bacterial HTGs grouped into 84 OGCs, 35 of which were shared between genomes. Twenty-one OGCs were specific to SSA1-SG1 (Fig. 9e). In addition, each genome contained two to five unique OGCs (Fig. 9e).

Similar to the bacterial HTGs, fungal HTGs were also very similar across the three genomes and enriched for functions related to carbohydrate, amino acid, and lipid metabolism, but also for other functions such as replication and recombination (Fig. 9d). Of the 39 OGCs, 18 were shared between genomes, 12 were present only in the SSA1-SG1 and none were specific to *B. argentifolii* (Fig. 9f). Although there was conservation in broad functional categories for HTGs, these data show that certain HTGs may have been either acquired or retained in a lineage-specific manner. These *B. tabaci s.l.* genomes were particularly enriched in HTGs, compared to other sap feeding Sternorrhyncha insects [32, 152]. Here, we compared the total number of HTGs and their predicted functions in *B. argentifolii* to those in *B. tabaci* SSA1-SG1 (Fig. 9) and found that HTG functional profiles were similar with most HTGs assigned to metabolic functions (Fig. 9b, e).

Despite nearly identical broad functional profiles, the acquisition and/or retention of some HTGs was lineage-specific (Fig. 9e, f). Differences in selective pressure due to abiotic stresses such as temperature, or biotic interactions such as host plant range, natural enemies and symbionts may underlie these differences. For example, the SSA1-SG1 lineage is particularly adapted to feed on cassava plants [31, 112], which may possess different toxic metabolites and/or phloem-sap composition compared to species within the host-plant range of *B. argentifolii*.

We also analyzed microbial HTGs beyond the comparative functional annotations of the SSA1-SG1 and *B. argentifolii* genomes. In total 78 (fungal) and 63 (bacterial) HTGs originally published in the genome of *B. argentifolii* were used as sequence queries for CRBB analysis to gene transcripts generated as part of this study (see Additional file 1: Table S16). Of the total fungal HTGs, surprisingly less than 40% were reliably recovered (pairwise sequence identity [PID] >  = 75%) in the annotations of seven of the eight genomes examined. Reannotation of *B. argentifolii* resulted in the recovery of only 30 (38.40%) of the original fungal HTGs previously reported for this genome. SSA1-SG1-Ng contained the most fungal HTGs (*n* = 33), but only fractionally more than the average of 26 observed across all eight genomes. Examination of bacterial HTGs showed similar levels of recovery across all *B. tabaci s.l.*, albeit at a slightly higher recovery rate than for fungal HTGs. We found that on average 43 bacterial HTGs were annotated across all *Bemisia* populations. *B. argentifolii* had 47 of the 63 bacterial HTGs originally reported, while again SSA1-SG1-Ng showed the highest overall recovery (*n* = 48).

Our re-examination of microbial horizontally transferred genes is consistent with the total number of ‘functionally distinct’ proteins previously published for *B. argentifolii.* We conclude that previous estimates of HTG diversity in *B. argentifolii* and *B. tabaci s.s.* genomes are likely to be overestimated and additional experimental investigation is warranted to characterize the precise diversity of *Bemisia* endosymbiont related HTG genes.

## Conclusions

Structural annotation of *B. tabaci s.l.* genomes applied via a uniform methodology, which excluded any *ab-initio* prediction component, has resulted in recovery of many fewer total PCG compared to previously published *B. tabaci s.l.* genomes. Though PCG are observed with only slight variances, to the exclusion of *B. tabaci s.s,* in the total number of recovered PCG across all eight *B. tabaci s.l* investigated*.* The finalized PCG sets of all genomes presented in this study constitute a conservative and well supported evaluation of the *B. tabaci s.l.* gene space.

Nuclear phylogenomics, supported by ML and BI recovered near universal support of both monophyletic whitefly (Aleyrodidae) and *B. tabaci s.l.* We show that the non-cassava utilizing species *B. tabaci* Uganda-1 resides as the earliest branching member of all African *Bemisia* examined in this study. Major relationships and topological branching order of sampled *Bemisia* species was again supported by independent analysis of 11 protein-coding mitochondrial genes. Reciprocal crossing experiments further corroborate these topological patterning, supporting the hypothesis that three biological species of *B. tabaci s.l.* colonize cassava in Africa, also adding to the weight of evidence that *B. tabaci s.l.* is a group of morphologically, highly cryptic biological species. Examination of average nucleotide identity and topological patterns among the primary obligate endosymbiont *Portiera* from *B. argentifolii*, *B. tabaci s.s*. and newly presented *B. tabaci s.l.* specifically grouped *B. tabaci* SSA2 and *B. tabaci* SSA3, as well as *B. tabaci* SSA1-Ng and *B. tabaci* SSA1-Ug together, further supporting their biological species’ statuses. Using an integrative systematics approach, we concluded that the seven, sub-Saharan, African cassava populations of *B. tabaci s.l.* can be reduced to just three biological species, namely *B. tabaci* SSA1-SG1 ∪ SG2, *B. tabaci* SSA2 ∪ SSA3 and *B. tabaci* SSA1-SG3. The former two biological species occur widely in both East and West Africa, so are most usefully identified currently by names that include (molecular) diagnostic information.

These new *Bemisia* genomes will make a significant contribution to resources for studies of *B. tabaci s.l.* comparative evolution and for resolving the number of biological species within this morphologically cryptic group. They shall also assist efforts to unravel the genetics behind species’ differences in their interactions with host plants, invasiveness, and propensity to cause outbreaks. Lastly, this study contributes towards the expansion of valuable genetic information across multiple species of *Bemisia* further enabling the selection of candidate gene targets for novel whitefly and plant-virus control methods.

## Materials and methods

See Additional file 4 for full details of methods employed in this study.

### De novo genome assembly

Prior to assembly, input PacBio read data was screened taxonomically to remove contaminants (Additional file 1: Table S2) using a k-mer approach [153, 154]. Initial draft assemblies were generated with Canu v1.8 [37]. A priori Canu consensus correction genome size estimate was set to 650 Mb (genomeSize = 0.650 g). Canu ‘correctedErrorRate’ (0.045) value for consensus read overlap correction was adjusted whereby *correctedErrorRate* was decreased when genomic coverage was estimated to be > 60X. A genome refinement protocol was applied to each draft assembly and proceeded by initial generation of a ‘polished’ genome consensus via alignment of input read corrected reads using minimap2 [155] and wtdbg2-cns [156]. Redundans [157] was used to account for uncollapsed haplotypic variation. Collapsed haplotype draft references were then further scaffolded with PBSuite [158] and then screened for contamination using blobtools [159, 160]. See Additional file 4 and Additional file 1: Table S3 for full detail on de novo assembly and genome draft refinement.

### Structural and functional annotation of *B. tabaci s. l. genomes*

Genomic annotations of *B. tabaci s.l.* were generated via the EMBL-EBI Ensembl Gene Annotation pipeline [46]. Ensembl transcript models generated are supported by experimental evidence in the form of aligned short-read RNA-seq data, then verified and filtered using secondary protein-to-genome alignments of proteins from whitefly or closely related species. The Ensembl Gene Annotation pipeline gave precedence to gene models supported by well-aligned transcriptomic evidence [46, 161]. The genomes of *B. argentifolii* and *B. tabaci s.s.* were also reannotated (utilizing their original published RNA-Seq datasets). Bacteriocyte-specific RNA-seq libraries were applied to ensure bacteriocyte-specific gene expression contributed to structural annotation. Finalized gene builds contain multi-transcript PCG structures, where each transcript can contain overlapping exon boundaries; excluding any redundant transcripts where the splicing pattern is completely redundant when comparing to a longer model [46]. See Additional file 4 for complete detail of genomic annotation methods. Core databases of the six newly generated *B. tabaci s.l.* genomes presented herein were first released in April-2021, via Ensembl Metazoa (Ensembl release version e103) (https://metazoa.ensembl.org/). Re-annotations of both *B. argentifolii* and *B. tabaci s.s.* are available as a standalone ‘Figshare’ dataset (FastA, GFF3) [162] as part of this study.

Interproscan [163] version 5.40–77.0 (https://github.com/ebi-pf-team/interproscan) was used to generate functional annotations of canonical PCG gene models. Data was then exported in TSV format, then parsed to obtain only gene models that were annotated with Gene Ontology GO terms. This data was then imported into the web service WEGO 2.0 [164, 165]. Tabular data was exported from WEGO 2.0 in CSV format. Graphical data was output in PNG format, selecting only GO:term relationships deemed to be significant. Significance was assessed by the statistical Chi-square (*P*-value < 0.05) test of independence of expected frequencies of genes with GO terms and their observed frequencies.

### Comparative genomics, phylogenomics and target gene family identification

Orthologous sequences of twenty-three arthropod species (*n* = 23) were identified and clustered via OrthoFinder (v2.4.0) [51, 52] using only canonical PCGs for input (Table 6). Representatives of all six *B. tabaci s. l.* presented in this study were included, but also the published whitefly *B. argentifolii* and *B. tabaci s.s* [32, 33]*.* utilizing PCGs annotated via Ensembl gene annotation pipeline specifically for their inclusion in this study [162]. Protein-coding sequences for the closely related species *T. vaporariorum* were downloaded from WhiteflyDB (http://www.whiteflygenomics.org). *T. vaporariorum* [43] served as the closest outgroup taxon available to the *Bemisia* genus and was the only other Aleyrodidae family member with a complete genome assembly and annotation available at the time of analysis. See Table 6 for details on species analyzed, their data sources and results of homology clustering in the comparative whole genome analysis.

Protein sequences of OGCs (only OGCs >  = 78.30% of all species considered) were aligned using MAFFT v7.427 [166]. The concatenated alignment was cleaned using Gblocks v0.91b [167]. Evolutionary model testing was performed via ProtTest3 [168], allowing for mixed rate alpha ‘α’ and also invariant sites selection ‘Ι’. Phylogenetic tree reconstruction was performed under ML framework (RAxML v8.2.12) [169] and BI (MrBayes v3.2.7a) [170, 171] methods. The best fitting model of protein evolution (LG + Γ + I + F) was selected in RAxML using the default parameters including ‘-m PROTGAMMAILGF -p 12,345’. A MrBayes run was implemented with ‘lset rates = invgamma ngammacat = 4; prset aamodelpr = fixed(lg)’. BI was performed with four chains for 1,000,000 generations, posterior probability (PP) and consensus topology were summarized using MrBayes ‘sump’ and ‘sumt’; burnin was set to 25% of input sample trees.

Prior to all gene family analyses, a genome-wide screening method was adopted to identify orthologs of gene families of interest. These candidate OGCs were identified via a combination of screening of functional protein/domain annotation obtained from InterProScan v -5.40–77.0 and sequence-based matching using CRBB [163, 172] for full details see Additional file 4.

### TE annotation

Transposable element (TE) libraries generated RepeatModeler (https://www.repeatmasker.org/RepeatModeler/) v1.73. To avoid ‘over-masking’ of protein encoding genomic loci, de novo TE repeat libraries were screened with BLASTp and BLASTn MegaBLAST [173, 174]. The protocol for filtering potential protein containing repeat sequences is described here (https://blaxter-lab-documentation.readthedocs.io/en/latest/filter-repeatmodeler-library.html) and was implemented via bash script. Genome-wide copy number variation and overall sequence divergence on TE repeat types was examined using the Perl utility ‘OneCodetofindthemall’ [175] v.1.0 (https://doua.prabi.fr/software/one-code-to-find-them-all), and statistics reported on a per genome scaffold basis. To summarize TE repeat content ‘OneCodetofindthemall’ output was parsed using an in-house Perl script taking ‘.copynumber.csv’ files as input. Kimura divergence scores were extracted from RM generated ‘.out’ output file using the protocol as described [48], using custom Kimura divergence scripts (bash, R, Python) freely available via [48].

### Reciprocal cross-mating

*B. tabaci s.l* populations from Nigeria and Uganda were subjected to reciprocal crossing experiments in sympatric and allopatric crosses. Sympatric populations cohabitated in the same field or had overlapping geographic ranges, while allopatric populations had separate, non-overlapping ranges or were separated by more than 500 miles [31, 59]. The following population combinations were classified as sympatric (i) SSA1-SG1-Ng X SSA3-Ng, (ii) SSA2-Ug X SSA1-SG1-Ug, (iii) SSA2-Ng X SSA3-Ng and (iv) SSA1-SG1-Ng X SSA2-Ng. Allopatric population combinations were (i) SSA1-SG1-Ng X SSA1-SG1-Ug, (ii) SSA1-SG1-Ng X SSA2-Ug and (iii) SSA2-Ug X SSA3-Ng. See [8] and Additional file 4 for details of the crossing methods used.

### *Portiera* genome assembly and comparative genomics

*Portiera* genomes were obtained using an iterative-assembly approach (flye_iterative.sh available as part of data file deposited in ‘Figshare’ [176]). Briefly, Kraken2 v2.1.2 [154] with the database PlusPF-16 (standard Kraken2 database plus the protozoa, fungi, and plant RefSeq databases, available from (https://genome-idx.s3.amazonaws.com/kraken/k2_pluspf_16gb_20210517.tar.gz) was used to perform an initial selection of *Portiera* sequences from the pre-processed PacBio sequences (non-taxonomically filtered). Kraken-tools v1.2 was used to extract any classified sequence below the Oceanospirillales rank. Primary assemblies of *Portiera* sequences were produced with flye [177] v2.8.3-b1695 (–pacbio-raw –genome-size 0.35 m –meta). Primary assemblies were used to recover possible missing *Portiera* reads with minimap2 v2.17-r941 (-x map-pb) [155] and the pre-processed PacBio sequences. Secondary assemblies were obtained using all sequences recovered with minimap2 and re-assembled with flye (–pacbio-raw –genome-size 0.35 m –meta). Finally, secondary assemblies were polished with fly (-pacbio-raw –genome-size 0.35 m –meta –polish-target –iterations 10).

Polished *Portiera* assemblies were annotated with prokka v1.14.6 [178] (–gram neg –rfam), using all available *Portiera* genomes as primary annotation source (–proteins). Obtained annotations were manually inspected in Artemis v1.5 [179]. Annotations and genome sequences provided in GenBank format (.gbk), available as part of a ‘Figshare’ dataset [176] deposited as part of this study.

Orthologous clusters of proteins (OCPs) were computed with OrthoFinder v2.4.0 (-M msa -T iqtree -I 1.5) [51]. Encoding potential of *Portiera* strains was manually assessed using *Portiera* MED-Q1 (CP003835) as reference. Genomic synteny among *Portiera* genomes was plotted with genoPlotR [180] based on 202 core OCPs and the three proteins encoded in the variable region (*yidC, mnmE,* and *mnmG*).

Pairwise Average Nucleotide Identity values were calculated with the enveomics toolbox [181] using USEARCH v11.0.667 (ublast -id 0.1 -maxhits 1,000—acceptall -evalue 1e-5 -accel 1) as alignment algorithm [182]. The heatmap and hierarchical clustering (Euclidean distances and complete clustering) were obtained with the gplots package from R [183].

## Supplementary Information

**Additional file 1. **Supplementary tables S1-S16.

**Additional file 2. **Supplementary figures S1-S17.

**Additional file 3. **SignalP results for the α-glucosidase (GH13) of selected insect species.

**Additional file 4. **Supplementary text.

## Data Availability

The genomes, transcriptomes, and predicted protein-coding sequences are available from Ensembl Metazoa (http://metazoa.ensembl.org) and are included within the references. Raw RNA-Seq datasets generated and/or analyzed during the current study are available from the European Nucleotide Archive database repository (https://www.ebi.ac.uk/ena) under the parent project accessions: PRJEB28507, PRJEB36965, PRJEB35304, PRJEB39408. All data generated during the analyses of these datasets are included in this published article, supplementary information files, and figshare repository (10.6084/m9.figshare.23666799; 10.6084/m9.figshare.23666832.v4;  10.6084/m9.figshare.23666844). Custom Perl scripts are available from the authors on request.

## Abbreviations

API: Application programming interface
BI: Bayesian inference
BS: Bootstrap
BUSCO: Benchmarking universal single-copy orthologs
cDNA: Complementary DNA
CMD: Cassava mosaic disease
CBSD: Cassava brown streak disease
ENA: European Nucleotide Archive
GC: Guanine-cytosine
GO: Gene ontology
Hi-C: High-throughput chromosome conformation capture
HTG: Horizontally transferred gene
lncRNA: Long non-coding RNA
LINE: Long interspersed nuclear element
LTR: Long terminal repeat
ML: Maximum likelihood
MSA: Multiple sequence alignment
mtCO1: Mitochondrial cytochrome* c* oxidase subunit I
OGC: Orthologous gene cluster
P450: Cytochrome P450 monooxygenase
PCG: Protein-coding gene
PE: Paired-end
PID: Pairwise sequence identity
PP: Posterior probability
RNA-Seq: RNA sequencing
RM: RepeatMasker
rRNA: Ribosomal RNA
RT: Reverse transcriptase
scaRNA: Small Cajal body-specific RNA
SD: Standard deviation
SINE: Short interspersed nuclear element
snoRNA: Small nucleolar RNA
TE: Transposable element
TYLCV: Tomato yellow leaf curl virus
UTR: Untranslated region
Y2H: Yeast two-hybrid

## References

[1] Bellows TS, Perring TM, Gill RJ, Headrick DH (1994). Description of a species of *Bemisia* (Homoptera: Aleyrodidae). Ann Entomol Soc Am.

[2] Tay WT, Evans GA, Boykin LM, De Barro PJ (2012). Will the real *Bemisia tabaci* please stand up?. PLoS ONE.

[3] Russell LM (1957). Synonyms of *Bemisia tabaci* (Gennadius)(Homoptera: Aleyrodidae). Bull Brooklyn Entomol Soc.

[4] Frohlich DR, Torres-Jerez I, Bedford ID, Markham PG, Brown JK (1999). A phylogeographical analysis of the *Bemisia tabaci* species complex based on mitochondrial DNA markers. Mol Ecol.

[5] Liu S, Colvin J, De Barro PJ (2012). Species concepts as applied to the whitefly *Bemisia tabaci* systematics: how many species are there?. J Integr Agric.

[6] Vyskočilová S, Tay WT, van Brunschot S, Seal S, Colvin J (2018). An integrative approach to discovering cryptic species within the *Bemisia tabaci* whitefly species complex. Sci Rep.

[7] Mugerwa H, Seal S, Wang H-L, Patel MV, Kabaalu R, Omongo CA (2018). African ancestry of New World, *Bemisia tabaci*-whitefly species. Sci Rep.

[8] Mugerwa H, Wang H-L, Sseruwagi P, Seal S, Colvin J (2021). Whole-genome single nucleotide polymorphism and mating compatibility studies reveal the presence of distinct species in sub-Saharan Africa *Bemisia tabaci* whiteflies. Insect Sci.

[9] De Barro PJ, Liu S-S, Boykin LM, Dinsdale AB (2011). *Bemisia tabaci*: a statement of species status. Annu Rev Entomol.

[10] Maruthi MN, Colvin J, Thwaites RM, Banks GK, Gibson G, Seal SE (2004). Reproductive incompatibility and cytochrome oxidase I gene sequence variability amongst host-adapted and geographically separate *Bemisia tabaci* populations (Hemiptera: Aleyrodidae). Syst Entomol.

[11] Lowe S, Browne M, Boudjelas S, De Poorter M. 100 of the world’s worst invasive alien species: a selection from the global invasive species database. Auckland: Invasive Species Specialist Group Auckland; 2000.

[12] Navas-Castillo J, Fiallo-Olivé E, Sánchez-Campos S (2011). Emerging virus diseases transmitted by whiteflies. Annu Rev Phytopathol.

[13] Gilbertson RL, Batuman O, Webster CG, Adkins S (2015). Role of the insect supervectors *Bemisia tabaci* and *Frankliniella occidentalis* in the emergence and global spread of plant viruses. Annu Rev Virol.

[14] Otim-Nape GW, Bua A, Thresh JM, Baguma Y, Ogwal S, Ssemakula GN, et al. The current pandemic of cassava mosaic virus disease in East Africa and its control. Chatham, UK: Natural Resources Institute; 2000.

[15] Colvin J, Omongo CA, Maruthi MN, Otim-Nape GW, Thresh JM (2004). Dual begomovirus infections and high *Bemisia tabaci* populations: two factors driving the spread of a cassava mosaic disease pandemic. Plant Pathol.

[16] Omongo CA, Kawuki R, Bellotti AC, Alicai T, Baguma Y, Maruthi MN (2012). African cassava whitefly, *Bemisia tabaci*, resistance in African and South American cassava genotypes. J Integr Agric.

[17] Macfadyen S, Tay WT, Hulthen AD, Paull C, Kalyebi A, Jacomb F (2021). Landscape factors and how they influence whitefly pests in cassava fields across East Africa. Landsc Ecol.

[18] Maruthi MN, Hillocks RJ, Mtunda K, Raya MD, Muhanna M, Kiozia H (2005). Transmission of *Cassava brown streak virus* by *Bemisia tabaci* (Gennadius). J Phytopathol.

[19] Colvin J, Omongo CA, Govindappa MR, Stevenson PC, Maruthi MN, Gibson G (2006). Host-plant viral infection effects on arthropod-vector population growth, development and behaviour: management and epidemiological implications. Adv Virus Res.

[20] Alicai T, Omongo CA, Maruthi MN, Hillocks RJ, Baguma Y, Kawuki R (2007). Re-emergence of cassava brown streak disease in Uganda. Plant Dis.

[21] Patil BL, Legg JP, Kanju E, Fauquet CM (2015). Cassava brown streak disease: a threat to food security in Africa. J Gen Virol.

[22] Nweke FI (1994). Cassava processing in sub-Saharan Africa: the implications for expanding cassava production. Outlook Agric.

[23] Hahn SK, Janet K (1985). Cassava: a basic food of Africa. Outlook Agric.

[24] Thresh JM, Otim-Nape GW, Legg JP, Fargette D (1997). *African cassava mosaic virus* disease: the magnitude of the problem. Afr J Root Tuber Crops.

[25] Legg JP, Shirima R, Tajebe LS, Guastella D, Boniface S, Jeremiah S (2014). Biology and management of *Bemisia* whitefly vectors of cassava virus pandemics in Africa. Pest Manag Sci.

[26] Dinsdale A, Cook L, Riginos C, Buckley YM, De Barro P (2010). Refined global analysis of *Bemisia tabaci* (Hemiptera: Sternorrhyncha: Aleyrodoidea: Aleyrodidae) mitochondrial cytochrome oxidase 1 to identify species level genetic boundaries. Ann Entomol Soc Am.

[27] Ally HM, El Hamss H, Simiand C, Maruthi MN, Colvin J, Omongo CA (2019). What has changed in the outbreaking populations of the severe crop pest whitefly species in cassava in two decades?. Sci Rep.

[28] Wosula EN, Chen W, Fei Z, Legg JP (2017). Unravelling the genetic diversity among cassava *Bemisia tabaci* whiteflies using NextRAD sequencing. Genome Biol Evol.

[29] Chen W, Wosula EN, Hasegawa DK, Casinga C, Shirima RR, Fiaboe KK (2019). Genome of the African cassava whitefly *Bemisia tabaci* and distribution and genetic diversity of cassava-colonizing whiteflies in Africa. Insect Biochem Mol Biol.

[30] Elfekih S, Tay WT, Polaszek A, Gordon KHJ, Kunz D, Macfadyen S (2021). On species delimitation, hybridization and population structure of cassava whitefly in Africa. Sci Rep.

[31] Mugerwa H, Colvin J, Alicai T, Omongo CA, Kabaalu R, Visendi P, et al. Genetic diversity of whitefly (*Bemisia* spp.) on crop and uncultivated plants in Uganda: implications for the control of this devastating pest species complex in Africa. J Pest Sci. 2021;:1–24. 10.1007/s10340-021-01355-6 PMC8550740 34720787

[32] Chen W, Hasegawa DK, Kaur N, Kliot A, Pinheiro PV, Luan J (2016). The draft genome of whitefly *Bemisia tabaci* MEAM1, a global crop pest, provides novel insights into virus transmission, host adaptation, and insecticide resistance. BMC Biol.

[33] Xie W, Chen C, Yang Z, Guo L, Yang X, Wang D (2017). Genome sequencing of the sweetpotato whitefly *Bemisia tabaci* MED/Q. GigaScience.

[34] Zachos FE (2018). Mammals and meaningful taxonomic units: the debate about species concepts and conservation. Mammal Rev.

[35] De Queiroz K (2007). Species concepts and species delimitation. Syst Biol.

[36] Wongnikong W, van Brunschot SL, Hereward JP, Barro PJD, Walter GH (2020). Testing mate recognition through reciprocal crosses of two native populations of the whitefly *Bemisia tabaci* (Gennadius) in Australia. Bull Entomol Res.

[37] Koren S, Walenz BP, Berlin K, Miller JR, Bergman NH, Phillippy AM (2017). Canu: scalable and accurate long-read assembly via adaptive k-mer weighting and repeat separation. Genome Res.

[38] Challis R. rjchallis/assembly-stats 17.02. Zenodo. 2017. 10.5281/zenodo.322347.

[39] Bernardi G (2007). The neoselectionist theory of genome evolution. Proc Natl Acad Sci.

[40] Seppey M, Manni M, Zdobnov EM. BUSCO: assessing genome assembly and annotation completeness. In: Kollmar M, editor. Gene Prediction. New York, NY: Springer New York; 2019. p. 227–45. 10.1007/978-1-4939-9173-0_14 31020564

[41] Kriventseva EV, Kuznetsov D, Tegenfeldt F, Manni M, Dias R, Simão FA (2019). OrthoDB v10: sampling the diversity of animal, plant, fungal, protist, bacterial and viral genomes for evolutionary and functional annotations of orthologs. Nucleic Acids Res.

[42] Blackman RL, Cahill M (1998). The karyotype of *Bemisia tabaci* (Hemiptera: Aleyrodidae). Bull Entomol Res.

[43] Xie W, He C, Fei Z, Zhang Y (2020). Chromosome-level genome assembly of the greenhouse whitefly (*Trialeurodes vaporariorum* Westwood). Mol Ecol Resour.

[44] Florea L, Souvorov A, Kalbfleisch TS, Salzberg SL. Genome assembly has a major impact on gene content: a comparison of annotation in two *Bos taurus* assemblies. PLOS ONE. 2011;6. 10.1371/journal.pone.0021400 PMC3120881 21731731

[45] Simão FA, Waterhouse RM, Ioannidis P, Kriventseva EV, Zdobnov EM (2015). BUSCO: assessing genome assembly and annotation completeness with single-copy orthologs. Bioinformatics.

[46] Aken BL, Ayling S, Barrell D, Clarke L, Curwen V, Fairley S, et al. The Ensembl gene annotation system. Database. 2016;2016. 10.1093/database/baw093 PMC4919035 27337980

[47] Bourque G, Burns KH, Gehring M, Gorbunova V, Seluanov A, Hammell M (2018). Ten things you should know about transposable elements. Genome Biol.

[48] Whitfield ZJ, Dolan PT, Kunitomi M, Tassetto M, Seetin MG, Oh S (2017). The diversity, structure, and function of heritable adaptive immunity sequences in the *Aedes aegypti* genome. Curr Biol CB.

[49] Petersen M, Armisén D, Gibbs RA, Hering L, Khila A, Mayer G (2019). Diversity and evolution of the transposable element repertoire in arthropods with particular reference to insects. BMC Evol Biol.

[50] Sicat JPA, Visendi P, Sewe SO, Bouvaine S, Seal SE (2022). Characterization of transposable elements within the *Bemisia tabaci* species complex. Mob DNA.

[51] Emms DM, Kelly S. OrthoFinder: phylogenetic orthology inference for comparative genomics. Genome Biol. 2019;20. 10.1186/s13059-019-1832-y PMC6857279 31727128

[52] Emms DM, Kelly S (2015). OrthoFinder: solving fundamental biases in whole genome comparisons dramatically improves orthogroup inference accuracy. Genome Biol.

[53] Campbell LI, van Brunschot SL, Nwezeobi J. Additional file 5: Comparative genomic analysis of *Bemisia tabaci s.l. *and other arthropods. Figshare [dataset]. 2023. 10.6084/m9.figshare.23666799.

[54] Campbell LI, Rota-Stabelli O, Edgecombe GD, Marchioro T, Longhorn SJ, Telford MJ (2011). MicroRNAs and phylogenomics resolve the relationships of Tardigrada and suggest that velvet worms are the sister group of Arthropoda. Proc Natl Acad Sci.

[55] Giribet G, Edgecombe GD (2019). The phylogeny and evolutionary history of arthropods. Curr Biol.

[56] Stoll NR, Shull AF (1919). Sex determination in the white fly. Genetics.

[57] Qin L, Pan L-L, Liu S-S (2016). Further insight into reproductive incompatibility between putative cryptic species of the *Bemisia tabaci* whitefly complex. Insect Sci.

[58] Hsieh C-H, Ko C-C, Chung C-H, Wang H-Y (2014). Multilocus approach to clarify species status and the divergence history of the *Bemisia tabaci* (Hemiptera: Aleyrodidae) species complex. Mol Phylogenet Evol.

[59] Nwezeobi J, Onyegbule O, Nkere C, Onyeka J, van Brunschot S, Seal S (2020). Cassava whitefly species in eastern Nigeria and the threat of vector-borne pandemics from East and Central Africa. PLoS ONE.

[60] Brown WM, George M, Wilson AC (1979). Rapid evolution of animal mitochondrial DNA. Proc Natl Acad Sci.

[61] Bing X-L, Yang J, Zchori-Fein E, Wang X-W, Liu S-S (2013). Characterization of a newly discovered symbiont of the whitefly *Bemisia tabaci* (Hemiptera: Aleyrodidae). Appl Environ Microbiol.

[62] El Hamss H, Ghosh S, Maruthi MN, Delatte H, Colvin J (2021). Microbiome diversity and reproductive incompatibility induced by the prevalent endosymbiont *Arsenophonus* in two species of African cassava *Bemisia tabaci* whiteflies. Ecol Evol.

[63] Baumann P (2005). Biology of bacteriocyte-associated endosymbionts of plant sap-sucking insects. Annu Rev Microbiol.

[64] Thao ML, Baumann P (2004). Evolutionary relationships of primary prokaryotic endosymbionts of whiteflies and their hosts. Appl Environ Microbiol.

[65] Santos-Garcia D, Vargas-Chavez C, Moya A, Latorre A, Silva FJ (2015). Genome evolution in the primary endosymbiont of whiteflies sheds light on their divergence. Genome Biol Evol.

[66] Santos-Garcia D, Latorre A, Moya A, Gibbs G, Hartung V, Dettner K (2014). Small but powerful, the primary endosymbiont of moss bugs, *Candidatus* Evansia muelleri, holds a reduced genome with large biosynthetic capabilities. Genome Biol Evol.

[67] Tamas I, Wernegreen JJ, Nystedt B, Kauppinen SN, Darby AC, Gomez-Valero L (2008). Endosymbiont gene functions impaired and rescued by polymerase infidelity at poly (A) tracts. Proc Natl Acad Sci.

[68] Santos-Garcia D, Mestre-Rincon N, Ouvrard D, Zchori-Fein E, Morin S (2020). *Portiera* gets wild: genome instability provides insights into the evolution of both whiteflies and their endosymbionts. Genome Biol Evol.

[69] Konstantinidis KT, Tiedje JM (2005). Genomic insights that advance the species definition for prokaryotes. Proc Natl Acad Sci.

[70] Sloan DB, Moran NA (2013). The evolution of genomic instability in the obligate endosymbionts of whiteflies. Genome Biol Evol.

[71] Santos-Garcia D, Juravel K, Freilich S, Zchori-Fein E, Latorre A, Moya A (2018). To B or not to B: comparative genomics suggests *Arsenophonus* as a source of B vitamins in whiteflies. Front Microbiol.

[72] Wang Y-B, Ren F-R, Yao Y-L, Sun X, Walling LL, Li N-N (2020). Intracellular symbionts drive sex ratio in the whitefly by facilitating fertilization and provisioning of B vitamins. ISME J.

[73] Li N-N, Jiang S, Lu K-Y, Hong J-S, Wang Y-B, Yan J-Y (2022). Bacteriocyte development is sexually differentiated in *Bemisia tabaci*. Cell Rep.

[74] Malka O, Santos-Garcia D, Feldmesser E, Sharon E, Krause-Sakate R, Delatte H (2018). Species-complex diversification and host-plant associations in *Bemisia tabaci*: a plant-defence, detoxification perspective revealed by RNA-seq analyses. Mol Ecol.

[75] Després L, David J-P, Gallet C (2007). The evolutionary ecology of insect resistance to plant chemicals. Trends Ecol Evol.

[76] Heckel DG. Insect detoxification and sequestration strategies. In: Annual plant reviews. John Wiley & Sons, Ltd; 2014. p. 77–114.

[77] Wang X-W, Liu S-S, Czosnek H, Ghanim M (2016). Functional genomics in the whitefly
*
Bemisia tabaci
*
species complex. Management of insect pests to agriculture: lessons learned from deciphering their genome, transcriptome and proteome.

[78] Horowitz AR, Ghanim M, Roditakis E, Nauen R, Ishaaya I (2020). Insecticide resistance and its management in *Bemisia tabaci* species. J Pest Sci.

[79] Xia J, Xu H, Yang Z, Pan H, Yang X, Guo Z (2019). Genome-wide analysis of carboxylesterases (COEs) in the whitefly, *Bemisia tabaci* (Gennadius). Int J Mol Sci.

[80] Oakeshott JG, van Papenrecht EA, Boyce TM, Healy MJ, Russell RJ (1993). Evolutionary genetics of *Drosophila* esterases. Genetica.

[81] Carnero Avilés L, Cerna Chávez E, Rodríguez Rodríguez JF, Beltrán Beache M, Ochoa Fuentes YM, Velarde Félix S (2021). Quantification of enzymes related to insecticide resistance in *Bemisia tabaci* from the state of Sinaloa. Rev Mex Cienc Agríc.

[82] Oakeshott JG, Claudianos C, Russell RJ, Robin GC (1999). Carboxyl/cholinesterases: a case study of the evolution of a successful multigene family. BioEssays.

[83] Feyereisen R (2006). Evolution of insect P450. Biochem Soc Trans.

[84] Karunker I, Benting J, Lueke B, Ponge T, Nauen R, Roditakis E (2008). Over-expression of cytochrome P450 *CYP6CM1* is associated with high resistance to imidacloprid in the B and Q biotypes of *Bemisia tabaci* (Hemiptera: Aleyrodidae). Insect Biochem Mol Biol.

[85] Zhou C, Cao Q, Li G, Ma D (2020). Role of several cytochrome P450s in the resistance and cross-resistance against imidacloprid and acetamiprid of *Bemisia tabaci* (Hemiptera: Aleyrodidae) MEAM1 cryptic species in Xinjiang. China Pestic Biochem Physiol.

[86] Snoeck S, Wybouw N, Van Leeuwen T, Dermauw W. Transcriptomic plasticity in the arthropod generalist *Tetranychus urticae* upon long-term acclimation to different host plants. G3 GenesGenomesGenetics. 2018;8:3865–79. 10.1534/g3.118.200585 PMC6288829 30333191

[87] Dermauw W, Van Leeuwen T, Feyereisen R (2020). Diversity and evolution of the P450 family in arthropods. Insect Biochem Mol Biol.

[88] Suiko M, Kurogi K, Hashiguchi T, Sakakibara Y, Liu M-C (2017). Updated perspectives on the cytosolic sulfotransferases (SULTs) and SULT-mediated sulfation. Biosci Biotechnol Biochem.

[89] Ung D, Nagar S (2007). Variable sulfation of dietary polyphenols by recombinant human sulfotransferase (SULT) 1A1 genetic variants and SULT1E1. Drug Metab Dispos.

[90] Dubaisi S, Fang H, Caruso JA, Gaedigk R, Vyhlidal CA, Kocarek TA (2020). Developmental expression of SULT1C4 transcript variants in human liver: implications for discordance between SULT1C4 mRNA and protein levels. Drug Metab Dispos.

[91] Aidlin Harari O, Santos-Garcia D, Musseri M, Moshitzky P, Patel M, Visendi P (2020). Molecular evolution of the glutathione S-transferase family in the *Bemisia tabaci* species complex. Genome Biol Evol.

[92] Enayati AA, Ranson H, Hemingway J (2005). Insect glutathione transferases and insecticide resistance. Insect Mol Biol.

[93] Shou-Min F (2012). Insect glutathione S-transferase: a review of comparative genomic studies and response to xenobiotics. Bull Insectol.

[94] Friedman R (2011). Genomic organization of the glutathione S-transferase family in insects. Mol Phylogenet Evol.

[95] Yang X, He C, Xie W, Liu Y, Xia J, Yang Z (2016). Glutathione S-transferases are involved in thiamethoxam resistance in the field whitefly *Bemisia tabaci* Q (Hemiptera: Aleyrodidae). Pestic Biochem Physiol.

[96] Elbaz M, Halon E, Malka O, Malitsky S, Blum E, Aharoni A (2012). Asymmetric adaptation to indolic and aliphatic glucosinolates in the B and Q sibling species of *Bemisia tabaci* (Hemiptera: Aleyrodidae). Mol Ecol.

[97] Eakteiman G, Moses-Koch R, Moshitzky P, Mestre-Rincon N, Vassão DG, Luck K (2018). Targeting detoxification genes by phloem-mediated RNAi: a new approach for controlling phloem-feeding insect pests. Insect Biochem Mol Biol.

[98] Ranson H, Hemingway J, Sies H, Packer L (2005). Mosquito glutathione transferases. Methods in Enzymology.

[99] Ahn S-J, Vogel H, Heckel DG (2012). Comparative analysis of the UDP-glycosyltransferase multigene family in insects. Insect Biochem Mol Biol.

[100] Guo L, Xie W, Yang Z, Xu J, Zhang Y (2020). Genome-wide identification and expression analysis of UDP-glucuronosyltransferases in the whitefly *Bemisia tabaci* (Gennadius) (Hemiptera: Aleyrodidae). Int J Mol Sci.

[101] Pym A, Singh KS, Nordgren Å, Davies TGE, Zimmer CT, Elias J (2019). Host plant adaptation in the polyphagous whitefly, *Trialeurodes vaporariorum*, is associated with transcriptional plasticity and altered sensitivity to insecticides. BMC Genomics.

[102] Xiao H-Y, Chen D-L, Lu T-T, Yao Y-J, Liu N-Y (2022). The UDP-glycosyltransferase gene family in *Achelura yunnanensis* (Lepidoptera: Zygaenidae): identification, phylogeny, and diverse expression patterns. Diversity.

[103] Dean M, Hamon Y, Chimini G (2001). The human ATP-binding cassette (ABC) transporter superfamily. J Lipid Res.

[104] He C, Liang J, Liu S, Wang S, Wu Q, Xie W (2019). Changes in the expression of four ABC transporter genes in response to imidacloprid in *Bemisia tabaci* Q (Hemiptera: Aleyrodidae). Pestic Biochem Physiol.

[105] Tian L, Song T, He R, Zeng Y, Xie W, Wu Q (2017). Genome-wide analysis of ATP-binding cassette (ABC) transporters in the sweetpotato whitefly, *Bemisia tabaci*. BMC Genomics.

[106] Ashford DA, Smith WA, Douglas AE (2000). Living on a high sugar diet: the fate of sucrose ingested by a phloem-feeding insect, the pea aphid *Acyrthosiphon pisum*. J Insect Physiol.

[107] Cristofoletti PT, Ribeiro AF, Deraison C, Rahbé Y, Terra WR (2003). Midgut adaptation and digestive enzyme distribution in a phloem feeding insect, the pea aphid *Acyrthosiphon pisum*. J Insect Physiol.

[108] Jing X, White TA, Luan J, Jiao C, Fei Z, Douglas AE (2016). Evolutionary conservation of candidate osmoregulation genes in plant phloem sap-feeding insects. Insect Mol Biol.

[109] Nakai H, Okuyama M, Kim YM, Saburi W, Wongchawalit J, Mori H (2005). Molecular analysis of α-glucosidase belonging to GH-family 31. Biol - Sect Cell Mol Biol.

[110] Chiba S (1997). Molecular mechanism in α-glucosidase and glucoamylase. Biosci Biotechnol Biochem.

[111] Douglas AE (2006). Phloem-sap feeding by animals: problems and solutions. J Exp Bot.

[112] Malka O, Feldmesser E, van Brunschot S, Santos-Garcia D, Han WH, Seal S (2021). The molecular mechanisms that determine different degrees of polyphagy in the *Bemisia tabaci* species complex. Evol Appl.

[113] Von Heljne G (1998). Life and death of a signal peptide. Nature.

[114] Cohen AC, Hendrix DL (1994). Demonstration and preliminary characterization of α-amylase in the sweetpotato whitefly, *Bemisia tabaci* (Aleyrodidae: Homoptera). Comp Biochem Physiol Part B Comp Biochem.

[115] Biasini M, Bienert S, Waterhouse A, Arnold K, Studer G, Schmidt T (2014). SWISS-MODEL: modelling protein tertiary and quaternary structure using evolutionary information. Nucleic Acids Res.

[116] Smith MD, Wertheim JO, Weaver S, Murrell B, Scheffler K, Kosakovsky Pond SL (2015). Less is more: an adaptive branch-site random effects model for efficient detection of episodic diversifying selection. Mol Biol Evol.

[117] Kosakovsky Pond SL, Frost SDW (2005). Not so different after all: a comparison of methods for detecting amino acid sites under selection. Mol Biol Evol.

[118] Gabriško M (2013). Evolutionary history of eukaryotic α-glucosidases from the α-amylase family. J Mol Evol.

[119] Hunt BG, Ometto L, Wurm Y, Shoemaker DW, Yi SV, Keller L (2011). Relaxed selection is a precursor to the evolution of phenotypic plasticity. Proc Natl Acad Sci U S A.

[120] Lynch M, Conery JS (2000). The evolutionary fate and consequences of duplicate genes. Science.

[121] Fiallo-Olivé E, Pan L-L, Liu S-S, Navas-Castillo J (2020). Transmission of begomoviruses and other whitefly-borne viruses: dependence on the vector species. Phytopathology.

[122] Fiallo-Olivé E, Lett JM, Martin DP, Roumagnac P, Varsani A, Zerbini FM (2021). ICTV virus taxonomy profile: Geminiviridae 2021. J Gen Virol.

[123] Kang D, Liu G, Lundström A, Gelius E, Steiner H (1998). A peptidoglycan recognition protein in innate immunity conserved from insects to humans. Proc Natl Acad Sci U S A.

[124] Jiang L, Liu W, Guo H, Dang Y, Cheng T, Yang W (2019). Distinct functions of *Bombyx mori* peptidoglycan recognition protein 2 in immune responses to bacteria and viruses. Front Immunol.

[125] Tsai CW, McGraw EA, Ammar E-D, Dietzgen RG, Hogenhout SA (2008). *Drosophila melanogaster* mounts a unique immune response to the Rhabdovirus *Sigma virus*. Appl Environ Microbiol.

[126] Wang J, Song X, Wang M (2018). Peptidoglycan recognition proteins in hematophagous arthropods. Dev Comp Immunol.

[127] Mellroth P, Karlsson J, Steiner H (2003). A scavenger function for a *Drosophila* peptidoglycan recognition protein. J Biol Chem.

[128] Christophides GK, Zdobnov E, Barillas-Mury C, Birney E, Blandin S, Blass C (2002). Immunity-related genes and gene families in *Anopheles gambiae*. Science.

[129] International Glossina Genome Initiative, Attardo GM, Abila PP, Auma JE, Baumann AA, Benoit JB, et al. Genome sequence of the Tsetse Fly (*Glossina morsitans*): vector of African Trypanosomiasis. Science. 2014;344:380–6. 10.1126/science.1249656 PMC4077534 24763584

[130] Wang S, Beerntsen BT (2015). Functional implications of the peptidoglycan recognition proteins in the immunity of the yellow fever mosquito, *Aedes aegypti*. Insect Mol Biol.

[131] Wang ZZ, Shi M, Huang YC, Wang XW, Stanley D, Chen XX. A peptidoglycan recognition protein acts in whitefly (*Bemisia tabaci*) immunity and involves in *Begomovirus* acquisition. Sci Rep. 2016;6. 10.1038/srep37806 PMC5124967 27892529

[132] Lim J-H, Kim M-S, Kim H-E, Yano T, Oshima Y, Aggarwal K (2006). Structural basis for preferential recognition of diaminopimelic acid-type peptidoglycan by a subset of peptidoglycan recognition proteins. J Biol Chem.

[133] Wang X-R, Wang C, Ban F-X, Zhu D-T, Liu S-S, Wang X-W (2019). Genome-wide identification and characterization of HSP gene superfamily in whitefly (*Bemisia tabaci*) and expression profiling analysis under temperature stress. Insect Sci.

[134] Zheng H-Y, Qin P-H, Yang K, Liu T-X, Zhang Y-J, Chu D (2022). Genome-wide identification and analysis of the heat-shock protein gene superfamily in *Bemisia tabaci* and expression pattern analysis under heat shock. Insects.

[135] Bai J, Wang Y-C, Liu Y-C, Chang Y-W, Liu X-N, Gong W-R (2021). Isolation of two new genes encoding heat shock protein 70 in *Bemisia tabaci* and analysis during thermal stress. Int J Biol Macromol.

[136] Bai J, Liu X-N, Lu M-X, Du Y-Z (2021). Transcriptional profiling of MED *Bemisia tabaci* exposed to thermal stress and verification of HSP70 expression. Entomol Res.

[137] Gotz M, Popovski S, Kollenberg M, Gorovits R, Brown JK, Cicero JM (2012). Implication of *Bemisia tabaci* heat shock protein 70 in begomovirus-whitefly interactions. J Virol.

[138] Kanakala S, Kontsedalov S, Lebedev G, Ghanim M. Plant-mediated silencing of the whitefly *Bemisia tabaci* cyclophilin B and heat shock protein 70 impairs insect development and virus transmission. Front Physiol. 2019;0:557. 10.3389/fphys.2019.00557 PMC6517521 31133883

[139] Li Z, Srivastava P. Heat-shock proteins. Curr Protoc Immunol. 2004;58:A.1T.1-A.1T.6. 10.1002/0471142735.ima01ts58 18432918

[140] Mahadav A, Kontsedalov S, Czosnek H, Ghanim M (2009). Thermotolerance and gene expression following heat stress in the whitefly *Bemisia tabaci* B and Q biotypes. Insect Biochem Mol Biol.

[141] Jiang R, Qi L-D, Du Y-Z, Li Y-X (2017). Thermotolerance and heat-shock protein gene expression patterns in *Bemisia tabaci* (Hemiptera: Aleyrodidae) Mediterranean in relation to developmental stage. J Econ Entomol.

[142] Xia W-Q, Liang Y, Chi Y, Pan L-L, Zhao J, Liu S-S (2018). Intracellular trafficking of begomoviruses in the midgut cells of their insect vector. PLOS Pathog.

[143] Uchibori M, Hirata A, Suzuki M, Ugaki M (2013). *Tomato yellow leaf curl virus* accumulates in vesicle-like structures in descending and ascending midgut epithelial cells of the vector whitefly, *Bemisia tabaci*, but not in those of nonvector whitefly *Trialeurodes vaporariorum*. J Gen Plant Pathol.

[144] Zhao J, Chi Y, Zhang XJ, Wang XW, Liu SS (2019). Implication of whitefly vesicle associated membrane protein-associated protein B in the transmission of *Tomato yellow leaf curl virus*. Virology.

[145] Sánchez-Campos S, Navas-Castillo J, Camero R, Soria C, Díaz JA, Moriones E (1999). Displacement of *Tomato yellow leaf curl virus* (TYLCV)-Sr by TYLCV-Is in tomato epidemics in Spain. Phytopathology.

[146] Rana VS, Popli S, Saurav GK, Raina HS, Chaubey R, Ramamurthy VV (2016). A *Bemisia tabaci* midgut protein interacts with begomoviruses and plays a role in virus transmission. Cell Microbiol.

[147] Wang P, Heitman J (2005). The cyclophilins. Genome Biol.

[148] Kumari S, Roy S, Singh P, Singla-Pareek SL, Pareek A (2013). Cyclophilins: proteins in search of function. Plant Signal Behav.

[149] Tamborindeguy C, Bereman MS, DeBlasio S, Igwe D, Smith DM, White F (2013). Genomic and proteomic analysis of *Schizaphis graminum* reveals cyclophilin proteins are involved in the transmission of *Cereal yellow dwarf virus*. PLoS ONE.

[150] Kanakala S, Ghanim M (2016). Implication of the whitefly *Bemisia tabaci* cyclophilin B protein in the transmission of *Tomato yellow leaf curl virus*. Front Plant Sci.

[151] Czosnek H, Ghanim M, Ghanim M (2002). The circulative pathway of begomoviruses in the whitefly vector *Bemisia tabaci*— insights from studies with *Tomato yellow leaf curl virus*. Ann Appl Biol.

[152] Luan J-B, Chen W, Hasegawa DK, Simmons AM, Wintermantel WM, Ling K-S (2015). Metabolic coevolution in the bacterial symbiosis of whiteflies and related plant sap-feeding insects. Genome Biol Evol.

[153] Wood DE, Salzberg SL (2014). Kraken: ultrafast metagenomic sequence classification using exact alignments. Genome Biol.

[154] Wood DE, Lu J, Langmead B (2019). Improved metagenomic analysis with Kraken 2. Genome Biol.

[155] Li H (2018). Minimap2: pairwise alignment for nucleotide sequences. Bioinformatics.

[156] Ruan J, Li H (2020). Fast and accurate long-read assembly with wtdbg2. Nat Methods.

[157] Pryszcz LP, Gabaldón T (2016). Redundans: an assembly pipeline for highly heterozygous genomes. Nucleic Acids Res.

[158] English AC, Richards S, Han Y, Wang M, Vee V, Qu J (2012). Mind the gap: upgrading genomes with Pacific Biosciences RS long-read sequencing technology. PLoS ONE.

[159] Kumar S, Jones M, Koutsovoulos G, Clarke M, Blaxter M. Blobology: exploring raw genome data for contaminants, symbionts and parasites using taxon-annotated GC-coverage plots. Front Genet. 2013;4. 10.3389/fgene.2013.00237 PMC3843372 24348509

[160] Laetsch DR, Blaxter ML. BlobTools: interrogation of genome assemblies. F1000Research. 2017;6:1287.

[161] Curwen V, Eyras E, Andrews TD, Clarke L, Mongin E, Searle SMJ (2004). The Ensembl automatic gene annotation system. Genome Res.

[162] Campbell LI, van Brunschot SL, Nwezeobi J. Additional file 6: Ensembl genebuild annotation of *B. argentifolii *and *B. tabaci s.s. *genomes. Figshare [dataset]. 2023. 10.6084/m9.figshare.23666832.

[163] Zdobnov EM, Apweiler R (2001). InterProScan-an integration platform for the signature-recognition methods in InterPro. Bioinformatics.

[164] Ye J, Fang L, Zheng H, Zhang Y, Chen J, Zhang Z, et al. WEGO: a web tool for plotting GO annotations. Nucleic Acids Res. 2006;34 suppl_2:W293–7. 10.1093/nar/gkl031 PMC1538768 16845012

[165] Ye J, Zhang Y, Cui H, Liu J, Wu Y, Cheng Y, et al. WEGO 2.0: a web tool for analyzing and plotting GO annotations, 2018 update. Nucleic Acids Res. 2018;46:W71–5.166. Katoh K, Standley DM. MAFFT multiple sequence alignment software version 7: improvements in performance and usability. Mol Biol Evol. 2013;30:772–80.

[166] Katoh K, Standley DM (2013). MAFFT multiple sequence alignment software version 7: improvements in performance and usability. Mol Biol Evol.

[167] Talavera G, Castresana J (2007). Improvement of phylogenies after removing divergent and ambiguously aligned blocks from protein sequence alignments. Syst Biol.

[168] Darriba D, Taboada GL, Doallo R, Posada D (2011). ProtTest 3: fast selection of best-fit models of protein evolution. Bioinformatics.

[169] Stamatakis A (2014). RAxML version 8: a tool for phylogenetic analysis and post-analysis of large phylogenies. Bioinformatics.

[170] Huelsenbeck JP, Ronquist F (2001). MRBAYES: Bayesian inference of phylogenetic trees. Bioinformatics.

[171] Ronquist F, Huelsenbeck JP (2003). MrBayes 3: Bayesian phylogenetic inference under mixed models. Bioinformatics.

[172] Aubry S, Kelly S, Kümpers BMC, Smith-Unna RD, Hibberd JM (2014). Deep evolutionary comparison of gene expression identifies parallel recruitment of trans-factors in two independent origins of C4 photosynthesis. PLOS Genet.

[173] Altschul SF, Gish W, Miller W, Myers EW, Lipman DJ (1990). Basic local alignment search tool. J Mol Biol.

[174] Morgulis A, Coulouris G, Raytselis Y, Madden TL, Agarwala R, Schäffer AA (2008). Database indexing for production MegaBLAST searches. Bioinformatics.

[175] Bailly-Bechet M, Haudry A, Lerat E (2014). “One code to find them all”: a Perl tool to conveniently parse RepeatMasker output files. Mob DNA.

[176] Campbell LI, van Brunschot SL, Nwezeobi J. Additional file 7: Endosymbiont assemblies and annotations of *Candidatus* Portiera aleyrodidarum from six populations of *Bemisia tabaci s.l.* Figshare [dataset]. 2023. 10.6084/m9.figshare.23666844.

[177] Kolmogorov M, Yuan J, Lin Y, Pevzner PA (2019). Assembly of long, error-prone reads using repeat graphs. Nat Biotechnol.

[178] Seemann T (2014). Prokka: rapid prokaryotic genome annotation. Bioinformatics.

[179] Rutherford K, Parkhill J, Crook J, Horsnell T, Rice P, Rajandream M-A (2000). Artemis: sequence visualization and annotation. Bioinformatics.

[180] Guy L, Roat Kultima J, Andersson SG (2010). genoPlotR: comparative gene and genome visualization in R. Bioinformatics.

[181] Rodriguez-R LM, Konstantinidis KT. The enveomics collection: a toolbox for specialized analyses of microbial genomes and metagenomes. PeerJ Preprints; 2016.

[182] Edgar RC (2010). Search and clustering orders of magnitude faster than BLAST. Bioinformatics.

[183] R Core Team. A language and environment for statistical computing. 2013; 275-86.

