# Supplementary material for: Comparative evolutionary analyses of eight whitefly Bemisia tabaci sensu lato genomes: cryptic species, agricultural pests and plant-virus vectors
Source: BMC Genomics. 2023 Jul 19;24:408. doi: 10.1186/s12864-023-09474-3 (PMC10357772; doi:10.1186/s12864-023-09474-3)
Supplement: Supplementary file 2 — Additional file 2. Supplementary figures S1-S17. [file 12864_2023_9474_MOESM2_ESM.docx]

**Additional file 2: Supplementary figures S1-S17**

Comparative evolutionary analyses of eight whitefly *Bemisia tabaci sensu lato* genomes: cryptic species, agricultural pests and plant-virus vectors

Lahcen I. Campbell, Joachim Nwezeobi, Sharon L. van Brunschot, Tadeo Kaweesi, Susan E. Seal, Rekha Swamy, Annet Namuddu, Gareth L. Maslen, Habibu Mugerwa, Irina M. Armean, Leanne Haggerty, Fergal J. Martin, Osnat Malka, Diego Santos-Garcia, Ksenia Juravel, Shai Morin, Michael E. Stephens, Paul Visendi Muhindira, Paul J. Kersey, M. N. Maruthi, Christopher A. Omongo, Jesús Navas-Castillo, Elvira Fiallo-Olivé, Ibrahim Umar Mohammed, Hua-Ling Wang, Joseph Onyeka, Titus Alicai, John Colvin.

Correspondence to: [lcampbell@ebi.ac.uk](mailto:lcampbell@ebi.ac.uk); [jn11@sanger.ac.uk](mailto:jn11@sanger.ac.uk)

**Table of contents**

[Fig. S1: Genomic PacBio data contamination filtration 2](#_heading=h.30j0zll)

[Fig. S2: Draft assembly assessment and taxonomic assignment in Asia II-5 3](#_heading=h.1fob9te)

[Fig. S3: Draft assembly assessment and taxonomic assignment in SSA1-SG1-Ng 4](#_heading=h.3znysh7)

[Fig. S4: Draft assembly assessment and taxonomic assignment in SSA1-SG-Ug 5](#_heading=h.2et92p0)

[Fig. S5: Draft assembly assessment and taxonomic assignment in SSA2-Ng 6](#_heading=h.tyjcwt)

[Fig. S6: Draft assembly assessment and taxonomic assignment in SSA3-Ng 7](#_heading=h.3dy6vkm)

[Fig. S7: Draft assembly assessment and taxonomic assignment in Uganda-1 8](#_heading=h.1t3h5sf)

[Fig. S8: Mitochondrial phylogenomic analysis of whitefly and related hemiptera. 9](#_heading=h.4d34og8)

[Fig. S9: Inferred coding capabilities of Candidatus Portiera aleyrodidarum from different Bemisia hosts 10](#_heading=h.2s8eyo1)

[Fig. S10: Phylogenetic analysis of carboxylesterase proteins using maximum likelihood (ML) 11](#_heading=h.17dp8vu)

[Fig. S11: Phylogenetic analysis of cytochrome P450 proteins using maximum likelihood (ML) 13](#_heading=h.26in1rg)

[Fig. S12: Phylogenetic analysis of sulfotransferases using maximum likelihood (ML) 15](#_heading=h.lnxbz9)

[Fig. S13: Phylogenetic analysis of GSTs using maximum likelihood (ML) 17](#_heading=h.1ksv4uv)

[Fig. S14: Phylogenetic analysis of UGTs using maximum likelihood (ML) 19](#_heading=h.2jxsxqh)

[Fig. S15: Phylogenetic analysis of ABC transporter proteins using maximum likelihood (ML) 21](#_heading=h.z337ya)

[Fig. S16: Omega plot showing ω distribution under the RELAX alternative model of SSA1-SG1-Ug α-glucosidase (GH13) genes 23](#_heading=h.1y810tw)

[Fig. S17: Omega plot showing ω distribution under the RELAX alternative model with SSA1-SG1-Ng α-glucosidase (GH13) genes 24](#_heading=h.2xcytpi)

##


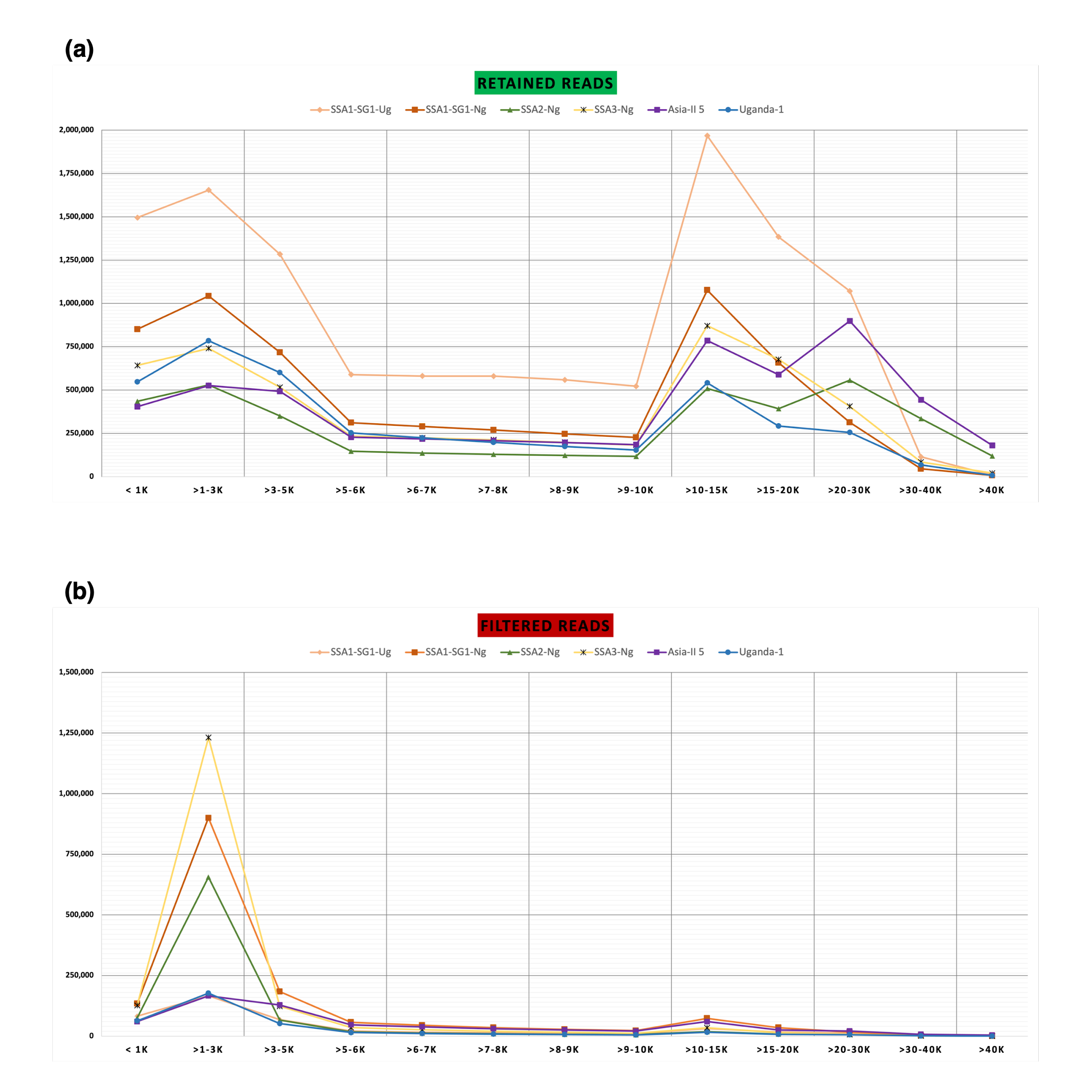


## Fig. S1: Genomic PacBio data contamination filtration

Summary of read filtration using Kraken2 across *Bemisia tabaci s.l.* genomic PacBio data generated as part of this study. Analysis performed with respect to putative contaminant sources formatted into Kraken2 databases. (a) Retained putative *Bemisia* reads after screening protocol; then provided as input for nuclear genomic assembly. (b) Non-*Bemisia*/contaminant reads identified by Kraken2 and removed from downstream analysis. X-axis: PacBio reads binned by length, <1,000 bp (1k) - >40,000 bp (40 k). Y-axis: total read count per bin. Major gridlines (n=50,000); Minor gridlines (n=20,000).


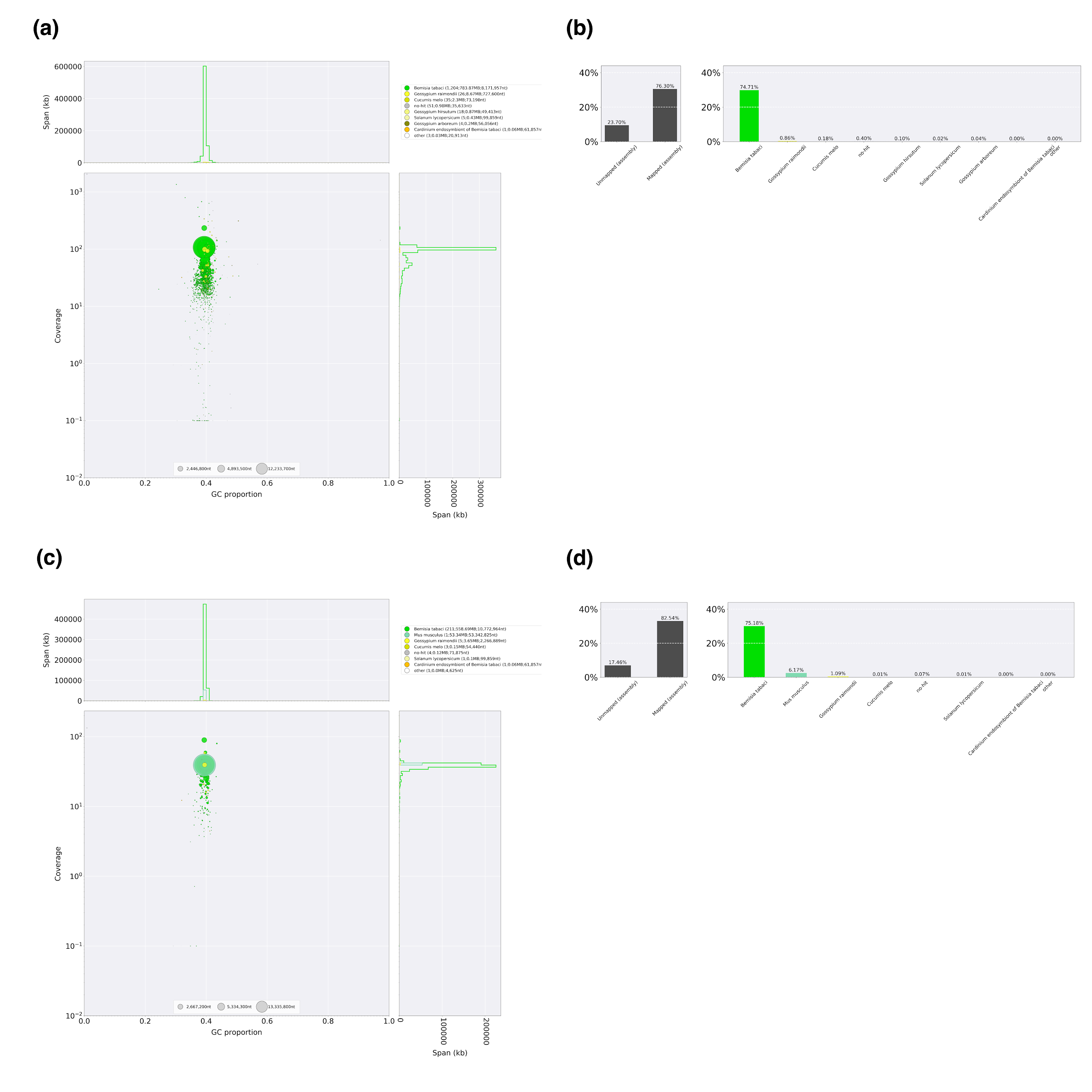


## Fig. S2: Draft assembly assessment and taxonomic assignment in Asia II-5

A graphical representation or ‘blobplot’ of the initial unaltered de novo draft assembly (a) and its associated assembly read mapping coverage with taxonomic read assignment (b); compared to the finalized, taxonomically filtered assembly (c) and its associated assembly read mapping coverage (d). Individual scaffolds plotted independently (circular blobs) can be seen in (a,c) where blob size scales with scaffold length; scaffold read mapping coverage (y-axis) versus scaffold GC% (x-axis). Taxonomic assignments are uniquely colored for clarity, while taxonomy color legend remains constant for all assemblies produced in this study.


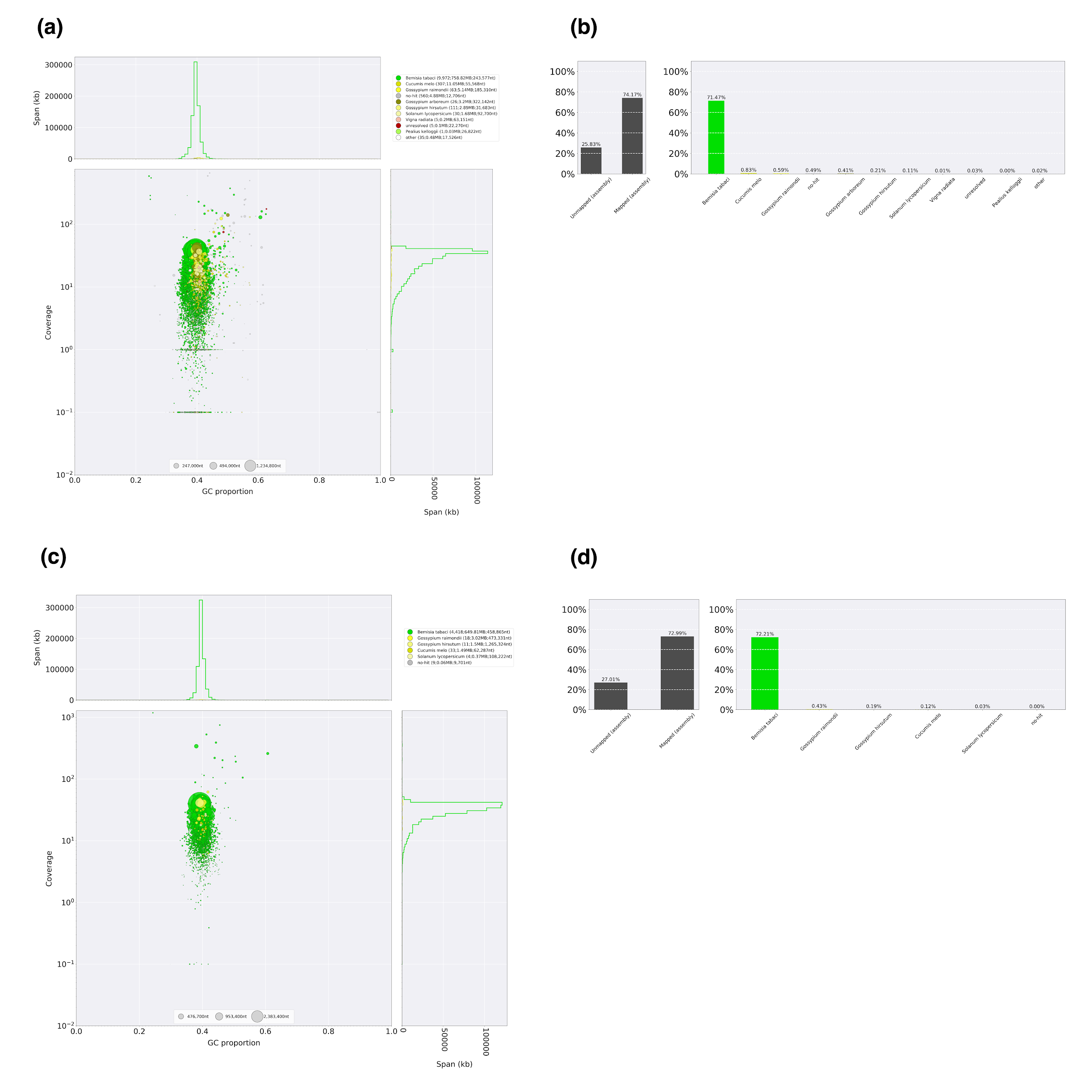


## Fig. S3: Draft assembly assessment and taxonomic assignment in SSA1-SG1-Ng

A graphical representation or ‘blobplot’ of the initial unaltered de novo draft assembly (a) and its associated assembly read mapping coverage with taxonomic read assignment (b); compared to the finalized, taxonomically filtered assembly (c) and its associated assembly read mapping coverage (d). Individual scaffolds plotted independently (circular blobs) can be seen in (a,c) where blob size scales with scaffold length; scaffold read mapping coverage (y-axis) versus scaffold GC% (x-axis). Taxonomic assignments are uniquely colored for clarity, while taxonomy color legend remains constant for all assemblies produced in this study.


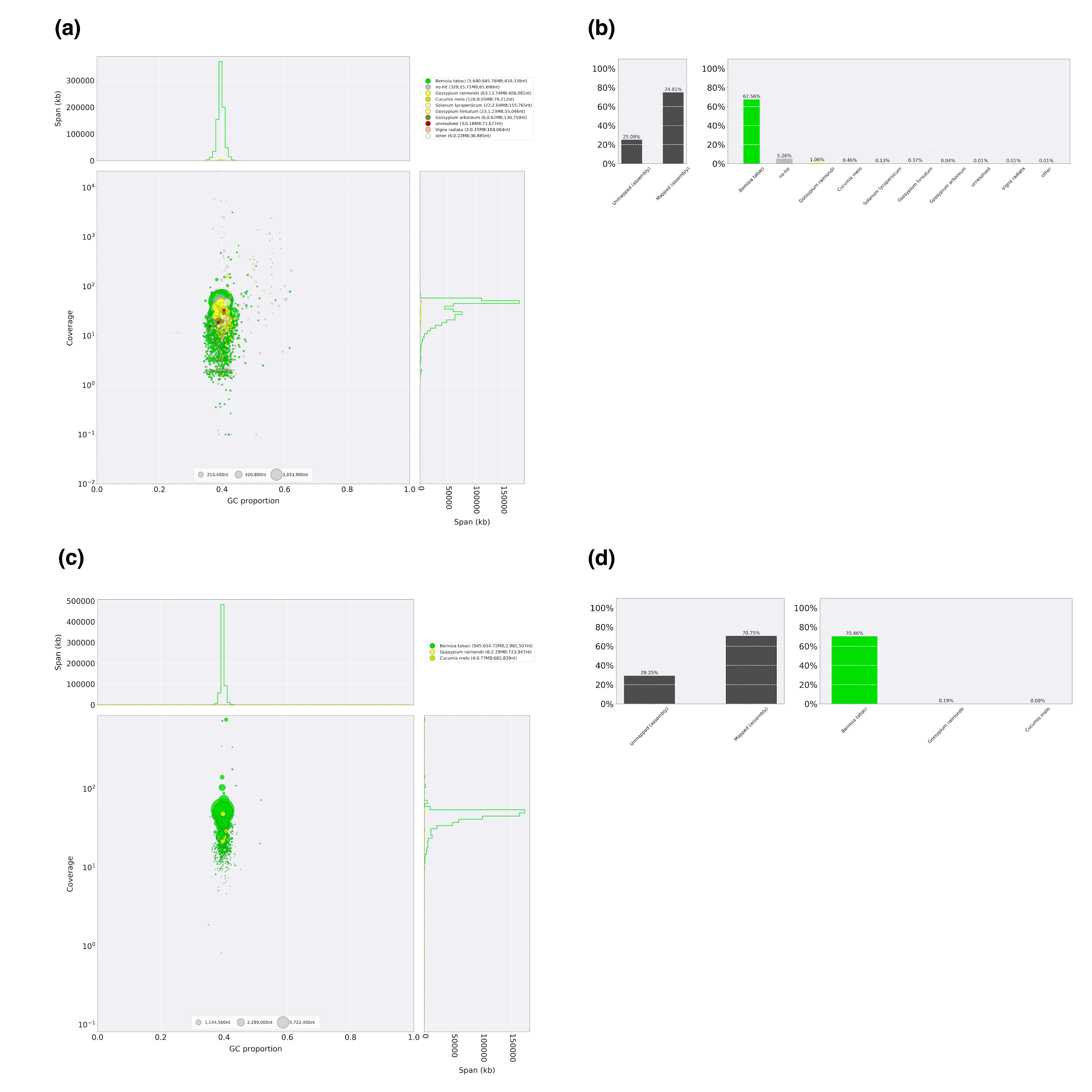


## Fig. S4: Draft assembly assessment and taxonomic assignment in SSA1-SG-Ug

A graphical representation or ‘blobplot’ of the initial unaltered de novo draft assembly (a) and its associated assembly read mapping coverage with taxonomic read assignment (b); compared to the finalized, taxonomically filtered assembly (c) and its associated assembly read mapping coverage (d). Individual scaffolds plotted independently (circular blobs) can be seen in (a,c) where blob size scales with scaffold length; scaffold read mapping coverage (y-axis) versus scaffold GC% (x-axis). Taxonomic assignments are uniquely colored for clarity, while taxonomy color legend remains constant for all assemblies produced in this study.


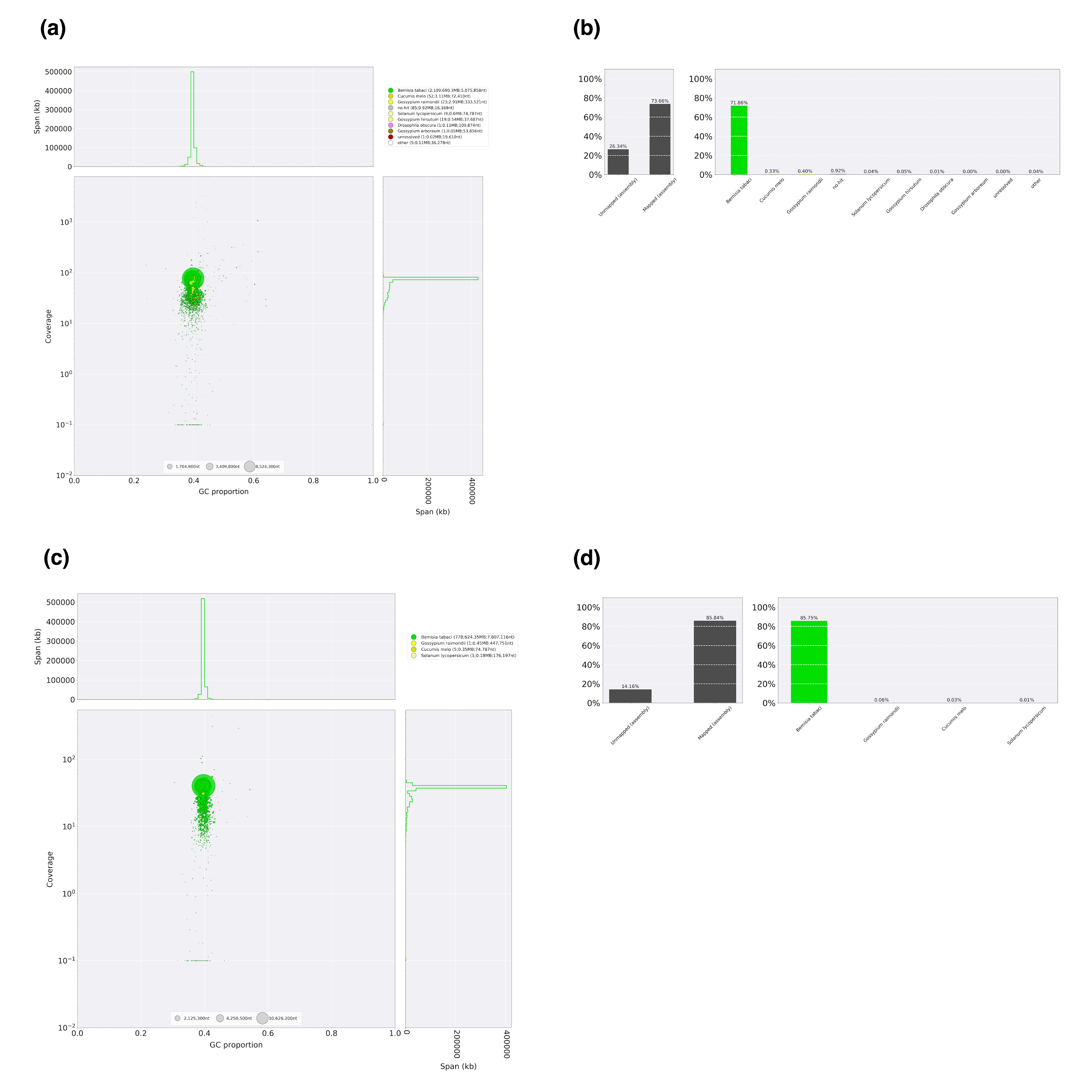


## Fig. S5: Draft assembly assessment and taxonomic assignment in SSA2-Ng

A graphical representation or ‘blobplot’ of the initial unaltered de novo draft assembly (a) and its associated assembly read mapping coverage with taxonomic read assignment (b); compared to the finalized, taxonomically filtered assembly (c) and its associated assembly read mapping coverage (d). Individual scaffolds plotted independently (circular blobs) can be seen in (a,c) where blob size scales with scaffold length; scaffold read mapping coverage (y-axis) versus scaffold GC% (x-axis). Taxonomic assignments are uniquely colored for clarity, while taxonomy color legend remains constant for all assemblies produced in this study.


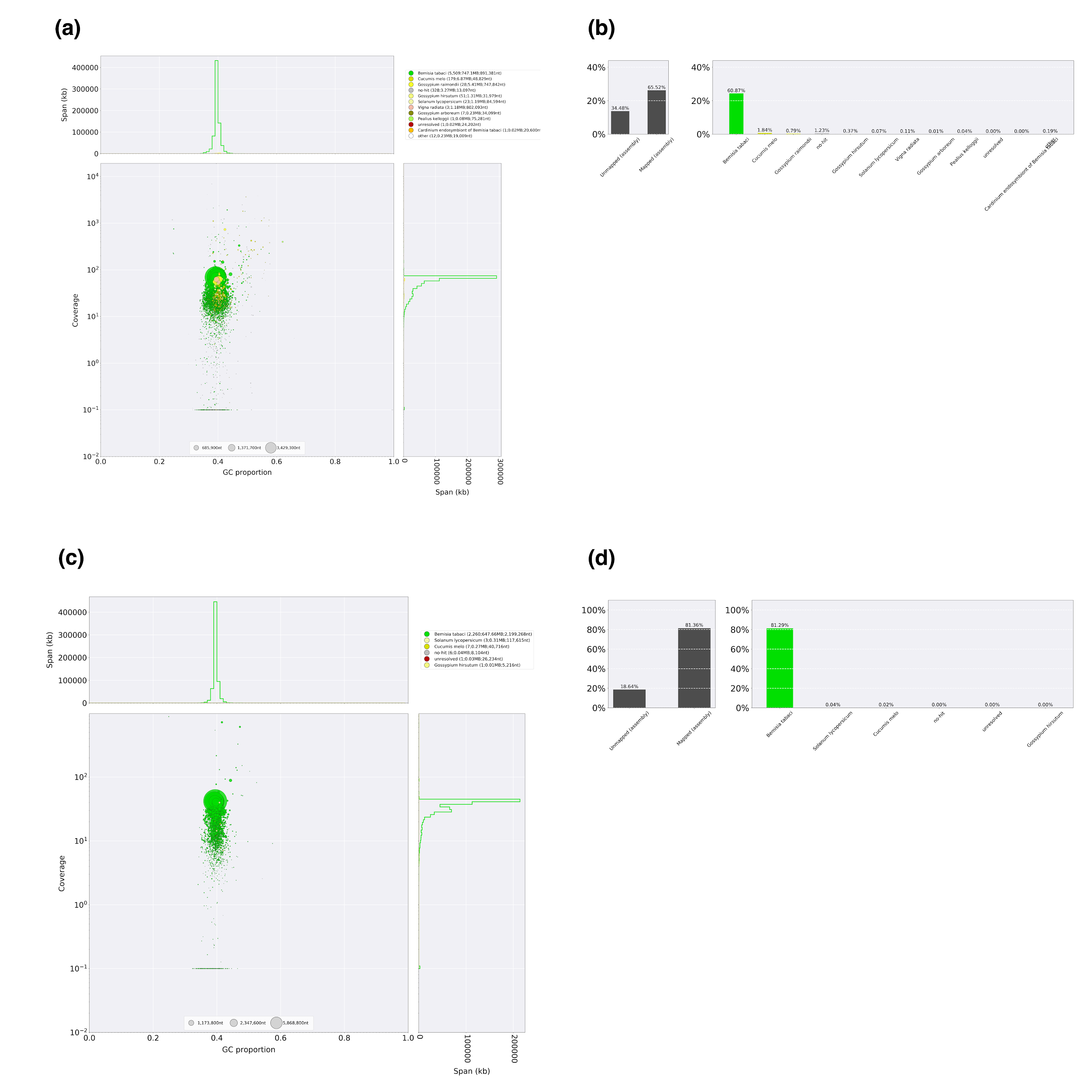


## Fig. S6: Draft assembly assessment and taxonomic assignment in SSA3-Ng

A graphical representation or ‘blobplot’ of the initial unaltered de novo draft assembly (a) and its associated assembly read mapping coverage with taxonomic read assignment (b); compared to the finalized, taxonomically filtered assembly (c) and its associated assembly read mapping coverage (d). Individual scaffolds plotted independently (circular blobs) can be seen in (a,c) where blob size scales with scaffold length; scaffold read mapping coverage (y-axis) versus scaffold GC% (x-axis). Taxonomic assignments are uniquely colored for clarity, while taxonomy color legend remains constant for all assemblies produced in this study.


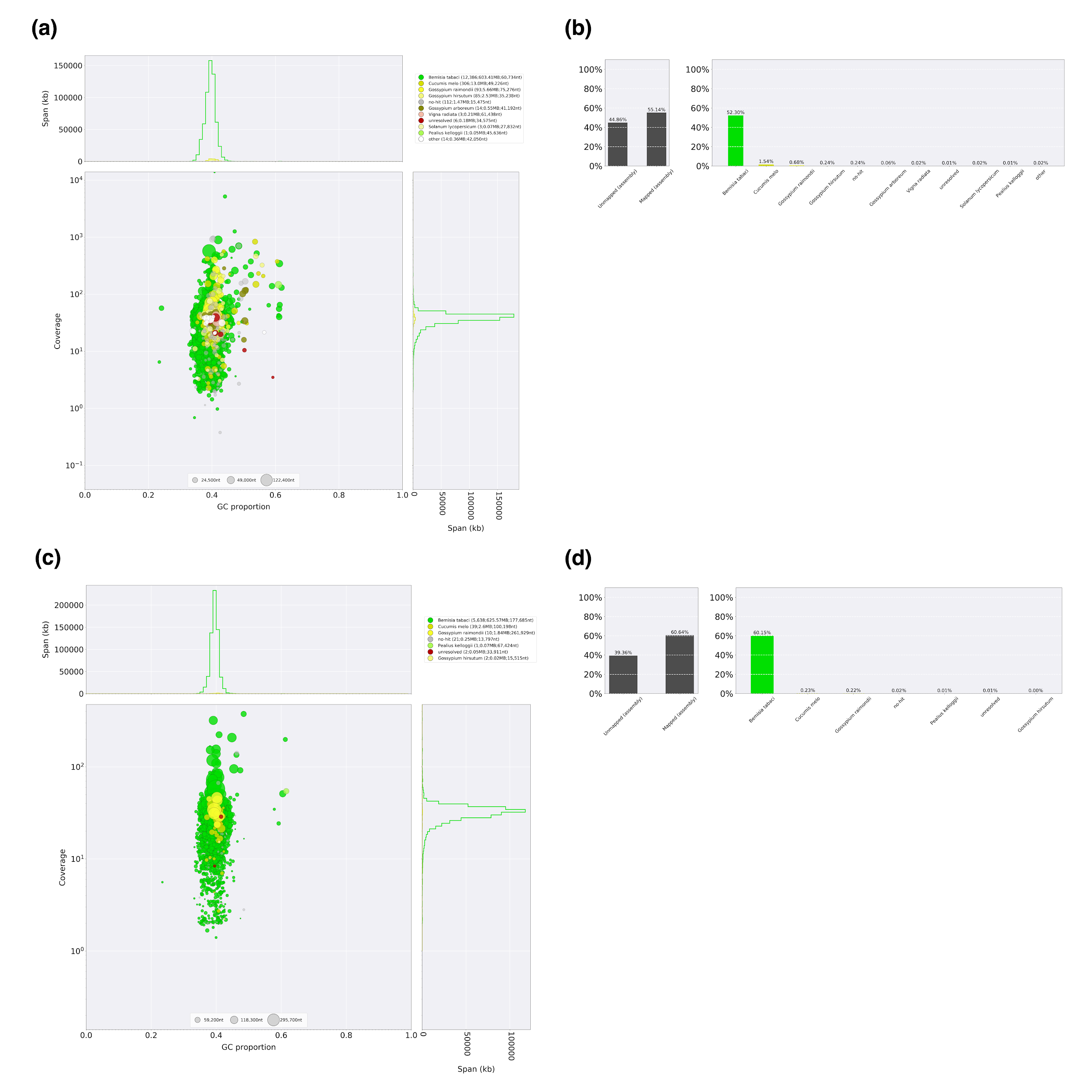


## Fig. S7: Draft assembly assessment and taxonomic assignment in Uganda-1

A graphical representation or ‘blobplot’ of the initial unaltered de novo draft assembly (a) and its associated assembly read mapping coverage with taxonomic read assignment (b); compared to the finalized, taxonomically filtered assembly (c) and its associated assembly read mapping coverage (d). Individual scaffolds plotted independently (circular blobs) can be seen in (a,c) where blob size scales with scaffold length; scaffold read mapping coverage (y-axis) versus scaffold GC% (x-axis). Taxonomic assignments are uniquely colored for clarity, while taxonomy color legend remains constant for all assemblies produced in this study.


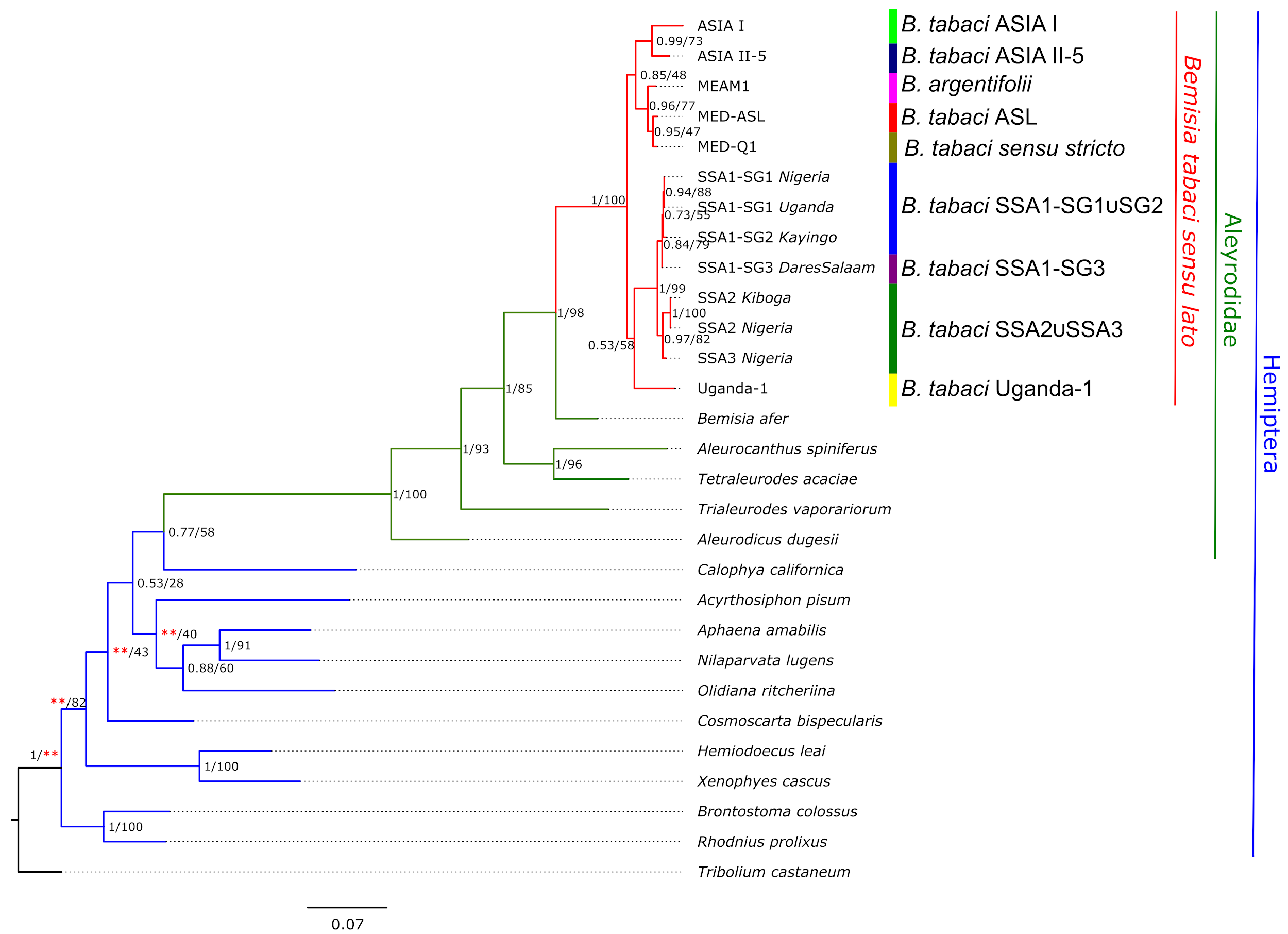


## Fig. S8: Mitochondrial phylogenomic analysis of whitefly and related hemiptera.

Analysis of 11 mitochondrial genes (nucleotide sequences) from 18 whitefly populations, 10 Hemiptera and one Coleoptera species. The three populations of *Bemisia tabaci* SSA1-SG1 Nigeria, SSA1-SG1 Uganda and SSA1-SG2 Kayingo were grouped together as the same biological species called *B. tabaci* SSA1-SG1∪SG2. Also, the three populations SSA2 Kiboga, SSA2 Nigeria and SSA3 Nigeria were grouped together as the biological species, *B. tabaci* SSA2∪SSA3. The tree shown above was constructed using RAxML and the MrBayes. The nodal support was generated by MrBayes’ posterior probability and RAxML bootstrap. The wildcard (**) shown in red is used to indicate the node where the RAxML tree does not provide a support value.

##
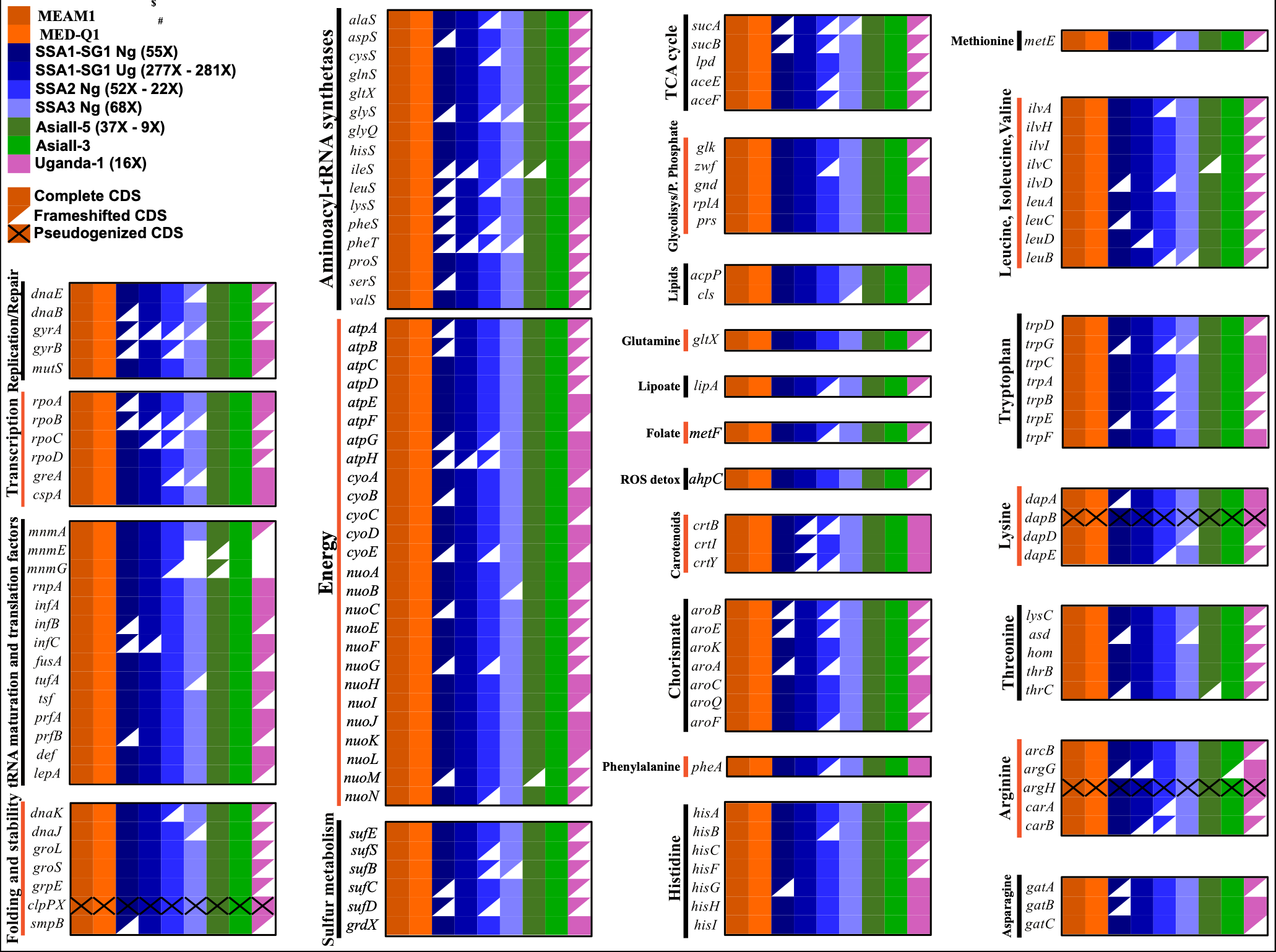
Fig. S9: Inferred coding capabilities of *Candidatus* Portiera aleyrodidarum from different *Bemisia* hosts

Presence and status of Coding Sequences (CDS) for the most relevant cell functions and metabolic capabilities of different *Portiera* strains from *Bemisia tabaci s.s.*, *B. tabaci s.l.* and *B. argentifolii*. Each gene was plotted only once, although it was involved in two or more categories. Filled squares indicate complete CDS, triangles represent putative sequencing-related artifactual frame-shifted genes, crossed squares represent already known pseudogenization events in *Portiera* from *Bemisia tabaci s.s*., *B. tabaci s.l.*, and *B. argentifolii*.

##
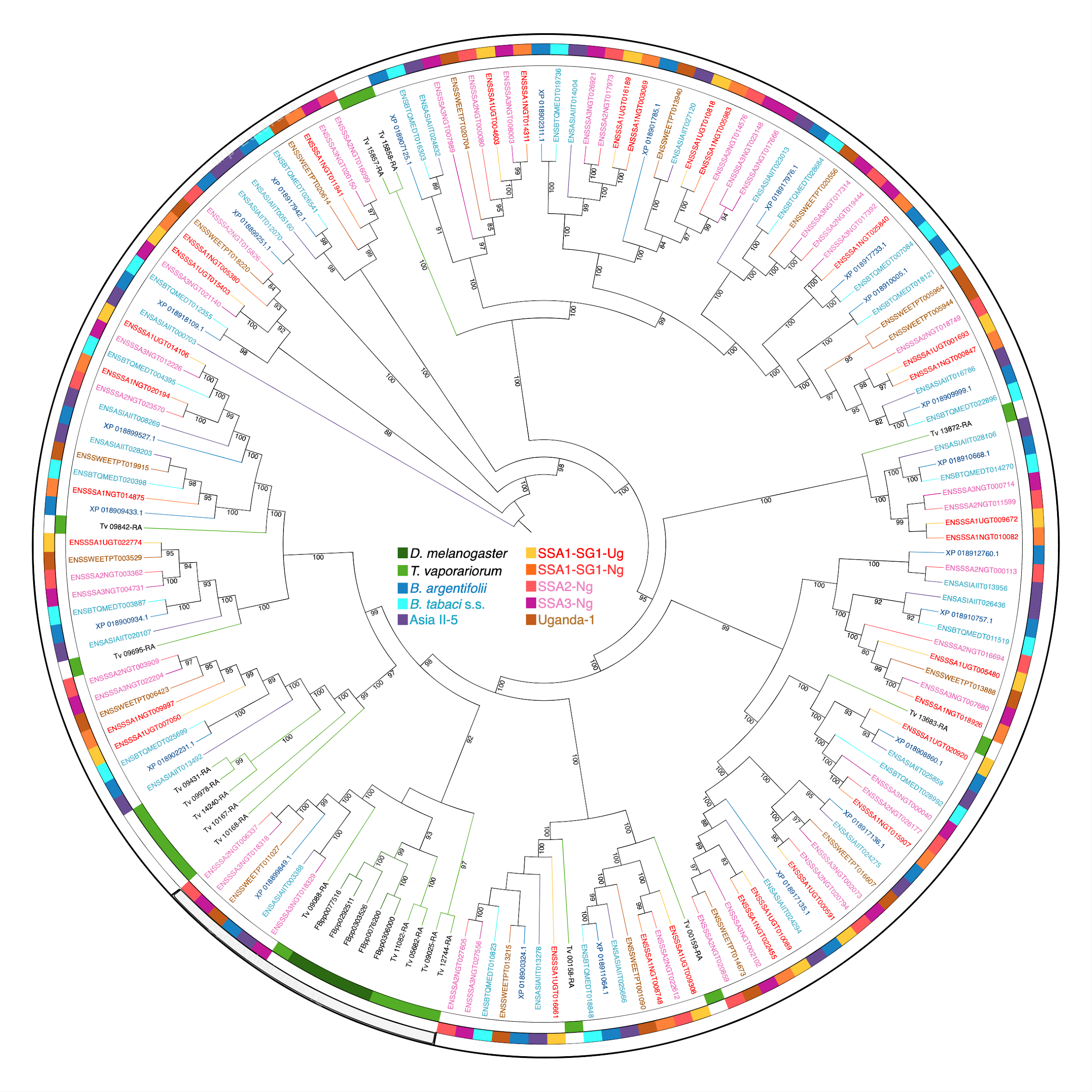
 Fig. S10: Phylogenetic analysis of carboxylesterase proteins using maximum likelihood (ML)

ML circular cladogram showing the relationships of carboxylesterase enzymes from eight *Bemisia tabaci s.l.* species, *Trialeurodes vaporariorum*, and *Drosophila melanogaster*. The tree was inferred using a total of 23 sequences from the reference *B. tabaci s.l.* species (*B. argentifolii*) and the identified orthologs in the other nine species. Nodes corresponding to bootstrap replicate values between 80 to 100 are presented. The names of the carboxylesterase proteins from *B. argentifolii* are highlighted in blue, *B. tabaci s.s.* and Asia II-5 are highlighted in light-blue, species in the SSA1-SG1∪G/1NG biological group in red, species in the SSA2∪SSA3 biological groups in pink, and Uganda-1 in brown. *Trialeurodes vaporariorum* and *D. melanogaster* are indicated by light and dark green, respectively. Colors surrounding the cladogram indicate the corresponding species: blue for *B. argentifolii*, light blue for *B. tabaci s.s.*, purple for Asia II-5, yellow for SSA1-SG1-Ug, orange for SSA1-SG1-Ng, red for SSA2-Ng, dark pink for SSA3-Ng and brown for Uganda-1. The outer circles specify the subfamily of the corresponding carboxylesterase enzymes (a/b esterase and one COE-1E). Protein sequences are denoted using GenBank accession numbers or Ensembl-EBI ‘stable Identifier’ (‘ENSXXX’) linked to the genomes presented in this study. Species name abbreviations are as indicated: XP; *B. argentifolii*, ENSSSA1NG; SSA1-SG1-Ng, ENSSSA1UG; SSA1-SG1-Ug, ENSSSA2NG; SSA2-Ng, ENSSSA3NG; SSA3-Ng, ENSASIAII; Asia II-5, ENSSSWEETP; Uganda-1; Tv; *T. vaporariorum* and FBpp; *D. melanogaster* (for more details see Additional file 1: Table S12).

##
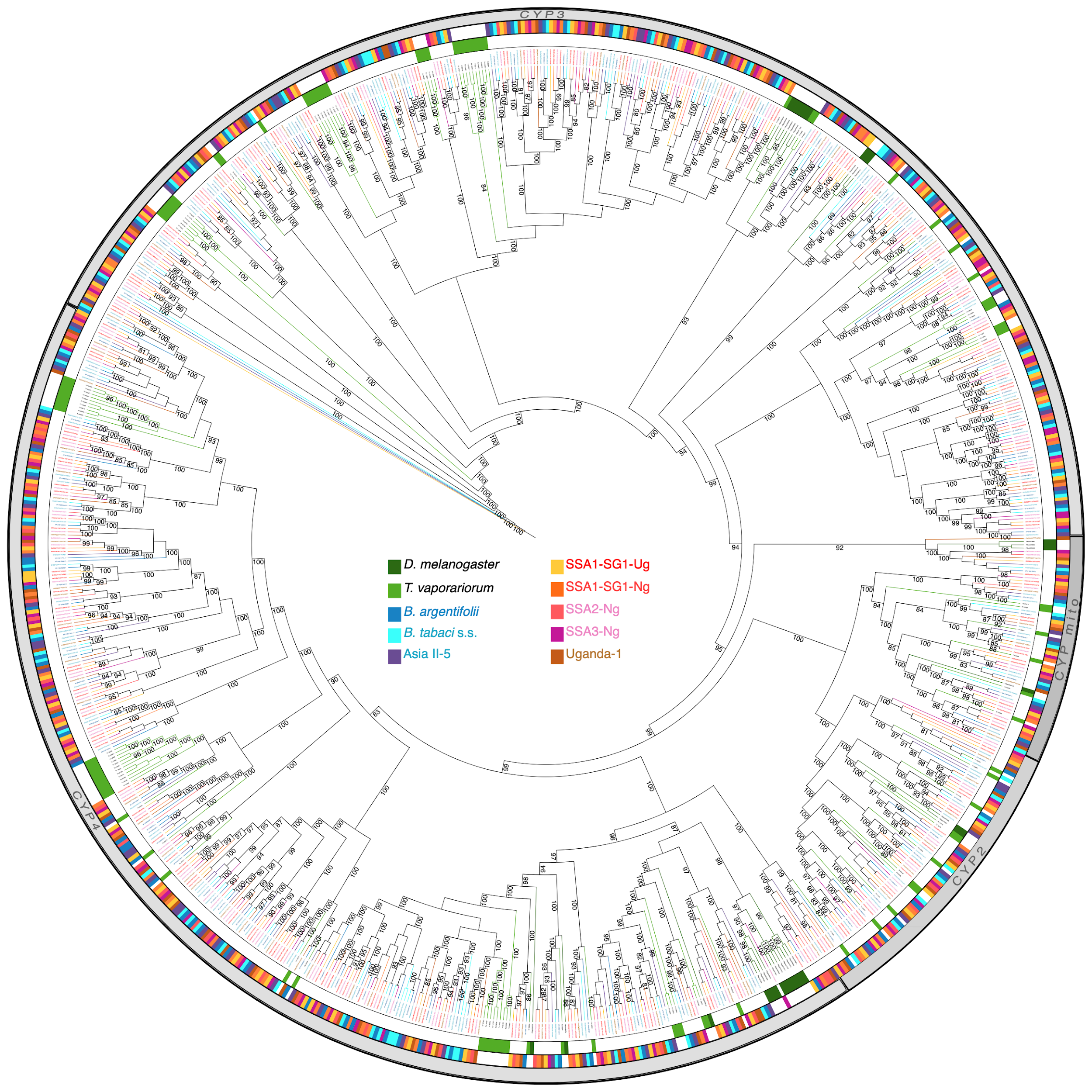


## Fig. S11: Phylogenetic analysis of cytochrome P450 proteins using maximum likelihood (ML)

ML circular cladogram showing the relationships of cytochrome P450 enzymes from eight *Bemisia tabaci s.l.* species, *Trialeurodes vaporariorum*, and *Drosophila melanogaster*. The tree was inferred using a total of 120 sequences of the reference *B. tabaci* species (*B. argentifolii*) and the identified orthologs in the other nine species. Nodes corresponding to bootstrap replicate values between 80 to 100 are presented. The names of the P450 proteins from *B. argentifolii* are highlighted in blue, *B. tabaci s.s.* and Asia II-5 are highlighted in light-blue, species in the SSA1-SG1∪G/1NG biological group in red, species in the SSA2∪SSA3 biological groups in pink, and Uganda-1 in brown. *Trialeurodes vaporariorum* and *D. melanogaster* are indicated by light and dark green, respectively. Colors surrounding the cladogram indicate the corresponding species: blue for *B. argentifolii*, light blue for *B. tabaci s.s.*, purple for Asia II-5, yellow for SSA1-SG1-Ug, orange for SSA1-SG1-Ng, red for SSA2-Ng, dark pink for SSA3-Ng and brown for Uganda-1. The outer circles specify the subfamily of the corresponding P450 enzymes (CYP2, CYP3, CYP4, and mitochondrial). Protein sequences are denoted using GenBank accession numbers or Ensembl-EBI ‘stable Identifiers’ (‘ENSXXX’) linked to the genomes presented in this study. Species name abbreviations are as indicated: XP; *B. argentifolii*, ENSSSA1NG; SSA1-SG1-Ng, ENSSSA1UG; SSA1-SG1-Ug, ENSSSA2NG; SSA2-Ng, ENSSSA3NG; SSA3-Ng, ENSASIAII; Asia II-5, ENSSSWEETP; Uganda-1; Tv; *T. vaporariorum* and FBpp; *D. melanogaster* (for more details see (Additional file 1: Table S12).


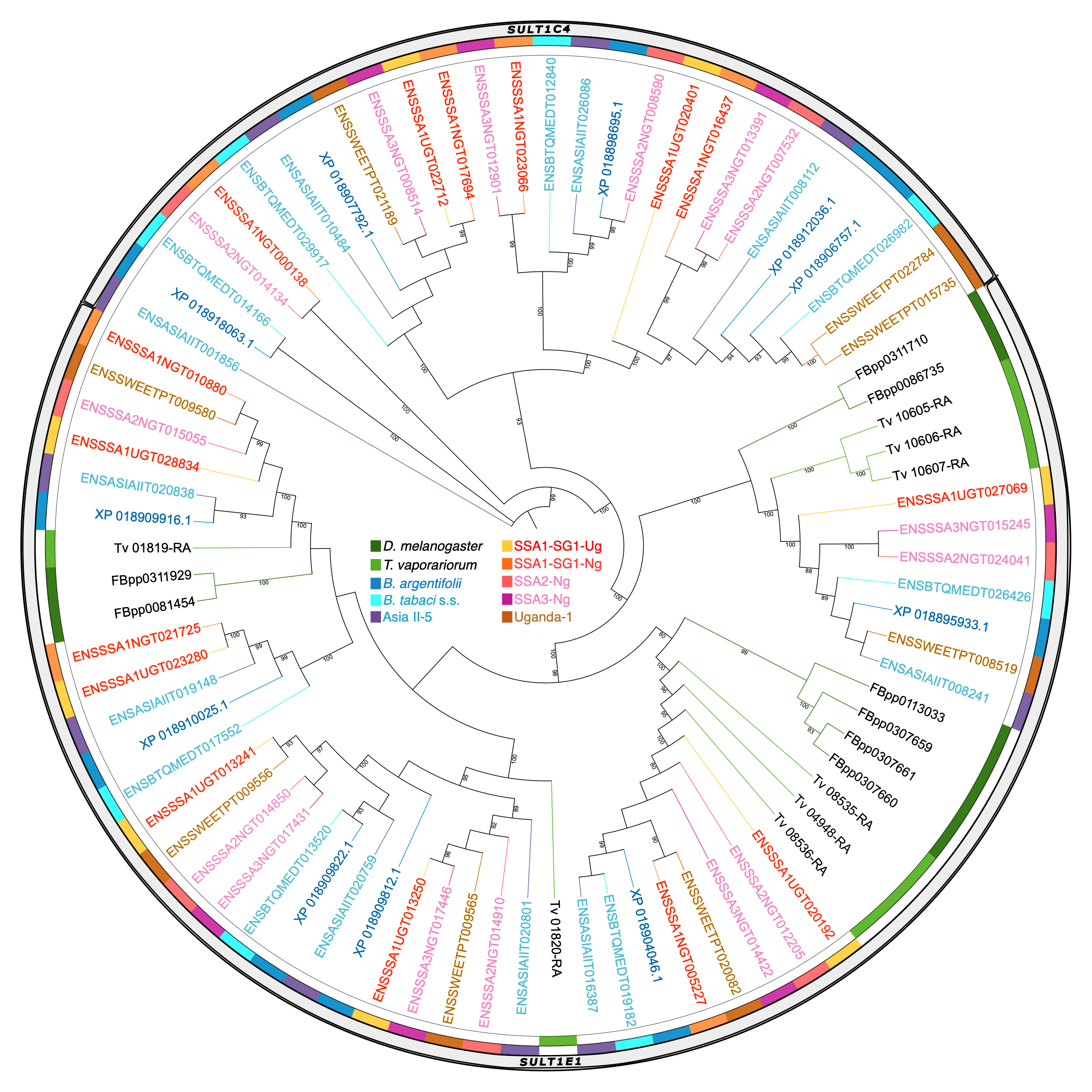


## Fig. S12: Phylogenetic analysis of sulfotransferases using maximum likelihood (ML)

ML circular cladogram showing the relationships of sulfotransferase enzymes from eight *Bemisia tabaci s.l.* species, *Trialeurodes vaporariorum*, and *Drosophila melanogaster*. The tree was inferred using a total of 11 sequences of the reference *B. tabaci* species (*B. argentifolii*) and the identified orthologs in the other nine species. Nodes corresponding to bootstrap replicate values between 80 to 100 are presented. The names of the sulfotransferase proteins from *B. argentifolii* are highlighted in blue, *B. tabaci s.s.* and Asia II-5 are highlighted in light-blue, species in the SSA1-SG1∪G/1NG biological group in red, species in the SSA2∪SSA3 biological groups in pink, and Uganda-1 in brown. *Trialeurodes vaporariorum* and *D. melanogaster* are indicated by light and dark green, respectively. Colors surrounding the cladogram indicate the corresponding species: blue for *B. argentifolii*, light blue for *B. tabaci s.s*., purple for Asia II-5, yellow for SSA1-SG1-Ug, orange for SSA1-SG1-Ng, red for SSA2-Ng, dark pink for SSA3-Ng and brown for Uganda-1. The outer circles specify the subfamily of the corresponding sulfotransferase enzymes (SULT1E1 and SULT1C4). Protein sequences are denoted using GenBank accession numbers or Ensembl-EBI ‘stable Identifiers’ (‘ENSXXX’) linked to the genomes presented in this study. Species name abbreviations are as indicated: XP; *B. argentifolii*, ENSSSA1NG; SSA1-SG1-Ng, ENSSSA1UG; SSA1-SG1-Ug, ENSSSA2NG; SSA2-Ng, ENSSSA3NG; SSA3-Ng, ENSASIAII; Asia II-5, ENSSSWEETP; Uganda-1; Tv; *T. vaporariorum* and FBpp; *D. melanogaster* (for more details see Additional file 1: Table S12).

##
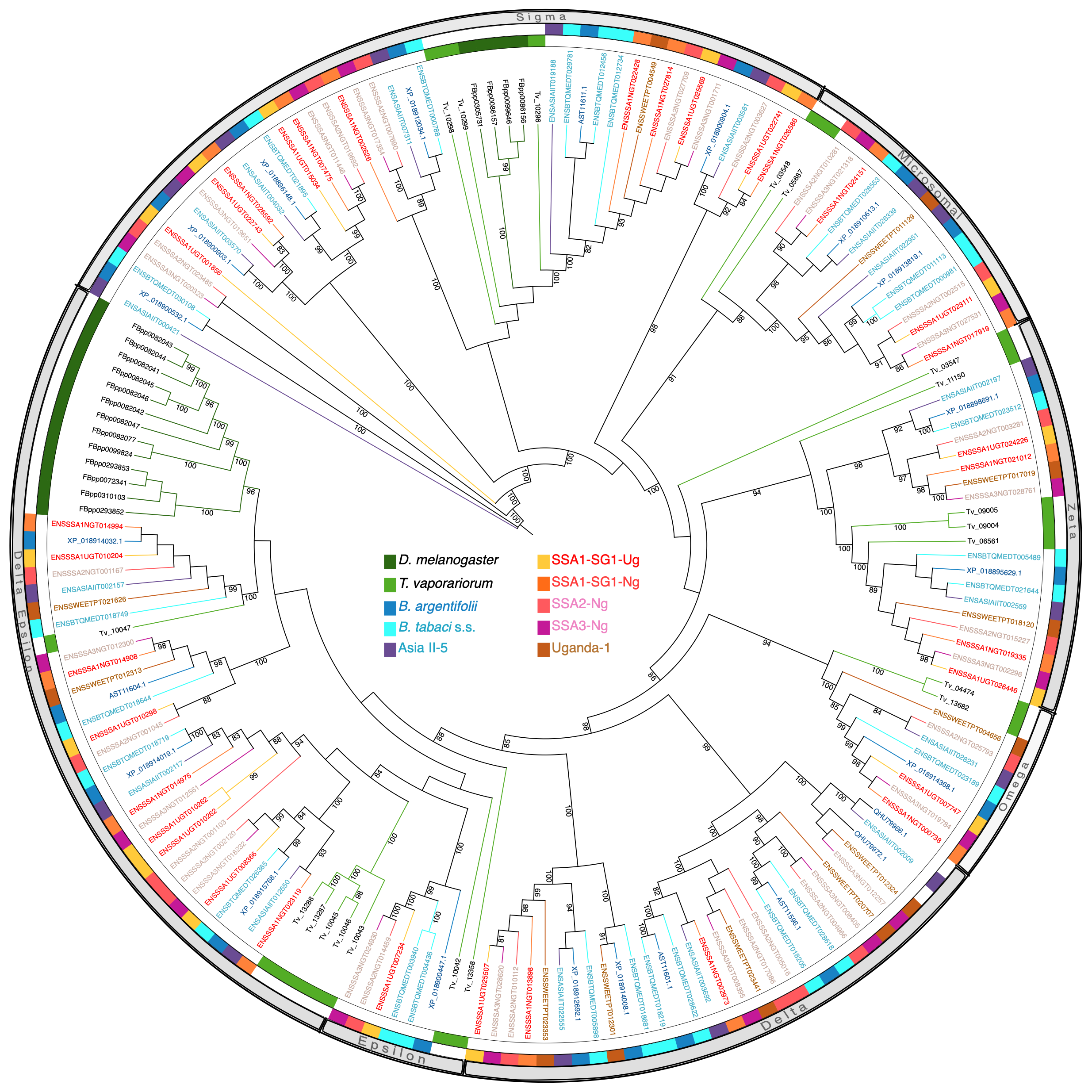


## Fig. S13: Phylogenetic analysis of GSTs using maximum likelihood (ML)

ML circular cladogram showing the relationships of glutathione S-transferases (GSTs) enzymes from eight *Bemisia tabaci s.l.* species, *Trialeurodes vaporariorum* (*T. vaporariorum*), and *Drosophila melanogaster* (*D. melanogaster*). The tree was inferred using a total of 22 sequences of the reference *B. tabaci* species (*B. argentifolii*) and the identified orthologs in the other nine species. Nodes corresponding to bootstrap replicate values between 80 to 100 are presented. The names of the GST proteins from *B. argentifolii* are highlighted in blue, *B. tabaci s.s.* and Asia II-5 are highlighted in light-blue, species in the SSA1-SG1∪G/1NG biological group in red, species in the SSA2∪SSA3 biological groups in pink, and Uganda-1 in brown. *Trialeurodes vaporariorum* and *D. melanogaster* are indicated by light and dark green, respectively. Colors surrounding the cladogram indicate the corresponding species: blue for *B. argentifolii*, light blue for *B. tabaci s.s.*, purple for Asia II-5, yellow for SSA1-SG1-Ug, orange for SSA1-SG1-Ng, red for SSA2-Ng, dark pink for SSA3-Ng and brown for Uganda-1. The outer circles specify the subfamily of the corresponding GST enzymes (Delta, Epsilon Theta, Omega, Zeta, Sigma, Delta-Epsilon, and microsomal). Protein sequences are denoted using GenBank accession numbers or Ensembl-EBI ‘stable Identifiers’ (‘ENSXXX’) linked to the genomes presented in this study. Species name abbreviations are as indicated: XP; *B. argentifolii*, ENSSSA1NG; SSA1-SG1-Ng, ENSSSA1UG; SSA1-SG1-Ug, ENSSSA2NG; SSA2-Ng, ENSSSA3NG; SSA3-Ng, ENSASIAII; Asia II-5, ENSSSWEETP; Uganda-1; Tv; *T. vaporariorum* and FBpp; *D. melanogaster* (for more details see Additional file 1: Table S12).

##
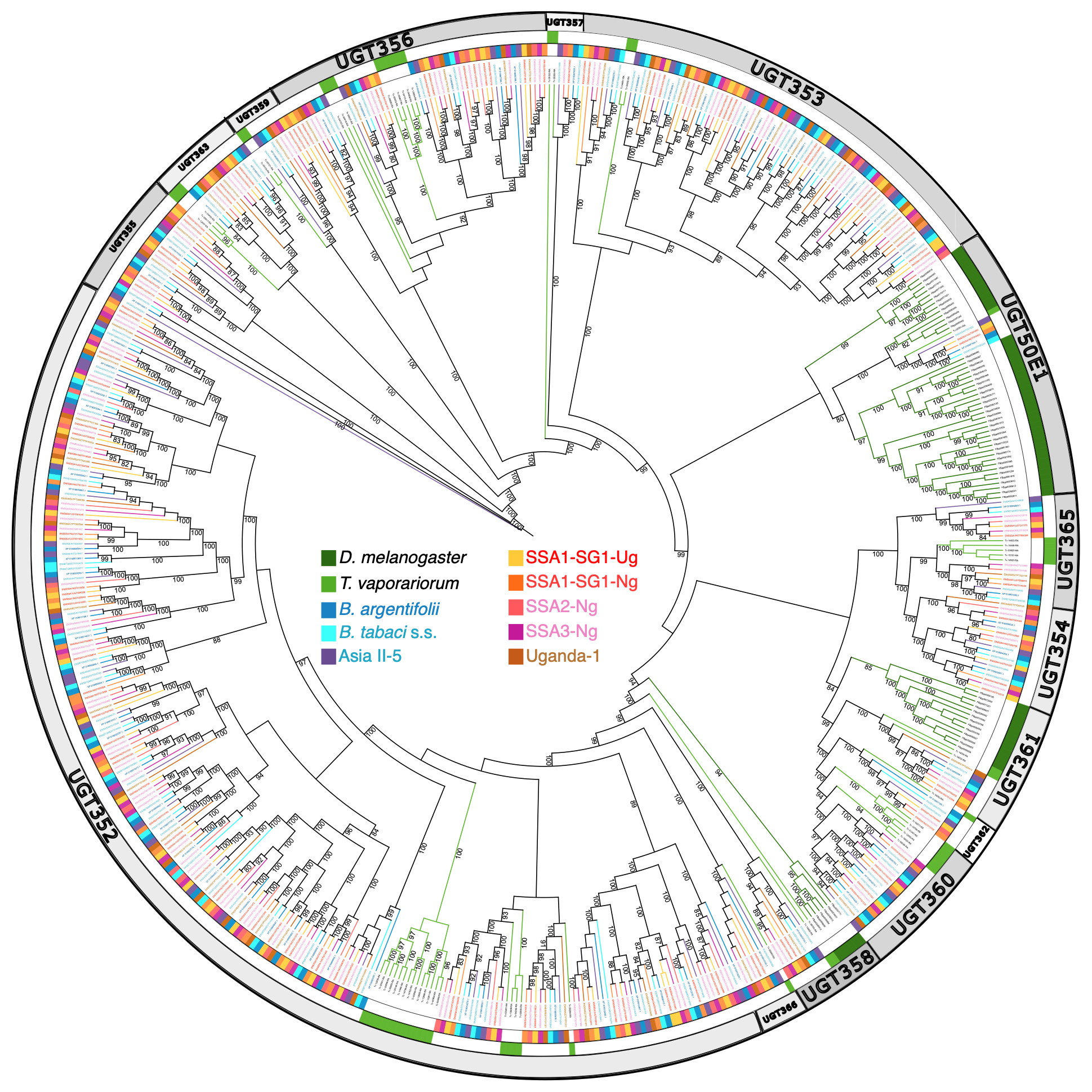


## Fig. S14: Phylogenetic analysis of UGTs using maximum likelihood (ML)

ML circular cladogram showing the relationships of uridine diphosphate-glucuronosyltransferase (UGTs) enzymes from eight *Bemisia tabaci s.l.* species, *Trialeurodes vaporariorum* (*T. vaporariorum*), and *Drosophila melanogaster* (*D. melanogaster*). The tree was inferred using a total of 72 sequences of the reference *B. tabaci* species (*B. argentifolii*) and the identified orthologs in the other nine species. Nodes corresponding to bootstrap replicate values between 80 to 100 are presented.. The names of the UGTs proteins from *B. tabaci* and *B. argentifolii* are highlighted in blue, *B. tabaci s.s.* and Asia II-5 are highlighted in light blue, species in the SSA1-SG1∪G/1NG biological group in red, species in the SSA2∪SSA3 biological groups in pink, and Uganda-1 in brown. *Trialeurodes vaporariorum* and *D. melanogaster* are indicated by light and dark green, respectively. Colors surrounding the cladogram indicate the corresponding species: blue for *B. argentifolii*, light blue for *B. tabaci s.s.*, purple for Asia II-5, yellow for SSA1-SG1-Ug, orange for SSA1-SG1-Ng, red for SSA2-Ng, dark pink for SSA3-Ng and brown for Uganda-1. The outer circles specify the subfamily of the corresponding UGTs enzymes (UGT352, UGT353, UGT354, UGT355, UGT356, UGT357, UGT358, UGT359, UGT360, UGT361, UGT362, UGT363, UGT365, UGT366, UGT50). Protein sequences are denoted using GenBank accession numbers or Ensembl-EBI ‘stable Identifiers’ (‘ENSXXX’) linked to the genomes presented in this study. Species name abbreviations are as indicated: XP; *B. argentifolii*, ENSSSA1NG; SSA1-SG1-Ng, ENSSSA1UG; SSA1-SG1-Ug, ENSSSA2NG; SSA2-Ng, ENSSSA3NG; SSA3-Ng, ENSASIAII; Asia II-5, ENSSSWEETP; Uganda-1; Tv; *T. vaporariorum* and FBpp; *D. melanogaster* (for more details see Additional file 1: Table S12).


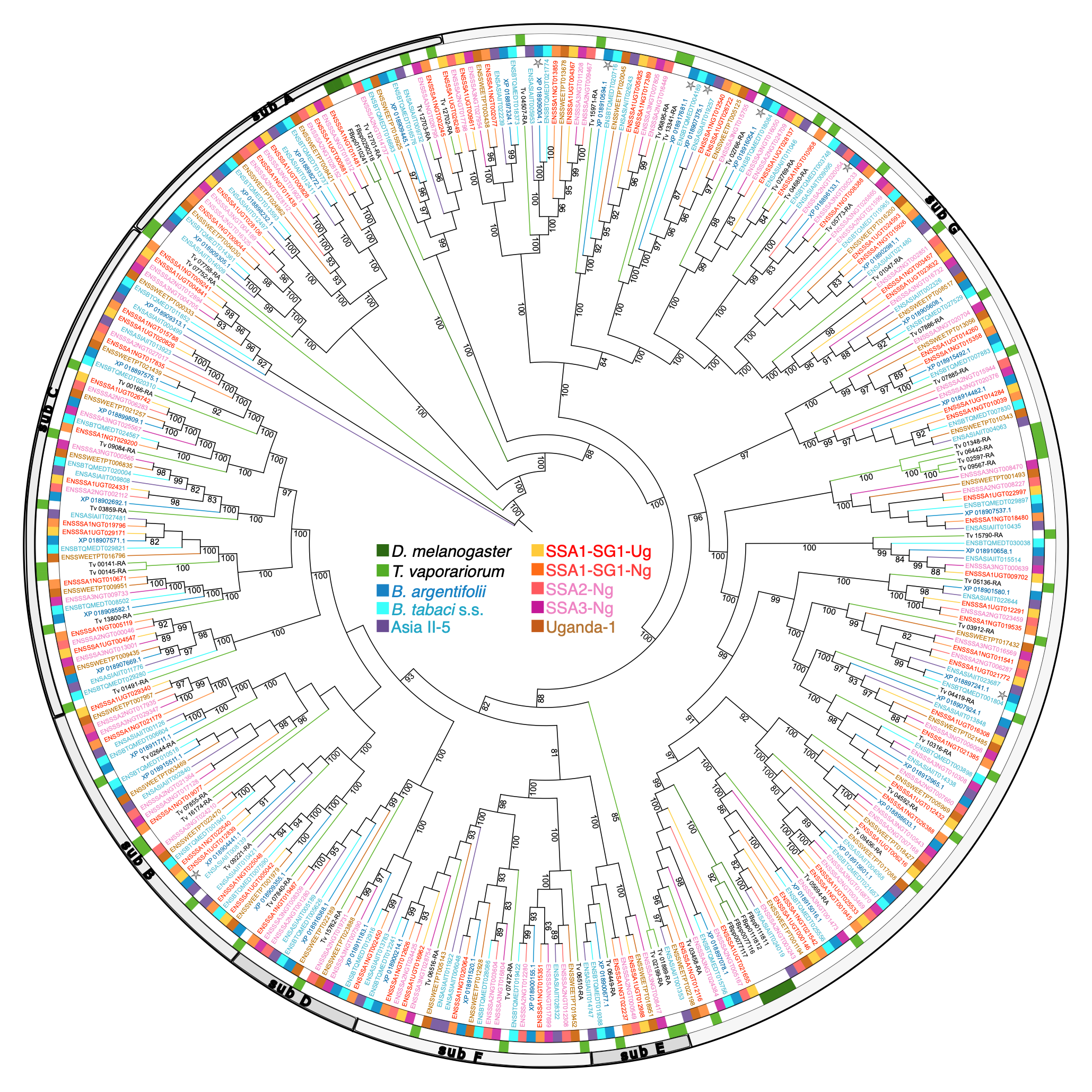


## Fig. S15: Phylogenetic analysis of ABC transporter proteins using maximum likelihood (ML)

ML circular cladogram showing the relationships of ABC (ATP-binding cassette) transporter enzymes from eight *Bemisia tabaci s.l.*  species, *Trialeurodes vaporariorum* (*T. vaporariorum*), and *Drosophila melanogaster* (*D. melanogaster*). The tree was inferred using a total of 42 sequences of the reference *B. tabaci s.l.* species (*B. argentifolii*) and the identified orthologs in the other nine species. Nodes corresponding to bootstrap replicate values between 80 to 100 are presented. The names of the ABC transporters proteins from *B. argentifolii* are highlighted in blue, *B. tabaci s.s.* and Asia II-5 are highlighted in light blue, species in the SSA1-SG1∪G/1NG biological group in red, species in the SSA2∪SSA3 biological groups in pink, and Uganda-1 in brown. *Trialeurodes vaporariorum* and *D. melanogaster* are indicated by light and dark green, respectively. Colors surrounding the cladogram indicate the corresponding species: blue for *B. argentifolii*, light blue for *B. tabaci s.s.*, purple for Asia II-5, yellow for SSA1-SG1-Ug, orange for SSA1-SG1-Ng, red for SSA2-Ng, dark pink for SSA3-Ng and brown for Uganda-1. The outer circles specify the subfamily of the corresponding ABC transporters enzymes (ABCA, ABCB, ABCC, ABCD, ABCE, ABCF and ABCG). Protein sequences are denoted using GenBank accession numbers or Ensembl-EBI ‘stable Identifiers’ (‘ENSXXX’) linked to the genomes presented in this study. Species name abbreviations are as indicated: XP; *B. argentifolii*, ENSSSA1NG; SSA1-SG1-Ng, ENSSSA1UG; SSA1-SG1-Ug, ENSSSA2NG; SSA2-Ng, ENSSSA3NG; SSA3-Ng, ENSASIAII; Asia II-5, ENSSSWEETP; Uganda-1; Tv; *T. vaporariorum* and FBpp; *D. melanogaster* (for more details see Additional file 1: Table S12).

##
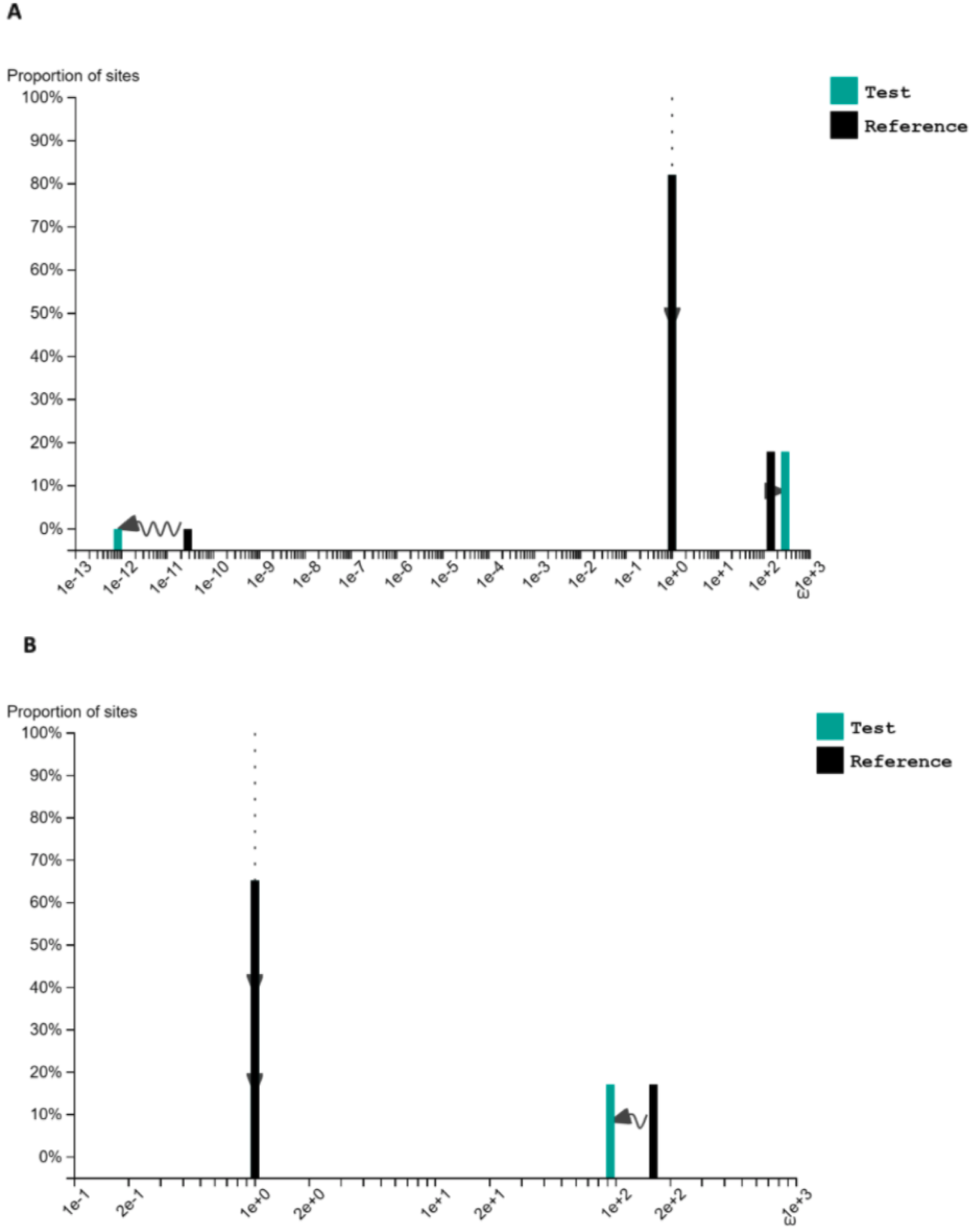


## Fig. S16: Omega plot showing ω distribution under the RELAX alternative model of SSA1-SG1-Ug α-glucosidase (GH13) genes

Part (A) shows cluster C1 and part (B) shows cluster C2 (sucrose hydrolysis) as test branches while all other SSA1-SG1-Ug α-glucosidase were considered as reference branches. Plot shows selection on test branches in cluster C1 was intensified (overall branch specific relaxation parameter (K) = 1.14) while selection on genes in cluster C2 was relaxed (K = 0.89), with test branches shifting towards neutrality.

##
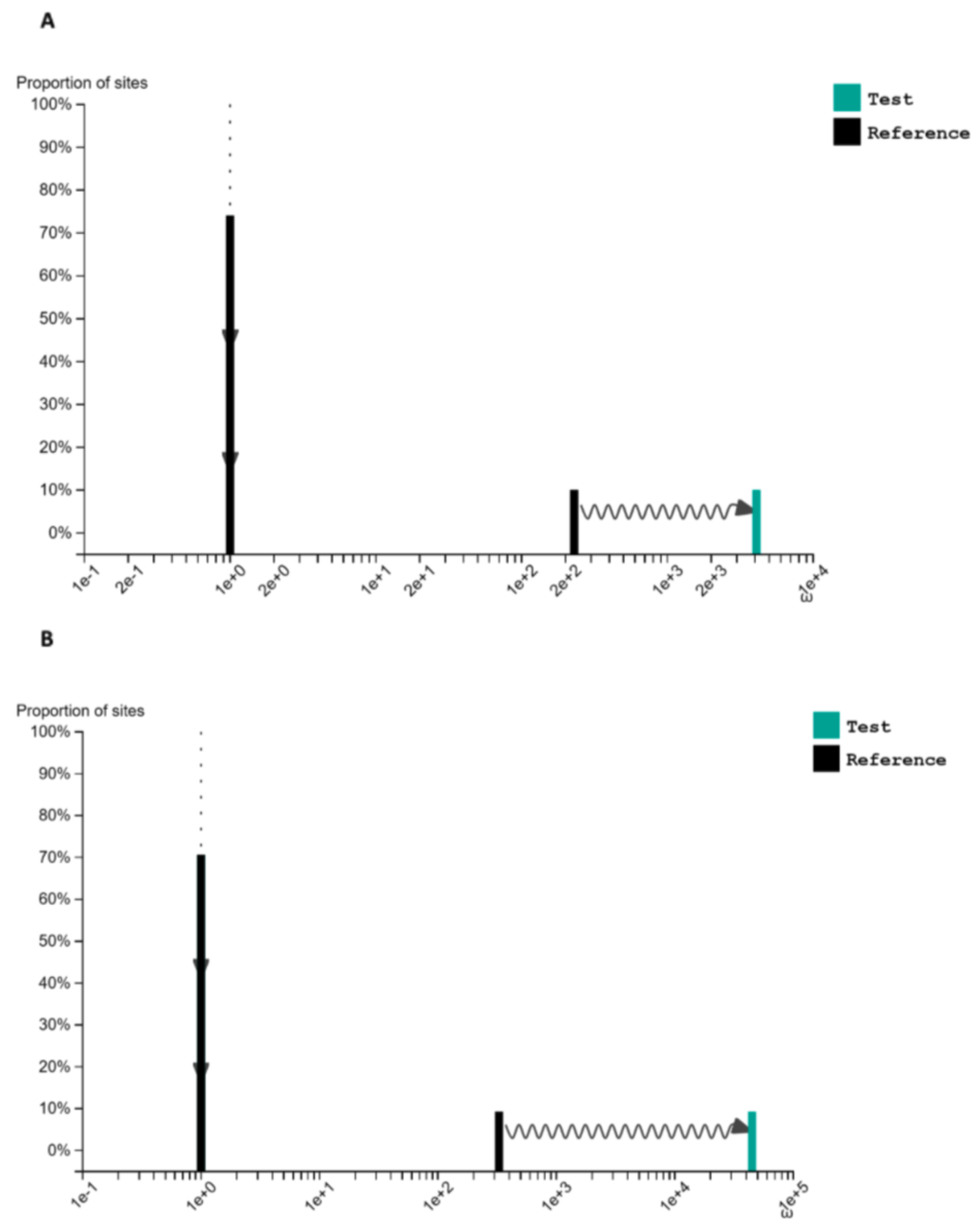


## Fig. S17: Omega plot showing ω distribution under the RELAX alternative model with SSA1-SG1-Ng α-glucosidase (GH13) genes

Part (A) shows cluster C1 and part (B) shows cluster C2 (sucrose hydrolysis) as test branches while all other SSA1-SG1-Ng α-glucosidase were considered as reference branches. Plot shows selection on test branches in both C1 and C2 gene clusters was intensified (K =1.53 & K = 1.85 respectively), with test branches shifting away from neutrality.
