# Supplementary material for: Comparative evolutionary analyses of eight whitefly Bemisia tabaci sensu lato genomes: cryptic species, agricultural pests and plant-virus vectors
Source: BMC Genomics. 2023 Jul 19;24:408. doi: 10.1186/s12864-023-09474-3 (PMC10357772; doi:10.1186/s12864-023-09474-3)
Supplement: Supplementary file 4 — Additional file 4. Supplementary text. [file 12864_2023_9474_MOESM4_ESM.docx]

**Additional file 4: Supplementary text**

Comparative evolutionary analyses of eight whitefly *Bemisia tabaci sensu lato* genomes: cryptic species, agricultural pests and plant-virus vectors

Lahcen I. Campbell, Joachim Nwezeobi, Sharon L. van Brunschot, Tadeo Kaweesi, Susan E. Seal, Rekha Swamy, Annet Namuddu, Gareth L. Maslen, Habibu Mugerwa, Irina M. Armean, Leanne Haggerty, Fergal J. Martin, Osnat Malka, Diego Santos-Garcia, Ksenia Juravel, Shai Morin, Michael E. Stephens, Paul Visendi Muhindira, Paul J. Kersey, M. N. Maruthi, Christopher A. Omongo, Jesús Navas-Castillo, Elvira Fiallo-Olivé, Ibrahim Umar Mohammed, Hua-Ling Wang, Joseph Onyeka, Titus Alicai, John Colvin.

Correspondence to: [lcampbell@ebi.ac.uk](mailto:lcampbell@ebi.ac.uk); [jn11@sanger.ac.uk](mailto:jn11@sanger.ac.uk)

# Table of contents

[**Table of contents 1**](#_vj24u76fsf0c)

[Generation of isofemale lines and full-sib inbreeding 2](#_dz68pzqckjv2)

[Nucleic acid isolation and evaluation 2](#_8ghryavzkruo)

[Transcriptomic sequencing and processing 3](#_m1px7vb7fxzc)

[Genomic sequencing and processing 4](#_lhhq9im4vwcl)

[Genome assembly refinement 4](#_v3sak5vlce1g)

[Repeat library generation and TE annotation 5](#_s0we004sgn5j)

[Annotation of *B. tabaci s. l.* genomes 5](#_433awm8tt6xb)

[Target gene family identification 6](#_dhiq1yw570ke)

[Reciprocal cross-mating 7](#_iywbq3pqo300)

[Detoxification and cassava adaptation 8](#_dv61n9yrm1lz)

[α-glucosidase (GH13) phylogenetics and selection-pressure evaluation 8](#_4l7zzcbmxu3p)

[Bacterial and fungal HTG analyses 9](#_5x8msfx0dru1)

[Mitochondrial genome assembly, annotation and phylogenetics 9](#_7fi3jhh514rm)

[RFLP-based population differentiation using single copy nuclear genes 11](#_836swzub93ix)

[References 11](#_tqffjk9a4mfx)

##

## Generation of isofemale lines and full-sib inbreeding

Independent core colonies of each *B. tabaci s.l.* population used in this study were first established from field-collections of ~100-300 adults, per single host plant ([Additional file 1: Table S1](https://docs.google.com/document/d/1ycEO5DMo4oWFkaqaQzGjr_JgcLLlZ8UA_hRomjPrDII/edit#heading=h.jkbwh35o4ag)). Isofemale lines were generated from each core colony. A single adult female was collected and transferred to a small Lock&Lock pot (LLP) whitefly-proof cage [1] containing one insect free, healthy *Solanum melongena* cv. Black Beauty (eggplant) that had developed to the five-leaf stage. The female was left to oviposit for *c.* 14 days before removal from the cage to prevent copulation between the progeny and the parent. Progeny which emerged after ~22 days from the date of setup, were labeled as F1 generation isofemale lines. Virgin males and females were collected from the F1 generation isofemale lines by monitoring the emergence in each daily (from day 15), with removal of emerged adults before they had the chance to mate. On each of these days, progeny were removed in the morning as they may have already mated. The LLPs were then re-checked before noon, to collect newly emerged adults for setting up the next generation. It was expected that newly emerged female adults would not have mated within 6 hours of emergence. This strategy was adopted for obtaining virgin males and females because previous in-house observations showed that inbreeding reduced the vigor of *B. tabaci s.l.* colonies, and techniques that involved removal of leaf sections to separate emergence cages resulted in reduced survival of the subsequent generation. Collections were repeated daily until sufficient virgin pairs (one male, one female) were obtained and transferred to new LLPs containing clean and healthy eggplants at the five-leaf stage. After *c.* 14 days, adults were removed and stored in ethanol for subsequent colony purity testing (mtCO1 marker sequencing of ≥3 adults/colony). This process was repeated for each corresponding line until the F5, F6, or F8 isofemale lines were obtained (see Additional file 1: Table S1, S4).

The full-sib inbred *B. tabaci s.l.* populations were “bulked-up” to produce enough adults needed for high-molecular weight genomic DNA isolation. This was achieved by selecting one LLP with high progeny numbers and an increased female to male ratio. The adults in the selected LLP were transferred to a whitefly-proof cage (BugDorm, US) already set up with 3-6 small eggplants. The inbred adults were then allowed to mate and oviposit in the new cage until they had reached sufficient numbers for large-scale collections.

## Nucleic acid isolation and evaluation

For genomic long-read sequencing, DNA was isolated from adult haploid males (n= 1000-3000) of selected inbred *B. tabaci s.l.* colonies, full details provided in (Additional file 1: Table S1). Custom-made aspirators were used to collect individual live adults into single glass vials, which were then sexed using a stereomicroscope. Only male adults were transferred in batches to LoBind tubes (Eppendorf), and were either extracted immediately, or snap frozen using liquid nitrogen and stored at -80 °C. High molecular weight (HMW) genomic DNA was isolated using the MagAttract HMW DNA Kit (Qiagen; tissue protocol). For *B. tabaci* SSA1-SG1-Ug and Uganda-1, small batches (n=50) were extracted, with eluates pooled prior to SMRT-bell library preparation (20-30 kb) and Sequel system sequencing performed by the Earlham Institute (UK). For *B. tabaci* SSA1-SG1-Ng, SSA2-Ng, SSA3-Ng and Asia II-5, modifications to the kit procedure were employed to increase gDNA quality and yield as follows: sample batches (n=200) were extracted in double volumes, wide-bore tips were used for gDNA handling steps, two sequential elutions (50 µL buffer AE) were performed with overnight holds (4 °C) for each. In addition, eluates were further purified using the DNA Clean & Concentrator kit (Zymo Research), then pooled prior to SMRT-bell library preparation (>30 kb) and Sequel system sequencing, performed by the Centre for Genomic Research, University of Liverpool (UK). Further details are provided in Additional file 1: Table S1.

## Transcriptomic sequencing and processing

RNA was isolated from three biological replicates of each discrete developmental life stage, collected from selected isofemale *B. tabaci s.l.* colonies. Cohorts were mixed sex, unless denoted otherwise. Life stages included eggs (n=200), first instars (n=100), second instars (n=100), third instars (n=100), fourth instars (n=100), early adults (n=50, <24 hours post-emergence) and older adults (n=50, five days post-emergence). Aided by stereomicroscopy or a headband magnifying visor (3.5 x), live egg and nymphal life stages were gently collected from live whole plants using minimally destructive techniques, keeping leaves attached where possible. For this, sterile RNAse-free dissection pins were used to gently lift samples from abaxial leaf surfaces and transfer them directly to LoBind tubes (Eppendorf) held on ice. Adults were collected using custom-made aspirators directly into LoBind tubes. Samples were immediately snap frozen using liquid nitrogen and stored at -80 °C. Total RNA was isolated using the Isolate II RNA Mini Kit (Bioline), with eluates further purified using the RNA Clean & Concentrator kit (Zymo Research). Library construction and sequencing were performed by Macrogen Europe (Netherlands) and Novogene (China), full details provided in Additional file 1: Table S4.

Transcriptomic libraries were quality checked before being provided as RNA-seq data for genomic annotation. FastQC (<https://www.bioinformatics.babraham.ac.uk/projects/fastqc/>) was used for QC. Adaptor trimming was achieved with NGS-QC_Toolkit (v2.3.4.) [2] using ‘IlluQC_PRLL.pl’ (default input parameters: “5 -c 16 -l 70 -s 20 -t 1 -z t”) with adaptor file containing select sequencing adaptors (F: 5'-AATGATACGGCGACCACCGAG ATCTACACTCTTTCCCTACACGACGCTCTTCCGATCT-3'; R: 5'-GATCGGAAGAGCACACGTCTGA ACTCCAGTCACATCACGATCTCGTATGCCGTCTTCTGCTTG-3'). The NGS-QC script ‘TrimmingReads.pl’ (input parameters: “-q 20 -n 50 -l 7”) removed bases from the 3’ end of the read when PHRED < 20, trim the first 7 bases from the 5’ end and remove reads <50 nt. See Additional file 1: Table S4 for the accession of ENA-deposited RNA-seq data utilized in this study.

## Genomic sequencing and processing

ENA (European Nucleotide Archive) accessions associated with utilized PacBio read data are listed in Additional file 1: Table S1. SSA1-SG1-Ug (SMRT cells n = 11) and Uganda-1 (SMRT cells n = 7) PacBio CLR libraries were obtained from Earlham Institute (<https://www.earlham.ac.uk/>) then processed using a combination of an in-house custom unix script (available upon request) and the software suite SMRT-Analysis v5.0 smrtlink-release: 5.0.1.9585 (PacBio). PacBio CLR libraries for SSA1-SG1-Ng (SMRT cell n = 8), SSA2-Ng (SMRT cell n = 8) and SSA3-Ng (SMRT cell n = 8) and Asia II-5 (SMRT cell n = 8) were obtained from the Centre for Genomic Research, University of Liverpool (<https://www.liverpool.ac.uk/genomic-research/>) with no additional preprocessing required prior to downstream analysis.

Kraken2 [3,4] was used to analyze genomic read data to identify potential sources of contamination and perform filtration prior to genome assembly. Custom Kraken2 databases were generated consisting of candidate contaminant organisms with respect to whitefly. The Kraken2 Perl script “download_{‘Life_*Domain’}*.pl” automated download of NCBI RefSeq taxonomic meta information and genomic sequence data across bacteria, fungi and viruses. Genomes were downloaded and combined with additional RefSeq data from invertebrates and plants (Bacteria (n= 11,178), Virus (n= 7,855), Fungi (n=9), Plant (n=106) and Invertebrate (n=170)). Sources of human contamination were screened using the *homo sapien* reference genome “GCRh38.p12” (GCF_000001405.38). Lastly, a single representative genome of the *Bemisia* genus was downloaded from NCBI RefSeq; the genome of *Bemisia argentifolii* (GCF_001854935.1) which served as a positive control for identifying *Bemisia tabaci s.l.* derived read data. For details of contamination filtration see Additional file 2: Fig. S1, Additional file 1: Table S2.

## Genome assembly refinement

Initial de novo draft assemblies were produced using Canu v1.8 [5]. Intermediate assemblies were consensus polished using the long read pairwise aligner minimap2 (using ‘-x map-pb’ ) [6] and the consensus method ‘wtdbg2-cns’ adopted from the assembly software wtdbg2 [7]. Three rounds of consensus polishing were performed in total.Assembly heterogeneity was assessed by performing redundant haplotype collapsing with Redundans [8]. PBSuite-PBJelly [9] was used for genome re-scaffolding and gap-filling. Blobtools [10,11] was used to visualize taxonomic sources of any undetected contamination. All intermediate draft assemblies were assessed with Blobtools (results not shown). Any sequence taxonomically assigned to a major clade suspected of being important to aspects of whitefly biology were accepted provided mapping coverage remained high and GC content was in line with that of *Bemisia* *tabaci s.l.* Taxonomic-Coverage ‘blobplots’ can be seen in Additional file 2: Figs. S2-S7. Genome completeness was assessed throughout (Table 3) using universal single copy orthologs via BUSCO v3.0 [12,13] and OrthoDB v9 (<https://www.orthodb.org/>) lineage sets: Eukaryota (n=303), Metazoa (n=978), Arthropoda (n=1,066) and Insecta (n=1,658) (<https://busco-archive.ezlab.org/v3/>). See Additional file 1: Table S3 for further details on assembly processing and statistics of intermediate and final genome assemblies.

## Repeat library generation and TE annotation

Genomic repeat libraries were generated for all *Bemisia* s.l. genomes presented in this study via RepeatModeller --version 1.73 [14] and then filtered for any potential CDS containing repeats. A summary of repeats; repeat families and repeat sequence classification coverage was generated programmatically via parsing of the CDS-filtered RepeatModeller repeat library with custom in-house Perl script (available upon request). Genomic scale repeat masking was performed via RepeatMasker (RM) v4.1.0 [15] in combination with the transposable element (TE) database “Dfam v3.1” [16] bundled as part of RM software package. RM verbose alignment ‘.align’ files were generated and retained by calling RM with “-a” parameter.

Summary statistics and comparison of repeat content was examined further using the a Perl based RM parser “OneCodetofindthemall’” version 1.0 [17] available here: (<http://doua.prabi.fr/software/one-code-to-find-them-all>). ‘OneCode’ operates on a per query sequence basis; ideally users pass near chromosome level assembly constructs. In this study the genomes are composed of sub-chromosome scaffolds with counts ranging from 227 (Asia II-5) to 5,713 (Uganda-1) therefore additional processing was required. This was achieved with in-house generated Perl script which accepted ‘OneCode’ output files with the extension ‘.copynumber.csv’ and provided complete genome wide summary statistics for major repeat families (“DNA", "LINE", "LTR", "Low complexity", "SINE", "Satellite", "Simple repeat”, ”Unknown”).

## Annotation of *B. tabaci s. l.* genomes

Structural genomic annotation of all *B. tabaci s.l.* genomes presented herein were generated via the EMBL-EBI Ensembl Gene Annotation pipeline [18]. Each Ensembl transcript model generated was supported by experimental evidence, no PCG features are derived using ab-initio methods [18]. PCG models were identified primarily via the alignment of short-read RNA-seq data, secondary protein-to-genome alignments of proteins from whitefly or closely related species provided additional evidence and means of gene feature elongation and refinement. The Ensembl Gene Annotation pipeline gives precedence to gene models supported by well-aligned transcriptomic evidence [18,19].

The RNA-seq libraries (n=127; short-read PE Illumina) utilized in this study for genome annotation of *B. tabaci .s.l.* genomes (SSA1-SG1-Ug, SSA1-SG1-Ng, SSA2-Ng, SSA3-Ng, Asia II-5, Uganda-1) derive from ‘discrete life stage’ (n=69) or ‘insect-plant interactome’ (n=55) generated to best capture temporal expression, plant host expression differences and lowly expressed transcripts.

RNA-Seq libraries are available via the ENA (European Nucleotide archive; <https://www.ebi.ac.uk/ena>) under the parent project accessions: PRJEB35414, PRJEB28507, PRJEB36965, PRJEB35304, PRJEB39408; see Additional file 1: Table S4. The genomes of *B. argentifolii* and *B. tabaci s.s.* were reannotated (utilizing the same RNA-Seq datasets as in the original published annotations) in tandem with the six new *B. tabaci s.l.* populations. Additionally, three previously published bacteriocyte-specific RNA-seq libraries (ENA accessions: SRR1523521 (PRJNA255988); SRR835869 (PRJNA79601); SRR2001505 (PRJNA282156)) were applied in order to ensure bacteriocyte-specific gene expression contributed to structural annotation.

The Ensembl gene annotation pipeline supports gene feature identification via use of protein-to-genome alignments. Protein evidence (PE) was obtained from Uniprot (‘protein existence evidence’ criteria at the protein [PE1] or transcript level [PE2]), Swiss-Prot and TrEMBL sequences were downloaded and combined (n= 779,118). PE was used for homology verification of transcript models generated from aligned RNA-seq data restricted to sequences from species within the Order: Hemiptera (‘UniProt-2019’). Finalized genomic annotations of all six genomes generated by the Ensembl pipeline are maintained as an Ensembl ‘core’ MySQL database. Core databases include genomic annotation data stored in gene feature classes, e.g. protein-coding, pseudogenic and long/short non-coding RNAs. Finalized gene builds contain multi-transcript PCG structures, where each transcript can contain overlapping exon boundaries; excluding any redundant transcripts where the splicing pattern is completely redundant when comparing to a longer model [18]. Additionally, PCG models will have met the minimum intron threshold (10 bp) while also having a low level of repeat coverage. All alternate transcripts and canonical only transcripts (both nucleotide and amino acid) were exported via the Ensembl core API. Genomic features captured included not only PCGs, but also pseudogenes, rRNAs, lncRNAs, snoRNA, scaRNAs and other assorted misc-RNA types were also identified. Of note was the overestimation of lncRNA gene models during early annotation efforts. Recovery of PCGs improperly annotated as lncRNAs, due to insufficient protein homology evidence for *Bemisia*, was achieved by expansion of protein homology evidence using 570 experimentally verified proteins (used in gene layering and refinement) from *B. argentifolii* (provided by the whitefly community and available on request - data not shown).

## Target gene family identification

A genome wide screening approach was used to identify probable orthologs to gene families of interest and was achieved using a combination of functional protein/domain annotation obtained from InterProScan v -5.40-77.0 and Conditional reciprocal best-BLAST (CRBB) (<https://github.com/ebi-pf-team/interproscan>) [20,21]. A list of genes was composed by curation of whitefly literature on functional protein families crucial to whitefly biology and proteins previously identified in *B. argentifolii “*MEAM1.2” (e.g. ‘BtaXXXX’) were extracted and queried using the CRB-BLAST method to all *B. tabaci s.l.* CRBB target hits identified in *B. tabaci* *s. l.* were examined using standard alone BLAST against ‘*nt’* and ‘*nr’* databases and served as input to genomic screening. Both putative *B. tabaci s.l*. homologs identified via CRBB analysis and the initial *B. argentifolii* query sequences were examined for their functional protein domains via Intepro analysis and manual inspection of protein annotation using Geneious Prime 2020.1.2 (<https://www.geneious.com>).

For each gene family of interest, a list of Interpro functional protein domain IDs (e.g. “IPRXXXXXXX”) (<http://www.ebi.ac.uk/interpro/>) were used to screen the functional annotations (Interpro functional annotation ‘.tsv’ files) of gene sets of each *B. tabaci s.l.* genome. Where possible (ensuring a non-empty list), any protein domain Interpro ID deemed unspecific to the gene/gene family of interest or ubiquitously present in many other non-related gene families were not considered further. Genomic screening of protein domain IDs was achieved using a custom in-house Perl script via the Ensembl Core database API [22]. This script imported functional protein annotations i.e., curated Interpro IDs per gene family of interest and output gene counts and all associated *B. tabaci s.l.* unique Ensembl “stable ID”. Ensembl stable IDs have the format of “({ENS}, {A six letter species Tag},{G | T | P | E denoting - Gene, Transcript, Translation and Exon respectively}, {a six digit Numerical ID})” e.g. “ENSSSA1UGT000001”. Prior to inclusion of putative hits in any downstream analyses, potential hits were again manually inspected using BLAST to NCBI nt, and NCBI Ref-Seq databases to examine homology to known protein families.

## Reciprocal cross-mating

Populations were first established on fresh eggplant in whitefly-proof lock-lock pots (LLPs). Eggplant hosts were chosen for crossing experiments because the *B. tabaci s.l*. populations used in this study shared eggplant as a common host. Once progeny emerged, sterile lancets were used to cut the leaf area surrounding the red-eyed nymphs. Each leaf-disc bearing a single nymph was then transferred individually into a glass vial that was closed-off with a cotton-wool bung, ensuring only a single nymph was present. Nymphs were left in their glass vials until they metamorphosed into adults (~24 hours). Adults were then examined using a stereomicroscope to identify if it was male or female.

Each reciprocal crossing experiment consisted of three to seven replicates of two control crosses and two reciprocal crosses in both directions. Before the experiment, clean lock-lock pots (LLP) containing one-month-old eggplant were set up. The lower leaves were removed, leaving the two youngest top leaves, to reduce the leaf-area available to the whiteflies and thereby increase the chances of mating. Each LLP containing a single eggplant was set up with virgin female and male whiteflies at a ratio of 1:3, respectively in both single-pair and group mating cohorts. Group mating cohorts were made up of three females and nine males for each cross. Whiteflies in each pot were left to mate and oviposit for seven days. Any dead males were replaced with their siblings from the cohort colony. The LLPs where the female(s) died before the seventh day were discarded and discounted from future analysis. After seven days, all adults were removed from the pots and stored in 95% ethanol at -20 ^o^C. Then mtCO1 identities of the parents were checked. Once whitefly began to emerge (18 to 21 days after setting up), they were removed daily, counted, sorted by sex and stored in 95% ethanol.

Reciprocal crossing experiments were conducted to test the hypothesis that populations classified as being similar species with partial mtCO1 sequence differences under 3.5% should mate successfully and produce viable progeny. The study was done using *B. tabaci s.l.* populations from Nigeria and Uganda in allopatric and sympatric crosses. The populations originating from Nigeria (-Ng) included (i) SSA1-SG1 (SSA1-SG1-Ng), (ii) SSA2 (SSA2-Ng), and SSA3 (SSA3-Ng). The Ugandan (-Ug) populations used in this study were (i) SSA1-SG1 (SSA1-SG1-Ug) and (ii) SSA2 (SSA2-Ug). Populations were classified as sympatric if they occur alongside one another in the same field or co-occur in regions of the same country, while populations classified as allopatric were collected more than 500 miles away from each other and occur in different localities. To this end, the following population combinations were classified as sympatric (i) SSA1-SG1-Ng X SSA3-Ng, (ii) SSA2-Ug X SSA1-SG1-Ug, (iii) SSA2-Ng X SSA3-Ng and (iv) SSA1-SG1-Ng X SSA2-Ng. On the other hand, allopatric population combinations were (i) SSA1-SG1-Ng X SSA1-SG1-Ug, (ii) SSA1-SG1-Ng X SSA2-Ug and (iii) SSA2-Ug X SSA3-Ng. The parents (males and females) used in each cross were checked for their identity using the method described in [23]. The partial mtCO1 gene sequence was amplified from the DNA by PCR, using the primers described in [23]. The amplicons were then purified and sequenced. The Molecular Evolutionary Genetics Analysis (MEGA v7.0) software was used to build a phylogenetic tree with the test samples and known consensus sequences [24]. The tree was studied to observe where the test samples clustered to confirm their identity.

## Detoxification and cassava adaptation

Phylogenetic trees were constructed under a ML framework [25] for six detoxification families (COEs, P450s, SULTs, GSTs, UGTs ABCs), using the *B. argentifolii*, *B. tabaci s.s.*, Asia II-5, Uganda-1, SSA1-SG1-Ug, SSA1-SG1-Ng, SSA2-Ng and SSA3-Ng species. Ultrafast Bootstrap (BS) analysis was performed using 100 BS replicates. Two outgroup species were included in the analysis a non-*Bemisia* whitefly, *Trialeurodes vaporariorum* (greenhouse whitefly), as it is the only other Aleyrodidae species with a completely sequenced genome, and Drosophila melanogaster, as the sequences of this species are well annotated and can be used for accurate classification of homologous genes. For genome content comparisons, we documented the presence/absence of *B. argentifolii* orthologs in each of the three African grouped biological species: SSA1-SG1∪SG2, SSA2∪SSA3, and Uganda-1.

## α-glucosidase (GH13) phylogenetics and selection-pressure evaluation

A subset of taxa from the main 23-taxon whole genome comparative orthology analysis were selected, which included the proteomes of 13 insect species namely *B. tabaci s.l.* (SSA1-SG1-Ug, SSA1-SG1-Ng & *B. argentifolii*), *T. castaneum, A. pisum, A. gambiae, B. terrestris, B. mori, D. plexippus, D. pulex, D. melanogaster, R. prolixus* and *T. urticae*. Orthologs within different insect species were initially identified and categorized obtaining OGCs from our Orthofinder [26] whole genome comparative analysis. All protein sequences from orthogroup (‘OG0000016’), the orthogroup for glycosyl hydrolase Family 13 were selected for further analysis. Protein sequences were analyzed to determine they possessed a complete N and C terminus using NCBI batch CD-search tool (https://www.ncbi.nlm.nih.gov/Structure/bwrpsb/bwrpsb.cgi). SignalP Server [27] was used to ascertain whether the protein sequence had a signal peptide. Prior to phylogenetic analysis, protein sequences of the 13 insect species, including the experimentally validated sucrase *A. pisum* (SUC1) gene (Q0H3F1_ACYPI), were aligned with ClustalW [28] using default parameters. Best-fit amino acid substitution model (WAG+G) was found with Bayesian information criterion (BIC) computed using ‘MEGA X’ [29,30] and implemented via Bayesian Evolutionary Analysis Sampling Trees (BEAST version 1.10.2) [31] using 4 gamma categories and a strict molecular clock model (1.0). Speciation: Yule process model [32] was considered with uniform birth rate and inverse gamma. Markov Chain Monte Carlo (MCMC) was implemented using a chain set of 10,000,000. The generated tree was visualized using iTOL v4 [33]. Both orthologs and paralogs identified in this study were used to study putative selection pressure acting on osmoregulation genes in *B. tabaci s.l.* SSA1-SG1-Ug and SSA1-SG1-Ng.

Episodic diversifying selection was analyzed using an adaptive branch-site effects model implemented in aBSREL (adaptive Branch-Site Random Effects Likelihood) software [34]. aBSREL allows for branch-to-branch and site-to-site variation in the substitution rate, allowing each site to evolve under any omega (ω) value. For each whitefly considered, each α-glucosidase (GH13) gene was selected as the “fore-ground” branch to test for episodic positive selection. Each gene was analyzed for the number of sites with pervasive selection using Fixed Effect Likelihood (FEL) software [35]. RELAX hypothesis testing framework [36] was used to fit the selection intensity parameter (K) for each branch. A significant result of K > 1 indicated that selection strength has been intensified along the test branches conversely K < 1 indicated that selection strength has been relaxed.

## Bacterial and fungal HTG analyses

Deduced amino acid sequences of the canonical protein-coding transcripts from *B. argentifolii*, SSA1-SG1-Ug, and SSA1-SG1-Ng were analyzed using eggNOG mapper-v2 [37], which utilizes the precomputed gene clusters and phylogenies of eggNOG 5.0 [37], to predict functions and resolve taxonomic relationships. Sequences of bacterial or fungal origins were then grouped into OGCs using the OrthoVenn2 web server [38]. CRBB was used for sequence discovery, where HTGs originally published in the genome of *B. argentifolii* (78 fungal and 63 bacterial HTGs) were used as queries in the analysis. An initial low discovery rate (recovery of *Bemisia* HTGs) in early gene annotations (data not shown) and methodological differences in annotation methods utilized for the published *Bemisia* genomes, prompted us to investigate the discrepancy further. All eight genomes were carefully annotated, or re-annotated, with their relevant RNA-seq (published RNA-seq sample), in addition to the 570 experimentally verified protein sequences utilized as additional homology evidence, see above: ‘Annotation of *B. tabaci s. l.* genomes’.

## Mitochondrial genome assembly, annotation and phylogenetics

De Novo genome assembly proceeded with the utilization of PacBio long read data, and where available high throughput Illumina short reads. Paired end Illumina data for SSA1-1SG1-Ug was utilized for draft genome correction and refinement but was not performed with SSA1-SG1-Ng for which no PE Illumina data was generated in this study. SSA1-SG1-Ug short read Illumina data was deposited to ENA (Run acc: ERR4160731 / Sample acc: ERS4559785). Mitochondrial genomes were produced using “consensus corrected” PacBio reads produced by Canu’s error correction stage previously generated during whole nuclear genome assembly. The software OrganellePBA [39] was used for the draft assembly. OrganellePBA was chosen for its ability to generate complete de novo assemblies of both mitochondrial and chloroplast genomes directly from whole genome PacBio reads alone.

Some alterations were made to the SSA1-SG1-Ug input data before generating a complete draft assembly to account for differences in PacBio library performance overall read lengths and read counts. Hence, SSA1-SG1-Ug PacBio reads were first size restricted, selecting for reads <= 16Kb; while the remaining five species were assembled using the full set of Canu corrected reads. The OrganellePBA analysis was conducted using default parameters. The mitochondrial genome of *Bemisia tabaci* (AY521259.2 / NC_006279.1) was provided to OrganellePBA as a genome reference guide. Prior to genomic annotation, draft mitochondrial genomes produced by OrganellePBA were either processed via consensus draft polishing and correction (SSA1-SG1-Ug) or passed on directly for annotation (all other *B. tabaci s.l.*). Pilon v 1.22 [40] was used to generate a more accurate error corrected mitochondrial genome via two full rounds of mapping and consensus polishing. The first round of polishing entailed using BWA-SW v0.7.17-r1188 to map the original reads used to generate the de novo draft assembly to the first complete OrganellePBA draft [41]. Intermediate SAM alignment files were converted to BAM, sorted, indexed and then provided to Pilon along with the current draft to be corrected and refined. Once finished, a second round was performed using the polished draft as the input for read mapping and correction. A third round of polishing was not performed as no significant improvements were further observed compared to the draft produced in the second round of polishing.

The automatic mitochondrial annotation pipeline MITOS v2.0 [42,43] was used to identify proteins, tRNAs and rRNAs. Structural annotations were visualized and manually curated using their associated feature information (GFF3) in Geneious Prime 2020.1.2 ([https://www.geneious.com](https://www.geneious.com/)). Whole genome alignments were generated to identify missing or partial features. Whole genome alignments were generated in a pairwise fashion, aligning each draft to the single best BLAST hit obtained via BLASTn analysis to the NCBI-nt database (May 2020). For protein coding genes, feature location (ORF) information was altered when supported by high quality HSP alignments to its single best BLAST hit (KR559510.1 best HSP). This was done to obtain as much of the coding ORF without incorporation of additional stop codons or additional methionine start codons. Additionally, missing tRNA features not identified during MITOS annotation were also incorporated into the final completed annotation. Support for tRNA feature projection was deemed sufficient when supported by both conserved flanking gene order arrangements and a minimum pairwise identity >=75% across the aligned region containing the transferred tRNA feature.

The nucleotide and amino acid sequences of the 13 protein-coding genes (PCG) from the mitogenome assemblies of the six *B. tabaci s.l.* species were combined to 22 Hemiptera species and one outgroup, *Tribolium castaneum* (Additional file 1: Table S9). The program Translatorx [44] was used to run a MAFFT [45,46] amino-acid guided alignment. Resulting alignments were trimmed of poorly aligned sites using Gblocks [47]. The substitutional saturation of codons was tested using DAMBE v7.0.35 [48], which revealed that the third codon positions across all 13 PCGs were saturated. Also, ATP-8 and NAD-6 gene sequences were removed because of lack of reads for several populations. The phylogenetic tree was therefore reconstructed using only the first and second codon positions of 11 PCGs. Individual sequences were concatenated while a best fitting model was identified prior to phylogenetic tree inference using Partitionfinder 2.1.1 [49]. Bayesian Inference (BI) and Maximum likelihood (ML) phylogenetic reconstruction methods were performed using MrBayes v3.2.7 [50–52] and RAxML v8.2.12 [53], respectively. Eight simultaneous runs with four independent Markov chains were performed for one million generations with MrBayes v3.27, sampling every 1,000 generations. RAxML v8.2.12 was employed under GTRGAMMA and PROTAUTO models for the nucleotide and amino acid partitions, respectively. ML bootstrap analysis was performed with 1,000 replicates. Phylogenetic trees were visualized using Figtree and Inkscape.

## RFLP-based population differentiation using single copy nuclear genes

Single whitefly adults collected from reciprocal crosses were transferred to a clean 1.5 mL Eppendorf tube. To extract insect DNA, 50 μL of 10 % Chelex was added to the tube and the whitefly was crushed using a plastic pestle until a clear homogenized mixture was obtained. The mixture was then incubated at 56 °C for 20 minutes and further incubated at 100 °C for five minutes. The mixture was then centrifuged at 13,500 g for five minutes using the Eppendorf Centrifuge 5424 R and the supernatant was collected and stored at -20 °C until use.

The primers GCN1-F (5’-TCGGTACCGAGCAGTCTCAT-3’) and GCN1-R: (5’- ATCCTGATGCCTGCTTCACC-3’) were used to amplify the partial eIF-2-alpha kinase activator GCN1 (Accession No. XM_019046420.1) gene in reactions containing 2 μL of extracted whitefly template DNA. PCR conditions were as follows: (i) initial denaturation at 95 °C for 3 min, (ii) denaturation at 95 °C for 30 sec, (iii) annealing at 52 °C for 30 sec, (iv) extension at 72 °C for 1 min and (v) final extension at 72 °C for 10 min. The PCR amplification steps (ii-iv) were performed for 40 cycles. Amplicons were purified using the Thermo Scientific GeneJET PCR purification kit following the kit manufacturer’s protocol to remove impurities that may interfere with the digestion process.

Restriction enzyme digestions were done using the following enzymes: (i) *Xmn*I for SSA1-SG1-Ng X SSA1-SG1-Ug, and (ii) *Sml*I for SSA2-Ng X SSA3-Ng. Purified amplicons (2 μL) were added to a mixture of water (6 μL), enzyme (2 μL) and Cutsmart buffer (2 μL) in a 0.2 mL PCR strip. For *Xmn*I digestions, reactions were incubated for 3 hours at 37 °C and then inactivated for 10 minutes at 65°C. For *Sml*I digestions, reactions were incubated for 3 hours at 55 °C with no inactivation step. Agarose gel electrophoresis (2% w/v in 0.5 X Tris/borate/EDTA buffer) was used to view the PCR amplification and restriction patterns.

## References

1. Wang P, Sun D-B, Qiu B-L, Liu S-S. The presence of six cryptic species of the whitefly *Bemisia tabaci* complex in China as revealed by crossing experiments. Insect Sci. 2011;18:67–77.

2. Patel RK, Jain M. NGS QC Toolkit: a toolkit for quality control of next generation sequencing data. PLOS ONE. 2012;7:e30619.

3. Wood DE, Salzberg SL. Kraken: ultrafast metagenomic sequence classification using exact alignments. Genome Biol. 2014;15:R46.

4. Wood DE, Lu J, Langmead B. Improved metagenomic analysis with Kraken 2. Genome Biol. 2019;20:257.

5. Koren S, Walenz BP, Berlin K, Miller JR, Bergman NH, Phillippy AM. Canu: scalable and accurate long-read assembly via adaptive k-mer weighting and repeat separation. Genome Res. 2017;27:722–36.

6. Li H. Minimap2: pairwise alignment for nucleotide sequences. Bioinformatics. 2018;34:3094–100.

7. Ruan J, Li H. Fast and accurate long-read assembly with wtdbg2. Nat Methods. 2020;17:155–8.

8. Pryszcz LP, Gabaldón T. Redundans: an assembly pipeline for highly heterozygous genomes. Nucleic Acids Res. 2016;44:e113–e113.

9. English AC, Richards S, Han Y, Wang M, Vee V, Qu J, et al. Mind the gap: upgrading genomes with Pacific Biosciences RS long-read sequencing technology. PLOS ONE. 2012;7:e47768.

10. Kumar S, Jones M, Koutsovoulos G, Clarke M, Blaxter M. Blobology: exploring raw genome data for contaminants, symbionts and parasites using taxon-annotated GC-coverage plots. Front Genet. 2013;4:237.

11. Laetsch DR, Blaxter ML. BlobTools: interrogation of genome assemblies. F1000Research. 2017;6:1287.

12. Simão FA, Waterhouse RM, Ioannidis P, Kriventseva EV, Zdobnov EM. BUSCO: assessing genome assembly and annotation completeness with single-copy orthologs. Bioinformatics. 2015;31:3210–2.

13. Waterhouse RM, Seppey M, Simão FA, Manni M, Ioannidis P, Klioutchnikov G, et al. BUSCO applications from quality assessments to gene prediction and phylogenomics. Mol Biol Evol. 2018;35:543–8.

14. Flynn JM, Hubley R, Goubert C, Rosen J, Clark AG, Feschotte C, et al. RepeatModeler2 for automated genomic discovery of transposable element families. Proc Natl Acad Sci. 2020;117:9451–7.

15. Smit A, Hubley R, Green P. RepeatMasker Open-4.0. 2013–2015. 2015; Available from: http://www.repeatmasker.org/

16. Hubley R, Finn RD, Clements J, Eddy SR, Jones TA, Bao W, et al. The Dfam database of repetitive DNA families. Nucleic Acids Res. 2016;44:D81–9.

17. Bailly-Bechet M, Haudry A, Lerat E. “One code to find them all”: a Perl tool to conveniently parse RepeatMasker output files. Mob DNA. 2014;5:13.

18. Aken BL, Ayling S, Barrell D, Clarke L, Curwen V, Fairley S, et al. The Ensembl gene annotation system. Database. 2016;baw093.

19. Curwen V, Eyras E, Andrews TD, Clarke L, Mongin E, Searle SMJ, et al. The Ensembl automatic gene annotation system. Genome Res. 2004;14:942–50.

20. Aubry S, Kelly S, Kümpers BMC, Smith-Unna RD, Hibberd JM. Deep evolutionary comparison of gene expression identifies parallel recruitment of trans-factors in two independent origins of C4 photosynthesis. PLOS Genet. 2014;10:e1004365.

21. Zdobnov EM, Apweiler R. InterProScan-an integration platform for the signature-recognition methods in InterPro. Bioinformatics. 2001;17:847–8.

22. Cunningham F, Allen JE, Allen J, Alvarez-Jarreta J, Amode MR, Armean IM, et al. Ensembl 2022. Nucleic Acids Res. 2022;50:D988–95.

23. Nwezeobi J, Onyegbule O, Nkere C, Onyeka J, van Brunschot S, Seal S, et al. Cassava whitefly species in eastern Nigeria and the threat of vector-borne pandemics from East and Central Africa. Farooq S, editor. PLOS ONE. 2020;15:e0232616.

24. Kumar S, Nei M, Dudley J, Tamura K. MEGA: A biologist-centric software for evolutionary analysis of DNA and protein sequences. Brief Bioinform. 2008;9:299–306.

25. Kalyaanamoorthy S, Minh BQ, Wong TK, Von Haeseler A, Jermiin LS. ModelFinder: fast model selection for accurate phylogenetic estimates. Nat Methods. 2017;14:587–9.

26. Emms DM, Kelly S. OrthoFinder: phylogenetic orthology inference for comparative genomics. Genome Biol. 2019;20:1-14.

27. Petersen TN, Brunak S, Von Heijne G, Nielsen H. SignalP 4.0: discriminating signal peptides from transmembrane regions. Nat Methods. 2011;8:785–6.

28. Larkin MA, Blackshields G, Brown NP, Chenna R, Mcgettigan PA, McWilliam H, et al. Clustal W and Clustal X version 2.0. Bioinformatics. 2007;23:2947–8.

29. Kumar S, Stecher G, Li M, Knyaz C, Tamura K. MEGA X: molecular evolutionary genetics analysis across computing platforms. Mol Biol Evol. 2018;35:1547–9.

30. Kumar S, Stecher G, Tamura K. MEGA7: molecular evolutionary genetics analysis version 7.0 for bigger datasets. Mol Biol Evol. 2016;33:1870–4.

31. Suchard MA, Lemey P, Baele G, Ayres DL, Drummond AJ, Rambaut A. Bayesian phylogenetic and phylodynamic data integration using BEAST 1.10. Virus Evol. 2018;4:vey016.

32. Gernhard T. The conditioned reconstructed process. J Theor Biol. 2008;253:769–78.

33. Letunic I, Bork P. Interactive Tree of Life (iTOL) v4: recent updates and new developments. Nucleic Acids Res. 2019;47:W256–9.

34. Smith MD, Wertheim JO, Weaver S, Murrell B, Scheffler K, Kosakovsky Pond SL. Less is more: an adaptive branch-site random effects model for efficient detection of episodic diversifying selection. Mol Biol Evol. 2015;32:1342–53.

35. Kosakovsky Pond SL, Frost SDW. Not so different after all: a comparison of methods for detecting amino acid sites under selection. Mol Biol Evol. 2005;22:1208–22.

36. Wertheim JO, Murrell B, Smith MD, Pond SLK, Scheffler K. RELAX: detecting relaxed selection in a phylogenetic framework. Mol Biol Evol. 2015;32:820–32.

37. Huerta-Cepas J, Szklarczyk D, Heller D, Hernández-Plaza A, Forslund SK, Cook H, et al. eggNOG 5.0: a hierarchical, functionally and phylogenetically annotated orthology resource based on 5090 organisms and 2502 viruses. Nucleic Acids Res. 2019;47:D309–14.

38. Xu L, Dong Z, Fang L, Luo Y, Wei Z, Guo H, et al. OrthoVenn2: a web server for whole-genome comparison and annotation of orthologous clusters across multiple species. Nucleic Acids Res. 2019;47:W52–8.

39. Soorni A, Haak D, Zaitlin D, Bombarely A. Organelle PBA, a pipeline for assembling chloroplast and mitochondrial genomes from PacBio DNA sequencing data. BMC Genomics. 2017;18:1-8.

40. Walker BJ, Abeel T, Shea T, Priest M, Abouelliel A, Sakthikumar S, et al. Pilon: an integrated tool for comprehensive microbial variant detection and genome assembly improvement. PLOS ONE. 2014;9:e112963.

41. Li H, Durbin R. Fast and accurate long-read alignment with Burrows–Wheeler transform. Bioinformatics. 2010;26:589–95.

42. Bernt M, Donath A, Jühling F, Externbrink F, Florentz C, Fritzsch G, et al. MITOS: Improved *de novo* metazoan mitochondrial genome annotation. Mol Phylogenet Evol. 2013;69:313–9.

43. Donath A, Jühling F, Al-Arab M, Bernhart SH, Reinhardt F, Stadler PF, et al. Improved annotation of protein-coding genes boundaries in metazoan mitochondrial genomes. Nucleic Acids Res. 2019;47:10543–52.

44. Abascal F, Zardoya R, Telford MJ. TranslatorX: multiple alignment of nucleotide sequences guided by amino acid translations. Nucleic Acids Res. 2010;38:W7–13.

45. Katoh K. MAFFT: a novel method for rapid multiple sequence alignment based on fast Fourier transform. Nucleic Acids Res. 2002;30:3059–66.

46. Katoh K, Standley DM. MAFFT multiple sequence alignment software version 7: improvements in performance and usability. Mol Biol Evol. 2013;30:772–80.

47. Castresana J. Selection of conserved blocks from multiple alignments for their use in phylogenetic analysis. Mol Biol Evol. 2000;17:540–52.

48. Xia X. DAMBE7: new and improved tools for data analysis in molecular biology and evolution. Kumar S, editor. Mol Biol Evol. 2018;35:1550–2.

49. Lanfear R, Frandsen PB, Wright AM, Senfeld T, Calcott B. PartitionFinder 2: new methods for selecting partitioned models of evolution for molecular and morphological phylogenetic analyses. Mol Biol Evol. 2017;34:772–3.

50. Huelsenbeck JP, Ronquist F. MRBAYES: Bayesian inference of phylogenetic trees. Bioinformatics. 2001;17:754–5.

51. Ronquist F, Huelsenbeck JP. MrBayes 3: Bayesian phylogenetic inference under mixed models. Bioinformatics. 2003;19:1572–4.

52. Altekar G, Dwarkadas S, Huelsenbeck JP, Ronquist F. Parallel metropolis coupled Markov chain Monte Carlo for Bayesian phylogenetic inference. Bioinformatics. 2004;20:407–15.

53. Stamatakis A. RAxML version 8: a tool for phylogenetic analysis and post-analysis of large phylogenies. Bioinformatics. 2014;30:1312–3.
